# Dopamine drives persistent remodelling of the maternal brain

**DOI:** 10.1038/s41586-026-10509-4

**Published:** 2026-05-20

**Authors:** Jennifer C. O’Chan, Giuseppina Di Salvo, Ashley M. Cunningham, Sohini Dutta, Elizabeth Brindley, Benjamin H. Weekley, Winnie Chen, Rasika R. Iyer, Ethan Wan, Cindy Zhang, Naguib Mechawar, Gustavo Turecki, Ian Maze

**Affiliations:** 1 Nash Family Department of Neuroscience, Friedman Brain Institute, Icahn School of Medicine at Mount Sinai, New York, NY USA; 2 Department of Psychiatry and Neuropsychology, School for Mental Health and Neuroscience (MHeNs), Maastricht University, Maastricht, The Netherlands; 3 Department of Neurobiology, University of Alabama at Birmingham, Birmingham, AL USA; 4 Douglas Institute, Department of Psychiatry, McGill University, Montreal, Quebec Canada; 5 Department of Pharmacological Sciences, Icahn School of Medicine at Mount Sinai, New York, NY USA; 6 Howard Hughes Medical Institute, Icahn School of Medicine at Mount Sinai, New York, NY USA

**Keywords:** Molecular neuroscience, Epigenetics and behaviour

## Abstract

Pregnancy and postpartum experiences represent transformative physiological states that impose lasting demands on the maternal body and brain, resulting in lifelong neural adaptations^1–6^. However, the precise molecular mechanisms that drive these persistent alterations remain poorly understood. Here we used brain-wide transcriptomic profiling to define the molecular landscape of neuroplasticity induced by reproductive experience, identifying the dorsal hippocampal formation (dHF) as a key site of transcriptional remodelling. Combining single-cell RNA sequencing with a maternal–pup separation paradigm, we additionally found that chronic postpartum stress significantly disrupts dHF adaptations by altering dopamine dynamics, leading to changes in the dopamine-dependent histone post-translational modification, H3 dopaminylation, which causally mediates downstream alterations in gene expression and behaviour. In human dorsal subiculum, a brain structure within the dHF, we uncovered conserved patterns of parity-dependent alterations in H3 dopaminylation and transcription. We further established the sufficiency of dopamine modulation in regulating these adaptations via chemogenetic suppression of dopamine release into the dHF, which recapitulated key epigenomic and behavioural features of reproductive experience in virgin female mice. In sum, our findings establish dopamine as a central regulator of parity-induced neuroadaptations in humans and mice, revealing a fundamental transcriptional mechanism by which female reproductive experience remodels the brain to sustain long-term behavioural adaptations.

## Abstract (web summary)

Brain-wide transcriptomic profiling in mice reveals that reproductive experience remodels the maternal brain by altering dopamine dynamics in the dorsal hippocampal formation, causing dopamine-dependent histone post-translational modifications, and thereby changes in gene expression and behaviour.

## Main

Matrescence—the physical, emotional, hormonal and social transition to motherhood—is a period of profound transformation that reshapes the maternal body and brain to support pregnancy, birth and offspring care. Although extensive research has focused on acute maternal brain processes that are essential for the onset and maintenance of parenting, precisely how brain adaptations are sustained beyond the postpartum period to persistently influence behaviour remains unclear. Recent human neuroimaging studies have revealed that parity—encompassing prior pregnancies carried to term across diverse modes of conception, delivery and infant feeding—induces long-lasting alterations in brain connectivity and structure, persisting for years or even decades following birth^1–6^. Similarly, animal models of parity, which capture the composite exposures of pregnancy, parturition, lactation and maternal care (hereafter referred to as reproductive experience (RE)), demonstrate persistent alterations in synaptic remodelling, cell proliferation and behavioural outcomes^7–13^. However, the specific mechanisms within the extensive repertoire of maternal physiological changes that drive these long-lasting brain adaptations remain poorly understood.

## Sustained transcriptional and behavioural adaptations

**Fig. 1:**
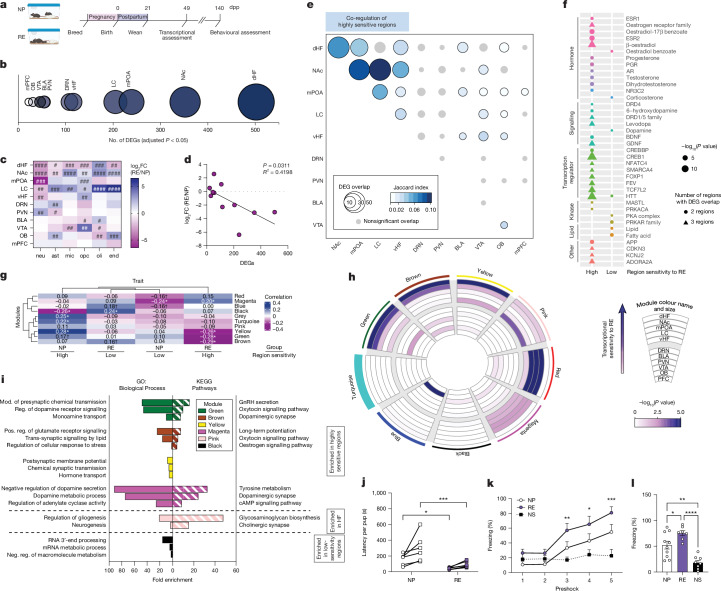
Pregnancy and postpartum experiences promote lasting transcriptional and behavioural adaptations in the maternal brain. **a**, Timeline comparing RE and NP female mice. Created in BioRender; Lab, M. https://biorender.com/mvh9p5o (2026). **b**, DEGs (from DESeq2, adjusted *P* < 0.05) per brain region. **c**, Comparison of normalized surrogate proportion variables from cell-type deconvolution analysis. Multiple unpaired two-sided Student’s *t*-tests with Benjamini–Krieger–Yekutieli correction. #FDR < 0.05, ##FDR < 0.01, ###FDR < 0.001, ####FDR < 0.0001). **d**, Linear regression of neuron proportions versus DEG number per brain region. **e**, Jaccard analysis of DEG overlap across brain regions. **f**, Upstream regulator analysis of overlapping DEGs with Ingenuity Pathway Analysis (IPA) (*P* < 0.05) for regions with high versus low sensitivity to RE. **g**, Trait heat map from weighted gene correlation network analysis showing Pearson’s correlation coefficient of each module with group × region sensitivity classification (†*P* < 0.1, *P < 0.05). **h**, Circos plot showing DEG enrichment for each gene co-expression module. Inner ring colour indicates significance of overlap (Fisher’s exact test, *P* < 0.05). Module size is indicated by arc thickness. **i**, GO and KEGG pathways enriched from gene co-expression modules (FDR < 0.05). GnRH, gonadotropin-releasing hormone; HF, hippocampal formation; mod., modulation; neg., negative; pos., positive; reg., regulation. **j**, RE (*n* = 7) and NP (*n* = 6) on the pup retrieval task. Two-way repeated measures ANOVA (rmANOVA), *P* = 0.0029, followed by Holm–Šídák multiple comparisons test. **k**, RE and NP compared to no-shock (NS) controls during contextual fear conditioning (**k**; two-way rmANOVA, *P* = 0.01, Dunnett’s multiple comparisons test) and context recall (**l**; one-way ANOVA, *P* < 0.0001, Tukey’s multiple comparisons test. Control RE (*n* = 7), NP (*n* = 10) and NS (*n* = 8). Data are mean ± s.e.m. *P < 0.05, **P < 0.01, ***P < 0.001, ****P < 0.0001. Source data

**Extended Data Fig. 1:**
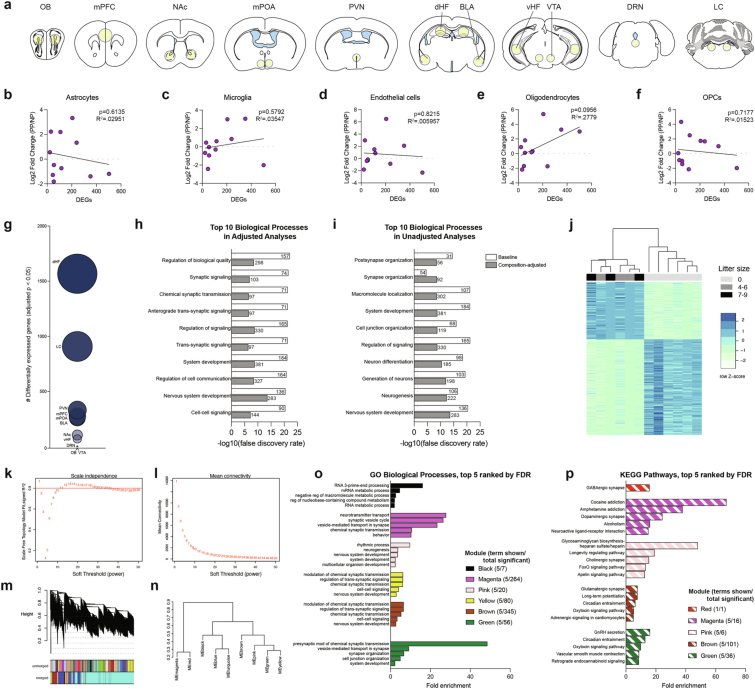
Brain-wide transcriptomic analysis of cell markers and gene expression networks. **a**) Brain regions selected for transcriptional profiling: olfactory bulb (OB), medial prefrontal cortex (mPFC), nucleus accumbens (NAc), medial preoptic area (mPOA), paraventricular nucleus (PVN), dorsal hippocampal formation (dHF), basolateral amygdala (BLA), ventral hippocampus (vHF), ventral tegmental area (VTA), dorsal raphe nucleus (DRN), and locus coeruleus (LC). Yellow circles indicate the micropunched area. Created in BioRender; Lab, M. https://biorender.com/4f8zyrj (2026). **b-f**) Linear regression analyses between fold change in normalized surrogate proportion variables generated from cell-type deconvolution analyses for **b**) astrocytic, **c**) microglia, **d**) endothelial, **e**) mature oligodendrocyte, and **f**) oligodendrocyte precursor cell markers with the number of DEGs identified from each brain region. **g**) DEGs per brain region following adjustment for cell composition changes (p_adj_ <0.05). **h-i**) Enriched GO Biological Processes in dHF between NP *vs*. RE, ranked by top 10 FDR values, for (**h**) baseline unadjusted analyses and (**i**) cell composition-adjusted analyses; with the number of genes in each GO term indicated. **j**) Heatmap of dHF DEGs between NP *vs*. RE, stratified by litter size. **k**) Scale independence plot for soft-threshold power selection for WGCNA analysis, illustrating the relationship between β and the scale-free topology fit index, identifying the optimal β value = 12. **l**) Mean connectivity plot illustrating the average network density for each evaluated soft-thresholding power. **m**) Dendrogram of gene clustering based on topological overlap. **n**) Dendrogram of eigengene network adjacency depicting similarity of modules. **o-p**) Significantly enriched **o**) GO Biological Processes and **p**) KEGG pathways, sorted by FDR values, from module gene sets. Bars are color-coded to indicate modules. The total number of significant terms for each analysis is provided. Source data

To investigate the persistent effects of RE in maternal brain, we compared dams that experienced a composite of reproductive and maternal experiences (breeding, pregnancy, parturition, lactation and pup interactions) to age-matched virgin nulliparous (NP) mice (Fig. 1a). As hormones return to baseline levels around 7 weeks post-conception^14^, we conducted brain-wide transcriptomic profiling 4 weeks following pup weaning (49 days postpartum (dpp)). Following differential expression analyses of 11 brain regions selected on the basis of prior evidence supporting their involvement in maternal behaviours (Extended Data Fig. 1a), our data revealed a wide range in the number of differentially expressed genes (DEGs; adjusted *P* < 0.05) across brain regions (Fig. 1b and Supplementary Table 1).

To assess whether these differential expression patterns could be attributed to common changes in cell types, we performed cell-type deconvolution analyses. We observed changes in cell marker expression across brain regions, regardless of the number of DEGs (Fig. 1c). Notably, the expression of neuronal markers was downregulated in the brain regions displaying the most DEGs (Fig. 1c,d and Extended Data Fig. 1b–f), implicating changes in neuronal populations that may underlie observed transcriptional responses.

As DEG differences across brain regions are influenced by cell-type composition, we repeated our analyses with surrogate cell-type proportion variables included in the model. Incorporating these covariates altered the relative ordering of brain regions by DEG number (Extended Data Fig. 1g and Supplementary Table 2). Remarkably, however, the dHF remained the most transcriptionally sensitive region, demonstrating that RE induces robust cell-intrinsic transcriptional remodelling in this region. Consistent with this, pathway enrichment analyses comparing DEGs from the original and composition-adjusted dHF analyses revealed strong concordance in the biological processes identified, indicating that both approaches robustly capture core transcriptional programs engaged by RE (Extended Data Fig. 1h,i). Notably, variation in litter size greater than zero did not meaningfully alter gene expression patterns within RE (Extended Data Fig. 1j).

We next explored whether the transcriptomic alterations in RE-sensitive regions might be driven by common upstream regulators. We returned to our unadjusted model, which preserves the full extent of RE-dependent transcriptional changes, to compare DEGs across brain regions, enabling the identification of common gene sets altered by RE. This revealed that the greatest overlap occurs in regions displaying the highest levels of transcriptional sensitivity (Fig. 1e). Next, we grouped the 11 brain regions according to the number of DEGs, the degree of overlap with other regions, and significant fold changes in neuronal marker expression, based on the premise that these regions may be co-regulated to elicit convergent transcriptional responses (referred to as high-sensitivity regions: dHF, nucleus accumbens (NAc), medial preoptic area (mPOA), locus coeruleus (LC), ventral hippocampus (vHF)). Brain regions lacking these criteria were classified as low-sensitivity regions (dorsal raphe nucleus (DRN), paraventricular nucleus of the hypothalamus (PVN), basolateral amygdala (BLA), ventral tegmental area (VTA), olfactory bulb (OB) and the medial prefrontal cortex (mPFC)). Next, we explored the predicted upstream regulators of overlapping DEGs in high- versus low-sensitivity brain regions, which revealed greater enrichment for oestrogen, progesterone, testosterone, dopamine and other ligands with similar receptor affinity or structural homology in high-sensitivity regions (Fig. 1f).

We next performed weighted gene co-expression network analysis (WGCNA) across all 11 brain regions, resulting in 9 co-expression modules (Extended Data Fig. 1k–n). Module–trait correlations identified significant correlations between high-sensitivity regions in RE dams and the brown, green, yellow and magenta modules (hereafter termed RE-sensitive modules; Fig. 1g). Genes within RE-sensitive modules displayed significant enrichment for DEGs from dHF, NAc, mPOA, LC and vHF (Fig. 1h). Functional annotation analysis of the genes from these modules highlighted signalling pathways related to neuromodulators, such as oestrogen (yellow and brown) and dopamine (green and magenta), in agreement with our upstream regulator analyses (Fig. 1i and Extended Data Fig. 1o,p).

**Extended Data Fig. 2:**
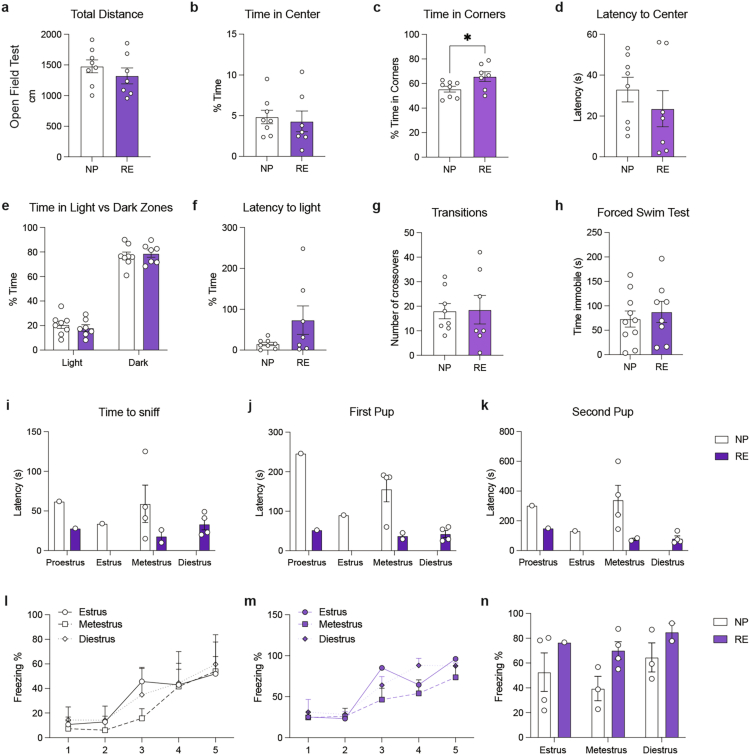
RE-associated behavioral adaptations are not influenced by locomotion, anxiety-/depressive-like behaviors, or estrous stage. **a-d**) In the open field test, there was no change in total distance, time spent in the center, or latency to the center. While there was a significant increase in time spent in corners for RE dams (unpaired two-sided Student’s t-test, *p = 0.0382), this did not impact any other outcomes on the open field test. **e-g**) In the light-dark box, there was no difference in time spent in light *vs*. dark zones, latency to enter the light zone, or transitions between zones. For panels a-g, RE (*n* = 7), NP (*n* = 8). **h**) There was no difference in time spent immobile on the forced swim test. RE (*n* = 8), NP (*n* = 10). **i-k**) Stratification of pup retrieval behaviors by estrous stage (Fisher’s exact test, *p* = 0.1002). RE (*n* = 7), NP (*n* = 6). **l-n**) Stratification of contextual fear conditioning by estrous stage (Fisher’s exact test, *p* = 0.6041). RE (*n* = 7), NP (*n* = 10). Error bars represent mean ± SEM. All *p*-values from Fisher’s exact tests comparing estrous stage distributions across groups for all behavioral experiments are provided in Supplementary Table 19. Source data

We next investigated whether such transcriptomic findings correspond to sustained functional alterations in RE females. We performed behavioural assessments about 16 weeks after pup weaning based on functions associated with brain regions displaying the most robust transcriptional changes, including maternal behaviour, learning and memory. To assess maternal behaviour, we tested pup retrieval in the home cage by placing two donor pups in opposite corners away from the nest. RE dams retrieved pups with significantly reduced latency compared with NP females (Fig. 1j), consistent with previous findings, although at a more extended timepoint in our studies^15^. Next, in a contextual fear conditioning paradigm, RE females exhibited both enhanced acquisition and context recall compared with NP (Fig. 1k,l), supporting previous reports of cognitive adaptations^7,10,12,13,16–22^. No significant differences were observed in the open field, light–dark box or forced swim tests (Extended Data Fig. 2a–h). Additionally, the distribution of oestrous stages was comparable between groups (Extended Data Fig. 2i–n).

## Contributions of pregnancy and postpartum events

**Extended Data Fig. 3:**
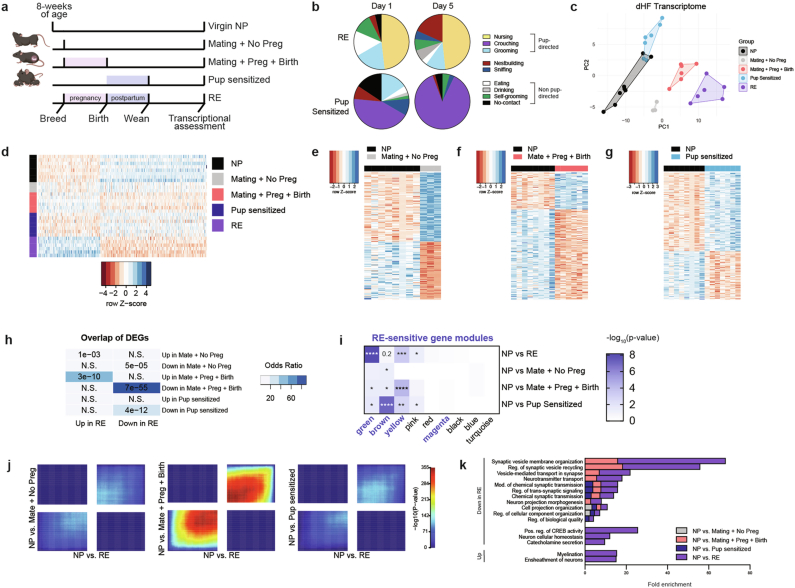
Postpartum experiences modulate the extent of RE-associated dHF transcriptomic and behavioral adaptations. **a**) Experimental design to examine the contribution of discrete female reproductive events. Created in BioRender; Lab, M. https://biorender.com/4tj0uq7 (2026). **b**) Chi square analyses showing significant shifts towards pup-directed behaviors after four days of pup exposure in Pup Sensitized virgin females (χ²(7) = 33.52, *p* = 2.11 × 10^−5^). **c**) Unsupervised principle components analysis of NP *vs*. RE DEGs (DESeq2, p_adj_ < 0.05) from dHF. **d**) Heatmap of differential expression profiles of the top 500 genes (ranked by ascending nominal *p*-values, DESeq2) between NP *vs*. RE. **e**) Heatmaps of DEGs for NP *vs*. Mating + No Pregnancy, **f**) NP *vs*. Mating + Pregnancy + Birth, **g**) NP *vs*. Pup sensitized dHF transcriptomes. **h**) Odds ratio analysis of DEG overlap for all comparisons (*vs*. NP). Insert numbers indicate respective *p* values for each association following Fisher’s exact test (N.S., *p* > 0.05). **i**) Overlap of DEGs (*vs*. NP, p_adj_ < 0.05) for each gene co-expression module (Fisher’s exact test, *p < 0.05, **p < 0.01, ***p < 0.001, ****p < 0.0001). High RE sensitivity modules are in bold. **j**) Threshold-free comparison of indicated comparisons by rank-rank hypergeometric overlap. The lower left and upper right quadrants represent concordant gene regulation. **k**) Shared ontologies following individual reproductive exposures *vs*. processes exclusive to the combined experiences in RE (FDR < 0.05).

We next sought to resolve the reproductive event(s) that contributed most substantially to the sustained transcriptional alterations that we observed. To do so, we compared females that were successfully bred to males but did not become pregnant (mating + no pregnancy), females that experienced pregnancy and parturition but did not transition to postpartum owing to pup removal on the day of birth (mating + pregnancy + birth), and virgin females exposed to 21 days of donor pup interactions (pup sensitized; Extended Data Fig. 3a,b). We focused our investigations on the dHF owing to its pronounced transcriptional sensitivity to RE. When comparing gene expression profiles in NP versus RE, we observed that the mating + pregnancy + birth group most closely resembled changes observed with RE, suggesting that pregnancy, and potentially its combination with birth, is a primary driver of dHF changes (Extended Data Fig. 3c,d). However, the magnitude of fold changes did not match that of the RE group, indicating that additional experiences are necessary.

Although visualization of differential expression profiles in the mating + no pregnancy and pup sensitized groups did not mimic changes seen with RE, significant DEGs across all groups (compared with NP) overlapped with those in RE (Extended Data Fig. 3e–h and Supplementary Table 3). Furthermore, DEGs from each comparison significantly overlapped with genes from RE-sensitive modules, with the strongest enrichment in mating + pregnancy + birth and pup sensitized groups (Extended Data Fig. 3i). Thus, despite distinct expression profiles between pup sensitized versus RE, pup interactions are likely to converge on similar regulatory networks, albeit through different DEGs that are critical for RE-driven transcriptional alterations. To further explore these relationships, we performed threshold-free rank–rank hypergeometric overlap (RRHO) analyses, which demonstrated transcriptional concordance across experiences, with the strongest overlap observed in the pregnancy-exposed group (Extended Data Fig. 3j). Furthermore, comparing gene ontology (GO) term analyses of DEGs for each group revealed significant enrichment in overlapping biological processes (Extended Data Fig. 3k). Notably, certain pathways were unique to the combination of reproductive exposures, including changes in CREB activity, catecholamine secretion and myelination.

**Extended Data Fig. 4:**
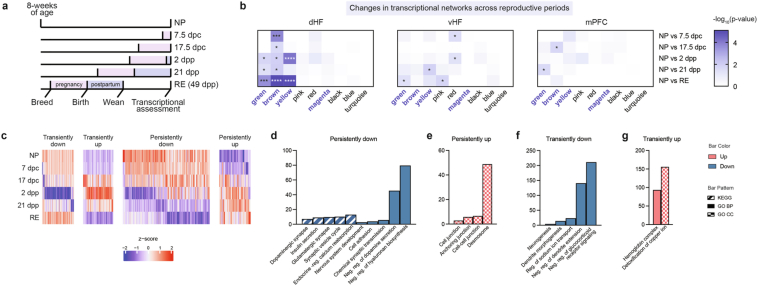
RE adaptations in the dHF transcriptome integrate pregnancy and postpartum experiences. **a**) Experimental design to examine the trajectory of RE dHF gene expression changes across pregnancy and postpartum periods (dpc: days post-conception; dpp: days postpartum). **b**) Enrichment of DEGs (*vs*. NP) with RE-sensitive gene co-expression modules across reproductive time points in high (left, dHF), moderate (middle, vHF), and low (right, mPFC) transcriptional sensitivity to RE status (Fisher’s exact test, *p < 0.05, **p < 0.01, ***p < 0.001, ****p < 0.0001). **c**) Time course analysis of dHF gene expression identifying patterns of transient *vs*. persistent up- and downregulation across pregnancy and postpartum (FDR < 0.05). **d-g**) Select GO terms and KEGG pathways enriched for dHF genes exhibiting (**d**) persistent downregulation, (**e**) persistent upregulation, (**f**) transient downregulation, and (**g**) transient upregulation across reproductive stages (FDR < 0.05).

We next conducted a controlled time-course study to examine the transcriptional dynamics across early and late gestational and postpartum stages in age-matched females (Extended Data Fig. 4a). Based on the hypothesis that RE-induced transcriptional alterations require multiple reproductive events to achieve full induction, we first compared DEGs at each timepoint (versus NP) to genes from RE-sensitive modules. DEGs from all comparisons significantly overlapped with the brown module, with postpartum exposures also enriching for the green and yellow modules (Extended Data Fig. 4b, left). To assess the regional specificity of these dynamic changes, we analysed bulk RNA-sequencing (RNA-seq) data from the vHF, a region that displayed moderate sensitivity to RE (Extended Data Fig. 4b, middle), along with the mPFC, which exhibited few DEGs (Extended Data Fig. 4b, right). These regions had limited overlap with RE-sensitive module genes, emphasizing the specificity of pathways in brain regions displaying heightened sensitivity to RE (Supplementary Tables 4–6).

To characterize cumulative transcriptional changes, we performed temporal analyses to detect genes displaying sustained or transient expression alterations. This revealed a prominent set of genes showing persistent and progressive downregulation, most pronounced during postpartum (Extended Data Fig. 4c), that enriched for processes related to glutamatergic synapses, dopaminergic signalling and endocrine-related pathways (Extended Data Fig. 4d). Conversely, genes with persistent upregulation enriched for pathways associated with cell junction dynamics (Extended Data Fig. 4e). Additionally, we identified gene sets displaying transient alterations, including downregulation of neurogenesis-related pathways and upregulation of copper ion metabolism (Extended Data Fig. 4f,g), consistent with other studies (reviewed in refs. ^23,24^). In total, these findings highlight postpartum as a key window for reinforcing RE-induced alterations.

## Chronic postpartum stress disrupts dHF adaptations

**Fig. 2:**
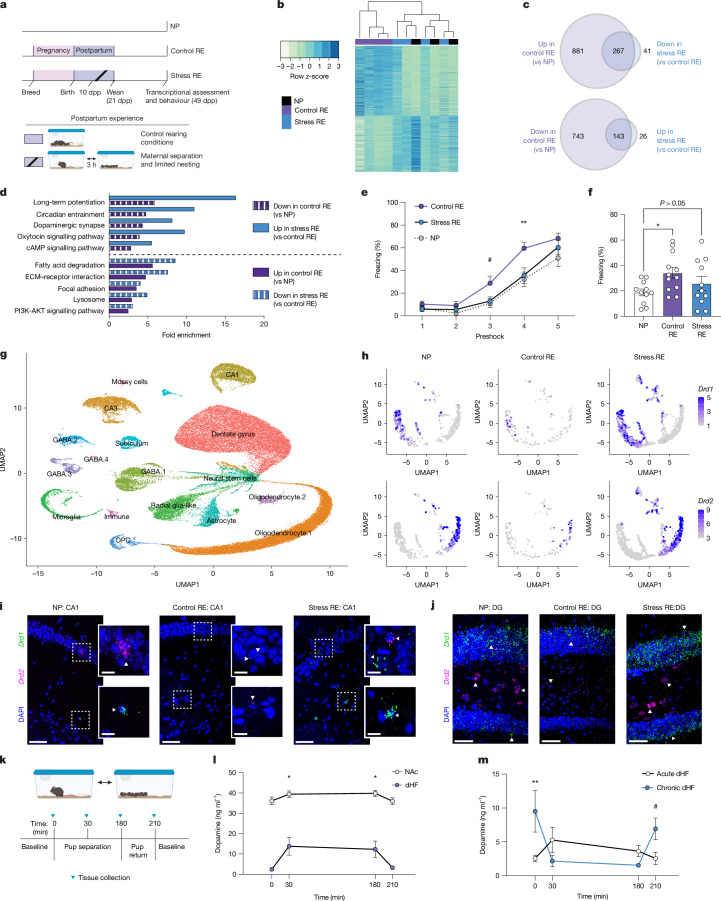
Postpartum stress disrupts long-term maternal dHF adaptations. **a**, Postpartum stress paradigm. Dams were provided with limited nesting and subjected to daily 3 h pup separations between 10–20 dpp. **b**, Heat map of significant DEGs (adjusted *P* < 0.05) with hierarchical clustering. **c**, Venn diagrams showing overlap of DEGs exhibiting opposing directionality for indicated comparisons. **d**, Select KEGG pathways for DEGs with opposing directionality (FDR < 0.05). ECM, extracellular matrix. **e**,**f**, Control RE, stress RE and NP during contextual fear acquisition (**e**; two-way rmANOVA, *P* = 0.017, Tukey’s multiple comparisons test; control RE versus NP, #*P* < 0.1, **P = 0.01) and context recall (**f**; one-way ANOVA, *P* = 0.05, Tukey’s multiple comparisons test; *P < 0.05). Control RE (*n* = 12), NP (*n* = 11) and stress RE (*n* = 11). **g**, Uniform manifold approximation and projection (UMAP) of cell clusters. **h**, Subclustering of the GABA.2 neuronal population showing reduced *Drd1* (top) and *Drd2* (bottom) expression in control RE. **i**,**j**, Representative images for *Drd1* and *Drd2* mRNAs in dorsal CA1 (**i**) and DG (**j**). Scale bars: 50 μm (main image), 12.5 μm (expanded region). **k**, Experimental design of pup separation test. **l**, Dopamine levels in dHF (*t*_0_ (*n* = 8); *t*_30_ (*n* = 9); *t*_180_ (*n* = 8); *t*_210_ (*n* = 5)) and NAc (*t*_0_ (*n* = 9); *t*_30_ (*n* = 9); *t*_180_ (*n* = 7); *t*_210_ (*n* = 5)) in response to acute pup separation (two-way ANOVA, *P* = 0.0089, Dunnett’s multiple comparisons test; *P < 0.05). **m**, Dopamine levels in response to acute (*t*_0_ (*n* = 9); *t*_30_ (*n* = 12); *t*_180_ (*n* = 12); *t*_210_ (*n* = 8)) and chronic (*t*_0_ (*n* = 5); *t*_30_ (*n* = 5); *t*_180_ (*n* = 5); *t*_210_ (*n* = 5)) pup separations (two-way ANOVA, interaction *P* = 0.0048, Tukey’s multiple comparisons test; #*P* < 0.1, **P < 0.01). Data are mean ± s.e.m. Panels **a** and **k** created in BioRender; Lab, M. https://biorender.com/mvh9p5o (2026). Source data

**Extended Data Fig. 5:**
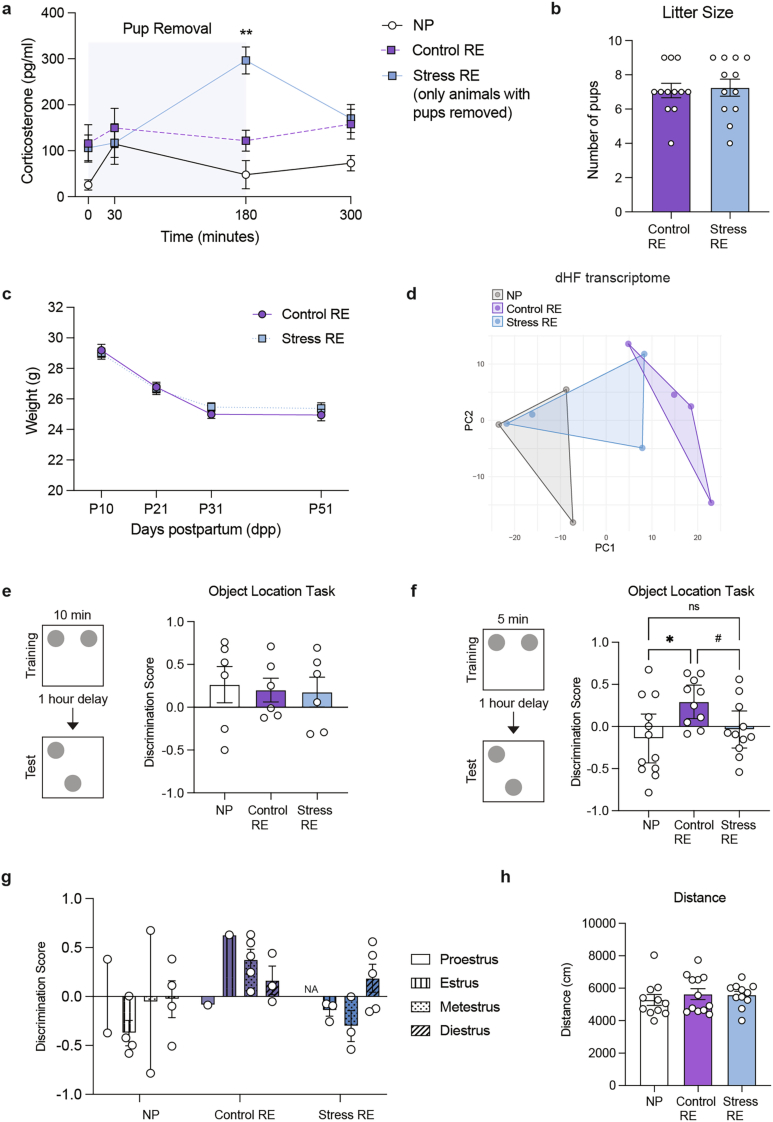
Postpartum stress attenuates spatial learning enhancements independent of metabolic, locomotor, or hormonal measures. **a**) Pup separation increases maternal corticosterone levels over 3-h (two-way rmANOVA, group: *p* = 0.0006, Tukey’s multiple comparisons test: Control *vs*. Stress RE, **p < 0.01). *n* = 6 animals/group. **b**) No effect of postpartum stress on litter size. *n* = 12/group. **c**) Postpartum stress did not alter maternal weights. **d**) Unsupervised principal components analysis of dHF transcriptomes. **e**) All groups discriminated the novel location following 10-min of training on the object location task (one way ANOVA, *p* = 0.9378) *n* = 6 animals/group. **f**) Control RE (*n* = 10), Stress RE (*n* = 11), and NP (*n* = 12) on the object location task following 5-min of training (one-way ANOVA, *p* = 0.0278, Holm-Šídák multiple comparisons test; *p < 0.05, #*p* < 0.1). **g**) Stratification of object location test score by estrous stage (Fisher’s exact test, *p* = 0.5551). Control RE (*n* = 10), Stress RE (*n* = 11), NP (*n* = 12). **h**) There was no difference in the total distance traveled in the open field test across groups. Control RE (*n* = 12), Stress RE (*n* = 11), NP (*n* = 11). Error bars represent mean ± SEM. Source data

We next examined whether postpartum perturbations may disrupt pathways that reinforce RE-induced alterations in dHF. We implemented a maternal stress paradigm that increases stress hormones and disrupts key postpartum experiences, including nursing and pup interactions, from 10–20 dpp (Fig. 2a and Extended Data Fig. 5a). During this period, dams were separated from their pups for 3 h daily and received limited nesting until pup weaning (stress RE). No differences in litter sizes or postpartum weights were observed (Extended Data Fig. 5b,c). Comparison of stress RE dHF transcriptional profiles revealed intermediate clustering between NP and control RE groups (Fig. 2b, Extended Data Fig. 5d and Supplementary Table 7), suggesting that postpartum stress disrupts the extent of RE alterations in dHF. Approximately 85% of control RE versus stress RE DEGs showed opposing directionality from NP versus control RE, enriching across pathways associated with long-term potentiation, dopaminergic synapses, oxytocin signalling and other processes (Fig. 2c,d).

Since our pathway analyses indicated changes in long-term potentiation, we focused our behavioural assessments on contextual fear conditioning. Control RE females exhibited enhanced acquisition and context recall compared to NP, whereas stress RE females showed no significant differences versus NP (Fig. 2e,f). To further evaluate dHF-dependent function, we used the object location task. Given prior findings that adult females require 10 min of training for reliable discrimination^25^, we first used this duration to confirm that all groups could successfully identify the moved object (Extended Data Fig. 5e). We then reduced the training time to 5 min. Under these conditions, control RE mice discriminated the moved object, whereas NP and stress RE females did not (Extended Data Fig. 5f), an effect that was not attributed to oestrous stage or locomotion (Extended Data Fig. 5g,h).

**Extended Data Fig. 6:**
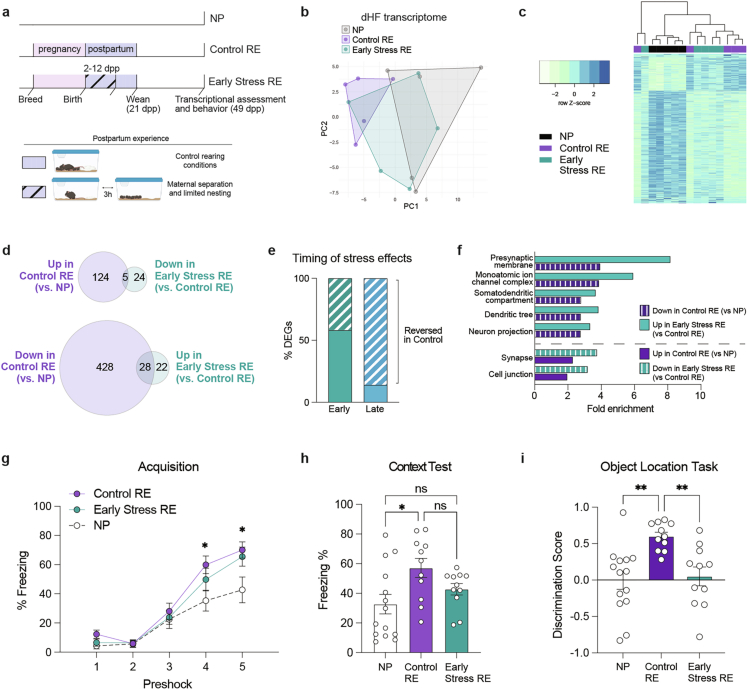
Early postpartum stress impact on maternal dHF plasticity. **a**) Early postpartum stress paradigm. Dams were provided with limited nesting and subjected to pup separation 3 h daily between 2–12 dpp. Created in BioRender; Lab, M. https://biorender.com/mvh9p5o (2026). **b**) Unsupervised principal components analysis of dHF transcriptomes. **c**) Heatmap of all significant DEGs (DESeq2, p_adj_ < 0.05) with hierarchical clustering. **d**) Venn diagrams showing overlap of DEGs altered in Control RE (*vs*. NP) and exhibiting opposing directionality in Early Stress RE (*vs*. Control RE). **e**) DEGs between Control RE *vs*. Early Stress RE (left) and Control RE *vs*. Late Stress RE (right), shown as percentages of total DEGs. Slashes denote the fraction reversed in Control RE relative to NP. **f**) RE-dependent enrichment in select Gene Ontology pathways (Cellular Component) are significantly reversed in Early Stress RE dHF (FDR < 0.05). **g**) Control RE, but not Early Stress RE, dams froze more than NP during the acquisition phase of contextual fear conditioning (two-way rmANOVA, interaction *p* = 0.0179, Tukey’s multiple comparisons test: Control RE *vs*. NP, *p < 0.05). **h**) During the context recall test, NP froze significantly less than Control RE (one-way ANOVA, *p* = 0.0219; Tukey’s multiple comparisons test, *p < 0.05). **i**) Control RE dams spent more time investigating the moved object in the object location task compared to NP and Early Stress RE dams after 5-min training and a 24-h delay before testing (one-way ANOVA, *p* = 0.001; Tukey’s multiple comparisons test: **p < 0.01) For panels g-i, Control RE (*n* = 11), Early Stress RE (*n* = 11), NP (*n* = 14). Error bars represent mean ± SEM. Source data

We next tested whether the same maternal stress paradigm from 2–12 dpp may also disrupt dHF adaptations (Extended Data Fig. 6a). Similar to the ‘late’ stress RE group, dHF transcriptome profiles from the early stress RE group clustered between those of NP and control RE groups (Extended Data Fig. 6b,c and Supplementary Table 8). DEGs with opposing directionality between NP versus control RE and control RE versus early stress RE had about 41% overlap (Extended Data Fig. 6d,e), and associated with synaptic plasticity pathways (Extended Data Fig. 6f). Thus, early and late postpartum stress similarly perturb dHF RE transcriptomic adaptations, with early stress exerting a less extensive effect. Consistent with this finding, there were no differences between early stress RE versus NP in contextual fear conditioning or the object location task (Extended Data Fig. 6g–i).

## Dopaminergic modulation underlying dHF plasticity

**Extended Data Fig. 7:**
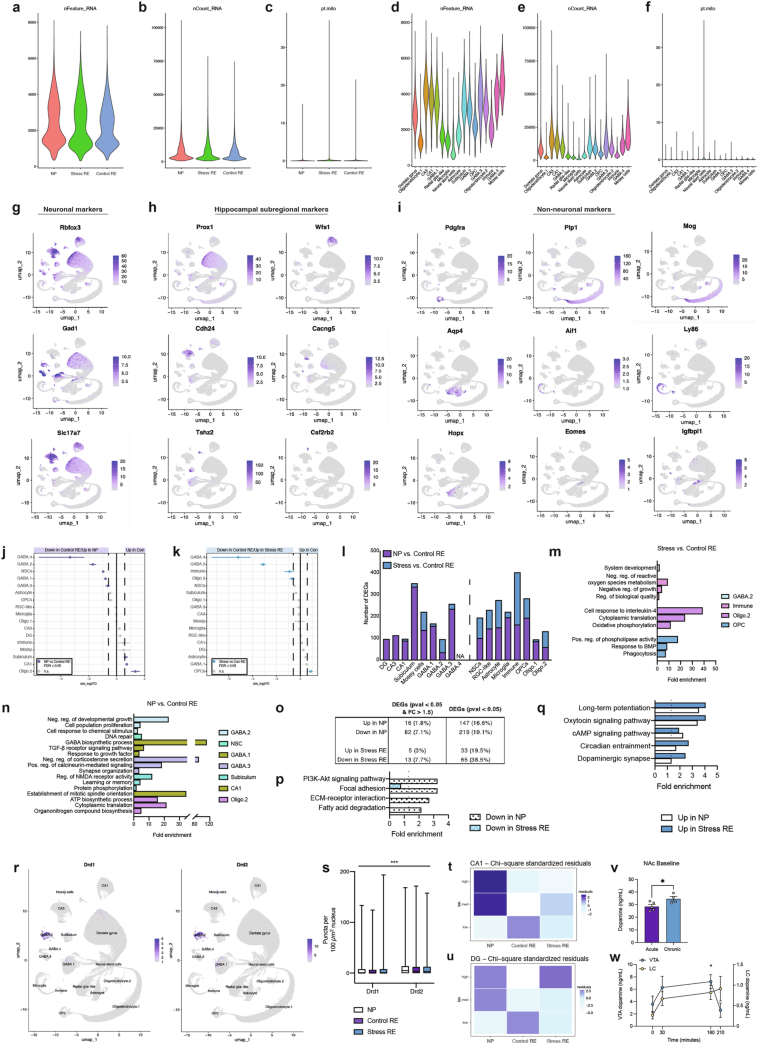
Validation of snRNA-seq analyses. Violin plots showing (**a**) number of detected genes, (**b**) UMI number, and (**c**) mitochondrial gene percentage, by group and **d-f**) annotated clusters. Unsupervised clustering of snRNA-seq data with cells colored for (**g**) neuronal, excitatory, inhibitory markers; (**h**) hippocampal subregional; and (**i**) non-neuronal markers. **j-k**) Point-range plots depicting proportions of cell-type clusters between (**j**) NP *vs*. Control RE, and (**k**) Stress RE *vs*. Control RE (scProportionTest, FDR < 0.05 and |log_2_Differerence| > log_2_(1.5)). *n* = 6 NP, 4 Control RE, 5 Stress RE. **l**) Number of DEGs following pseudobulk analysis (*p* < 0.05 and FC > 1.5). **m-n**) Ontology analyses for (**m**) Stress *vs*. Control RE and (**n**) NP *vs*. Control RE pseudobulk DEGs (FDR < 0.05). **o**) Overlap of bulk and pseudobulk DEGs by directionality. **p-q**) KEGG pathways enriched from overlapping DEGs from (o, *p* < 0.05). **r**) UMAPs of *Drd1* and *Drd2* expression. **s**) Quantification of Drd1 and Drd2 puncta in CA1 and DG (three-way LMM; group x region x probe ***p = 2.466 × 10^−9^). Box plots show median (center line), interquartile range (box), and minimum-to-maximum values (whiskers). **t-u**) Chi-square tests of *Drd1* and *Drd2* puncta distributions across expression bins for (**t**) CA1 (χ²(4) = 30.69, *p* = 3.5 × 10^−6^) and (**u**) DG (χ²(4) = 63.12, *p* = 6.4 × 10^−13^). **v**) Basal dopamine in NAc (unpaired two-sided Student’s t-test, *p = 0.0371). Acute (*n* = 5), Chronic (*n* = 4). **w**) Dopamine levels in VTA (*n*: t_0_ = 6; t_30_ = 8; t_180_ = 7; t_210_ = 6) and LC (*n*: t_0_ = 5; t_30_ = 8; t_180_ = 8; t_210_ = 6) during acute pup separation (two-way ANOVA followed by Dunnett’s multiple comparisons test: VTA t_0_ *vs*. VTA t_180_, *p = 0.0476). Error bars represent mean ± SEM. Source data

To identify the mechanisms underlying RE-induced dHF plasticity, we next leveraged the transcriptomic and behavioural signatures shared between NP and stress RE groups, focusing all subsequent analyses on the late postpartum stress paradigm given stronger disruption to RE adaptations. To identify the dHF cell-types that integrate hormone and neurotransmitter signalling to drive RE-related plasticity, we next performed single-nuclei RNA sequencing (snRNA-seq). Following rigorous quality control assessments to remove confounding sources of variation, such as mitochondrial mapping percentage, doublets, and cell-type contamination (Extended Data Fig. 7a–f), we retained 109,334 nuclei for downstream analysis (NP (*n* = 37,070); control RE (*n* = 35,631); stress RE (*n* = 36,633)). Cell-type annotation resulted in 16 distinct clusters (Fig. 2g and Extended Data Fig. 7g–i), including excitatory neuron subtypes (dentate gyrus (DG), CA1, CA3, subiculum and mossy cells; 43.3% of total), γ-aminobutyric acid-producing (GABAergic) neurons (GABA.1–GABA.4; 9.1% of total), neural progenitors (neural stem cells and radial glia-like cells; 9.7% of total) and non-neuronal or glial populations (astrocytes, oligodendrocytes, OPCs, microglia and immune cells; 37.9% of total).

Since our cell-type deconvolution analyses indicated downregulation of neuronal markers, we first confirmed shifts in proportional differences across major cell types, with GABA.2 and GABA.4 clusters reduced in control RE dHF compared to both NP and stress RE (Extended Data Fig. 7j,k). Following pseudobulk aggregation, we identified DEGs across both neuronal and non-neuronal subtypes, with excitatory neuron alterations predominantly observed between NP versus control RE, particularly within the subiculum (Extended Data Fig. 7l). By contrast, the most pronounced neuronal changes between control and stress RE were observed in the GABA.2 and mossy cell populations (Extended Data Fig. 7l). GO term analysis of DEGs from cell-types displaying proportional differences enriched for pathways associated with responses to endogenous stimuli, cell proliferation, synaptic function and metabolic processes (Extended Data Fig. 7m,n). Notably, DEGs shared between single-cell and bulk analyses enriched for similar pathways, indicating cross-platform convergence (Extended Data Fig. 7o–q).

We next focused on the GABA.2 cluster owing to shared proportional and transcriptional changes observed in comparison to both NP and stress RE groups, which suggested a common mechanism limiting dHF plasticity. Marker gene analysis revealed significant enrichment of dopamine receptor D_1_ (*Drd1*) and D_2_ (*Drd2*) mRNA expression (Extended Data Fig. 7r), aligning with our prior bioinformatic analyses implicating dopaminergic regulation. To further resolve this population, we subclustered the GABA.2 cells and identified distinct subpopulations expressing *Drd1* and *Drd2*, both of which appeared to be altered in control RE mice (Fig. 2h). In a separate cohort, we validated these changes using RNA fluorescence in situ hybridization (FISH) in dorsal CA1 (Fig. 2i and Extended Data Fig. 7s,t), where dopamine receptor-expressing interneurons have been described^26,27^. We also examined whether such changes extend to other dopamine-sensitive neuronal populations. RNA FISH in the DG, which contains excitatory *Drd1* and *Drd2*-expressing neurons (in granule and hilar mossy cells, respectively), similarly revealed significant changes in the distribution of *Drd1* and *Drd2* puncta across groups, alongside expected regional variations consistent with the sparser distribution of dopamine receptor-expressing neurons in CA1^28^ (Fig. 2j and Extended Data Fig. 7u).

Since these findings established dopaminergic signalling as a potential mediator of persistent RE-induced adaptations, this prompted us to examine whether maternal stress disrupts this process within its defined postpartum window. Given prior evidence that pup separation elevates dopamine levels in NAc^29,30^, we first investigated whether a similar response occurs in dHF (Fig. 2k). Dopamine levels increased during pup separation, with expected regional differences in NAc versus dHF reflecting the strength of innervation to these regions (Fig. 2l). Since acute and chronic stress produce different biological responses, we next assessed the effect of repeated separation stress, revealing elevated baseline dopamine in both dHF and NAc tissues (Fig. 2m and Extended Data Fig. 7v). Thus, decreased *Drd1/2*-expressing neurons may reflect a compensatory response to lower basal dopamine, consistent with our findings of reduced dopamine-related transcriptional activity in control RE.

## Parity alters H3 dopaminylation in mice and humans

**Extended Data Fig. 8:**
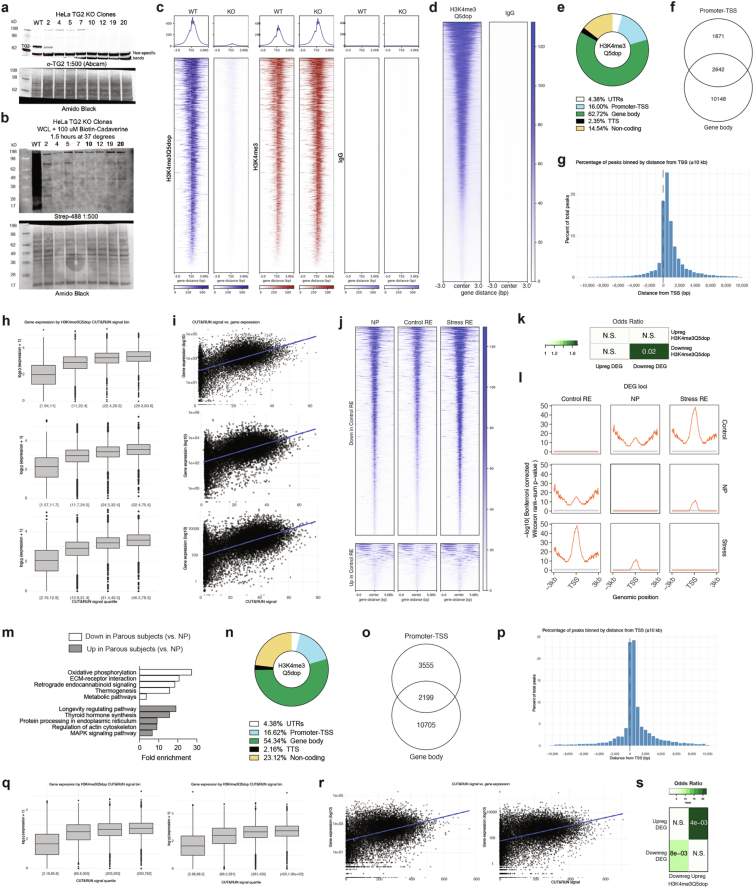
H3K4me3Q5dop corresponds with transcription. **a-b**) Validation of TG2 knockout cells using (**a**) western blot, and (**b**) transamidation activity assay. **c**) Heatmaps showing H3K4me3(Q5dop) enrichment in TG2 KO *vs*. WT. **d**) Heatmaps of H3K4me3Q5dop peaks (*vs*. IgG) at all enriched loci in NP dHF. **e, n**) Distribution of H3K4me3Q5dop across genic loci. **f, o**) Overlap of H3K4me3Q5dop at promoter-TSS and gene body regions. **g, p**) H3K4me3Q5dop enrichment within 2kB of TSS. **h, q**) Boxplots of log_10_-transformed gene expression across quartiles of H3K4me3Q5dop signal for (**h**) NP (top, *n* = 4), Control RE (middle, *n* = 4), Stress RE (bottom, *n* = 4), and (**q**) NP (left, *n* = 3), parous subjects (right, *n* = 5). Differences were assessed within each group (Kruskal-Wallis test, *p* < 2.2 × 10^−16^). Boxes indicate interquartile range with median center line, and whiskers extend to 1.5x IQR. **i, r**) Two-sided Spearman’s rank correlations between H3K4me3Q5dop enrichment *vs*. mean gene expression (log_10_-transformed) for (**i**) NP (top; ρ = 0.55), Control RE (middle; ρ = 0.52), Stress RE (bottom; ρ = 0.56) and (**r**) NP (left; ρ = 0.41), Parous (right; ρ = 0.40). For all, *p* < 2.2 × 10^−16^. **j**) Heatmaps of differential peaks (Diffbind, *p* < 0.05; log_2_FoldChange ≥ |0.1|), by directionality. **k, s**) Odds ratio analysis of differential H3K4me3Q5dop peaks and DEGs (Fisher’s exact test; inset *p*-values; N.S. *p* > 0.05). **l**) Statistical comparison of H3K4me3Q5dop profiles across DEG-associated loci showing –log_10_ Bonferroni-corrected *p*-values from bin-wise two-sided Wilcoxon rank-sum tests. Gray bars indicate non-significant bins after multiple-testing correction. Orange lines indicate statistical significance of signal differences across the TSS window, with higher values indicating greater confidence in differential enrichment between groups. Mouse tissues are shown in d-l and human tissues in m-s. For gel source data, see Supplementary Fig. 1.

Together, changes in dopamine modulation and gene expression point to a role for dopamine-dependent epigenetic mechanisms in shaping persistent transcriptional states. Motivated by this idea, we turned to a recently characterized class of histone post-translational modifications that is dependent on intracellular pools of biogenic monoamines, including serotonin, dopamine and histamine, termed monoaminylations^31–33^. This process involves transamidation of monoamines onto the glutamine 5 residue of histone H3 (H3Q5), a site adjacent to the canonically permissive lysine 4 tri-methylation (H3K4me3), by tissue transglutaminase 2^34^ (TG2; Extended Data Fig. 8a–c). Monoaminylation at H3Q5 can coexist with H3K4me3 and is responsive to environmental stimuli such as stress or drugs of abuse, with downstream effects on transcriptional output^31–33,35–40^. Using our previously validated H3K4me3Q5dop antibody^32^, we performed CUT&RUN-seq to test the involvement of H3 dopaminylation in RE transcriptional adaptations. The majority of H3K4me3Q5dop peaks annotated to genic loci, with a large proportion occurring within 2 kb of transcriptional start sites (TSSs; Extended Data Fig. 8d–g). Across all groups, higher H3K4me3Q5dop enrichment associated with increased gene expression (Extended Data Fig. 8h,i).

**Fig. 3:**
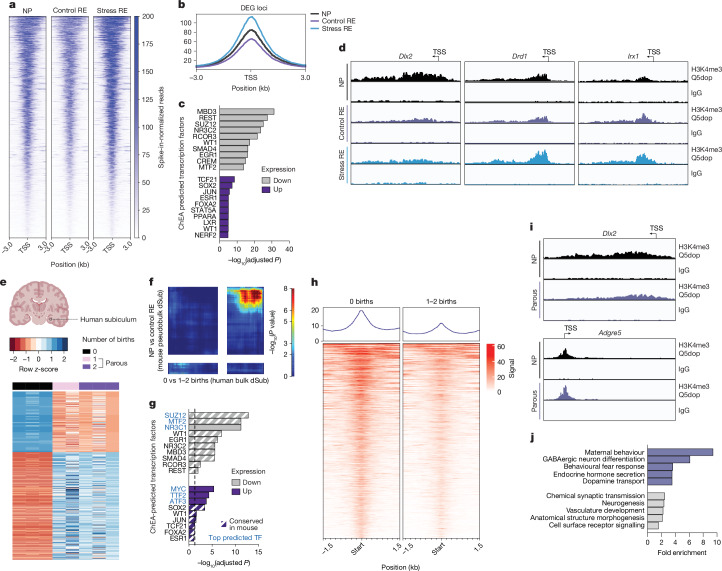
Parity downregulates H3 dopaminylation enrichment in dHF of humans and mice. **a**,**b**, Heat maps (**a**) and profiles (**b**) for H3K4me3Q5dop enrichment at TSSs for NP versus control RE DEGs. Plots represent merged data from *n* = 4 mice per group. **c**, ChEA ontology analysis of transcription factor enrichment (Benjamini–Hochberg, adjusted *P* < 0.05) for DEGs in NP versus control RE mice. **d**, Representative genome browser tracks for differentially enriched H3K4me3Q5dop peaks (versus IgG). Each track represents merged signal for *n* = 4 samples per group. **e**, Heat map of differential expression profiles of the top 500 genes, ranked by ascending *P* values, from bulk RNA-seq of human dSub tissue, from NP individuals with 0 births (*n* = 3) versus parous individuals with 1–2 births (*n* = 5). Created in BioRender; Lab, M. https://biorender.com/mvh9p5o (2026). **f**, Threshold-free comparison of human dSub RNA-seq and mouse dSub pseudobulk from snRNA-seq using RRHO. The lower left and upper right quadrants represent concordant gene regulation. **g**, ChEA ontology analysis from human dSub DEGs (Benjamini–Hochberg, adjusted *P* < 0.05). Transcription factors (TFs) that are conserved between human and mouse analyses are indicated by white stripes. The dotted line indicates a threshold of 1.3. **h**, Heat maps and profiles of H3K4me3Q5dop enrichment in human dSub, comparing individuals with 0 versus 1–2 births at differential loci (*P* < 0.05, log_2_(fold change) ≥ |0.1|). **i**, Representative genome browser tracks for differentially enriched H3K4me3Q5dop peaks (versus IgG) from human dSub. Each track represents merged signal for NP (*n* = 3) and parous (*n* = 5) individuals. **j**, Top significantly enriched pathways from GO analysis of differential H3K4me3Q5dop loci (grey bars; ranked by FDR). Additional significant pathways are shown in purple.

We next performed differential peak analysis and observed that the majority of group-specific changes in H3 dopaminylation were downregulated in control RE dHF compared with both NP and stress RE (Extended Data Fig. 8j and Supplementary Tables 9 and 10). Integrating these peaks with DEGs from the same comparisons revealed significant overlap between downregulated peaks and genes decreased in control RE (Extended Data Fig. 8k). H3K4me3Q5dop enrichment at the TSS of RE-regulated DEGs was also reduced at most loci compared to NP and stress RE (Fig. 3a,b and Extended Data Fig. 8l). Stratifying DEGs by directionality revealed enrichment of canonical repressors among downregulated genes and ligand-responsive nuclear receptors among upregulated genes, pointing to ligand-responsive transcriptional regulators that may engage additional chromatin remodelling pathways (Fig. 3c,d).

To assess the translational relevance of these findings, we next performed transcriptomic and H3K4me3Q5dop profiling in human dorsal subiculum (dSub), a subregion of the dHF that serves as its primary output and was identified in our snRNA-seq analysis as the most transcriptionally responsive subregion to RE. Comparing age-matched NP individuals with parous individuals who had 1–2 previous births, we identified robust differential gene expression indicative of long-lasting effects of parity in human brain (Fig. 3e and Supplementary Tables 11 and 12). These transcriptional signatures showed strong concordance with mouse pseudobulk expression profiles (Fig. 3f), and were enriched for pathways related to extracellular matrix remodelling and metabolism (Extended Data Fig. 8m). Furthermore, both upregulated and downregulated DEGs in human dSub were enriched for upstream regulators similarly identified in mouse (Fig. 3g). We also performed H3K4me3Q5dop CUT&RUN-seq (Extended Data Fig. 8n–r) in human brain, where differential binding analysis showed widespread reduction in dopaminylation signal in parous dHF, corresponding with DEG analyses (Fig. 3h,i, Extended Data Fig. 8s and Supplementary Table 13). Pathway analysis of differential peaks revealed significant enrichment for loci involved in maternal behaviour, behavioural fear responses, hormone regulation and dopamine transport (Fig. 3j), suggesting conserved, RE-associated remodelling of dopamine-sensitive transcriptional programs in dHF.

## Dopamine suppression recapitulates RE outcomes

**Fig. 4:**
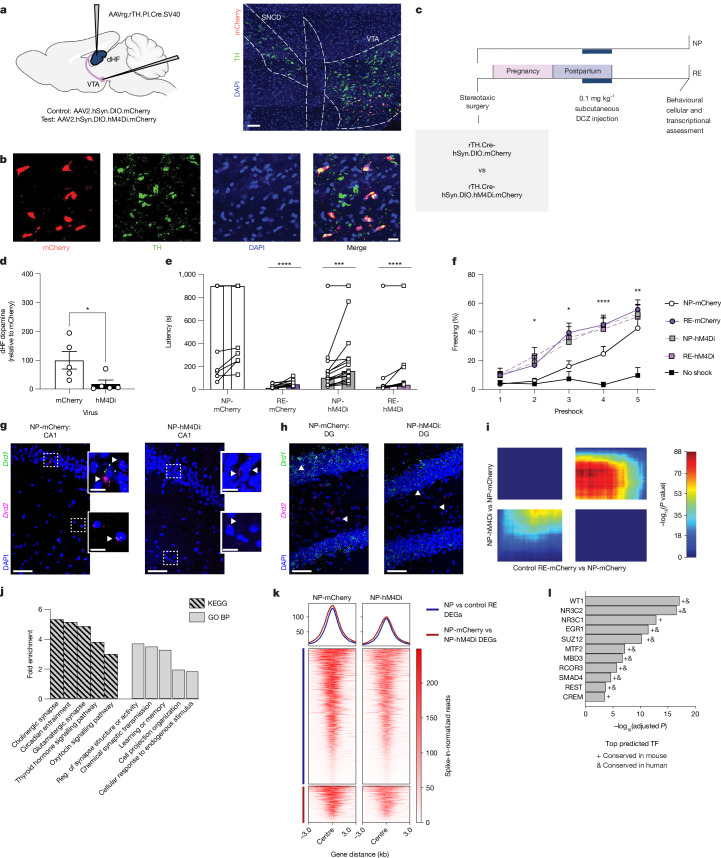
Chronic dopamine suppression is sufficient to mimic persistent maternal dHF plasticity. **a**, Schematic (left) and representative image (right) of DREADD targeting to VTA projection neurons following dHF injection of TH-driven retrograde Cre. SNCD, substantia nigra, compact part, dorsal tier. Scale bar, 100 μm. Created in BioRender; Lab, M. https://biorender.com/mvh9p5o (2026). **b**, Representative images showing overlap of virally induced mCherry with TH^+^ cells in VTA. Scale bar, 20 μm. Viral validation imaging in **a**,**b** was repeated in three independent experiments. **c**, Timeline of stereotaxic surgeries and DCZ injections from 10–20 dpp. **d**, dHF dopamine levels following inhibition of VTA projection neurons. Two-sided unpaired Student’s *t*-test; *P* = 0.0383; *n* = 5 per group. **e**, Pup retrieval task for RE-mCherry (*n* = 12), NP-hM4Di (*n* = 15) and RE-hM4Di (*n* = 11) versus NP-mCherry (*n* = 13) groups. Two-way rmANOVA, *P* < 0.0001, Sidak’s multiple comparisons test. **f**, Contextual fear acquisition for NP-mCherry (*n* = 11), RE-mCherry (*n* = 10), NP-hM4Di (*n* = 12), RE-hM4Di (*n* = 9) versus no shock (*n* = 5) groups. Two-way rmANOVA, *P* = 0.0031; Tukey’s multiple comparisons test. **g**,**h**, Representative images for *Drd1* and *Drd2* mRNAs in dorsal CA1 (**g**) and DG (**h**). Scale bars: 50 μm (main image), 12.5 μm (expanded region). **i**, Threshold-free comparison using RRHO, showing concordant gene regulation (bottom left and upper right quadrants) between indicated comparisons. **j**, Select GO and KEGG pathways enriched for NP-mCherry versus NP-hM4Di DEGs (FDR < 0.05). **k**, Heat maps and profiles of H3K4me3Q5dop enrichment at TSSs for DEGs in NP versus control RE (top, blue) and NP-mCherry versus NP-hM4Di (bottom, red). Plots represent merged data from *n* = 4 mice per group. **l**, Transcription factors identified from ChEA ontology analysis of downregulated DEGs (Benjamini–Hochberg, adjusted *P* < 0.05). Data are mean ± s.e.m. Source data

**Extended Data Fig. 9:**
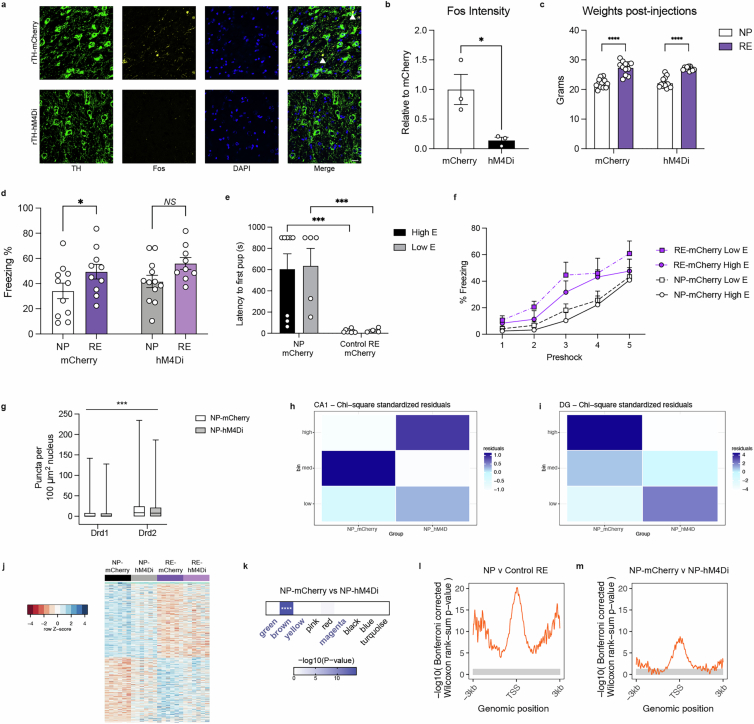
Assessment of VTA-dHF projection inhibition. **a**) Representative images (scale bar, 20 μm) and **b**) quantification of c-Fos in VTA TH+ neurons (unpaired two-sided Student’s t-test; *p = 0.03). *n* = 3 animals/group. **c**) NP-mCherry (*n* = 14), NP-hM4Di (*n* = 15), RE-mCherry (*n* = 12), and RE-hM4Di (*n* = 11) weights post-DCZ injections (two-way ANOVA; Tukey’s multiple comparisons corrections; ****p < 0.0001). **d**) NP-mCherry (*n* = 11), NP-hM4Di (*n* = 12), RE-mCherry (*n* = 10), and RE-hM4Di (*n* = 9) during context recall (two-way ANOVA*, p* = 0.0109; Fisher’s LSD, *p = 0.05). **e-f**) NP-mCherry and RE-mCherry binned by high *vs*. low estrogen (E) during (**e**) pup retrieval (two-way ANOVA, group: *p* < 0.0001, E: *p* = 0.8699); Tukey’s multiple comparisons test, ***p = 0.0001; RE-mCherry Low-E (*n* = 4), RE-mCherry High-E (*n* = 8), NP-mCherry Low-E (*n* = 5), NP-mCherry High-E (*n* = 8)), and (**f**) contextual fear acquisition (three-way rmANOVA, group: *p* = 0.0208, preshock: *p* < 0.0001, E: *p* = 0.3199; RE-mCherry Low-E (*n* = 3), RE-mCherry High-E (*n* = 7), NP-mCherry Low-E (*n* = 4), NP-mCherry High-E (*n* = 6)). Error bars represent mean ± SEM. **g**) *Drd1* and *Drd2* puncta quantification (three-way LMM; group x region: ***p = 2.559 × 10^−^^9^). Box plots show median (center line), interquartile range (box), minimum-to-maximum values (whiskers)*. n* = 3 animals/group. **h-i**) Chi-square tests of *Drd1* and *Drd2* puncta distributions across expression bins for (**h**) CA1 (χ^2^(2) = 4.33, *p* = 0.1145) and (**i**) DG (χ²(2) = 48.67, *p* = 2.7 × 10^−11^). **j**) Heatmap of top 500 genes (ranked by NP-mCherry *vs*. RE-mCherry ascending *p*-values). **k**) DEG overlap with RE-sensitive modules (****p < 0.0001). **l-m**) H3K4me3Q5dop profiles across DEG-associated loci, showing –log_10_ Bonferroni-corrected *p*-values from bin-wise two-sided Wilcoxon rank-sum tests across the TSS window. Source data

We next hypothesized that persistent reductions in dopamine tone may engage adaptive chromatin mechanisms to support enduring transcriptional and behavioural plasticity. As the VTA showed similar response patterns to pup separation (Extended Data Fig. 7w), we chemogenetically suppressed the dopaminergic VTA projection to dHF by bilaterally expressing a floxed inhibitory neuronal hM4Di-DREADD fused to mCherry (versus floxed mCherry controls) in the VTA, coupled with retrograde adeno-associated viral vectors (AAVs) expressing a tyrosine hydroxylase (TH)-dependent Cre recombinase into the dHF (Fig. 4a). We validated this approach by confirming selective mCherry expression in TH^+^ neurons of the VTA, as well as reduced FOS expression following administration of the selective DREADD agonist deschloroclozapine (DCZ; Fig. 4b and Extended Data Fig. 9a,b). To minimize injection-related stress, we restricted the window of chronic DCZ administrations. Since maternal stress from 10–20 dpp was sufficient to disrupt RE-induced plasticity, we posited that dHF dopamine suppression during this period may be sufficient to phenocopy RE-induced adaptations. Therefore, we administered DCZ to virgin NP females versus postpartum dams in parallel (NP-mCherry, NP-hM4Di, RE-mCherry and RE-hM4Di; Fig. 4c).

After confirming that our approach suppressed dHF dopamine (Fig. 4d) and did not induce gross metabolic changes (Extended Data Fig. 9c), we assessed behavioural outcomes one month after the final DCZ injection. In the pup retrieval test, failure to retrieve either pup occurred in 8 out of 13 NP-mCherry females, a result that occurred in only 1 out of 15 NP-hM4Di, 0 out of 12 RE-mCherry, and 1 out of 11 RE-hM4Di females (Fig. 4e). In contextual fear conditioning, NP-hM4Di females demonstrated enhanced acquisition, with no significant differences in the context recall test (Fig. 4f and Extended Data Fig. 9d). Oestrous stage analyses confirmed that the observed behavioural outcomes were driven by RE, independent of hormonal state or viral expression (Extended Data Fig. 9e,f).

Next, we examined whether dopamine suppression influences cellular receptor expression in the dHF. Similar to control RE, NP-hM4Di females had greater distribution of cells with low expression of *Drd1* and *Drd2*, compared with NP-mCherry (Fig. 4g,h and Extended Data Fig. 9g–i). Following transcriptional profiling to assess the degree to which dopamine suppression mirrors RE-associated gene expression patterns, we observed that NP-hM4Di exhibited an intermediate expression pattern relative to NP-mCherry versus RE-mCherry expression profiles (Extended Data Fig. 9j and Supplementary Table 14). RRHO analysis further supported transcriptional concordance between RE-mCherry and NP-hM4Di groups compared with NP-mCherry (Fig. 4i). Of note, NP-mCherry versus NP-hM4Di DEGs overlapped with genes from the RE-sensitive brown module (Extended Data Fig. 9k). Functional annotation of these DEGs revealed enrichment in pathways governing synaptic plasticity and responses to endogenous stimuli (Fig. 4j), similarly mirroring patterns altered in control RE dHF.

Finally, to assess whether the effects of dopamine suppression on dHF transcription are modulated in part through H3 dopaminylation, we conducted H3K4me3Q5dop CUT&RUN-seq in virally infected NP tissues. Examining H3K4me3Q5dop enrichment at the TSS of both RE-regulated DEGs and genes altered between NP-mCherry and NP-hM4Di groups, we observed significantly reduced signal at all loci examined in NP-hM4Di tissues (Fig. 4k, Extended Data Fig. 9l,m and Supplementary Table 15). We identified significant transcription factors regulating DEGs between NP-mCherry and NP-hM4Di, overlapping with those observed in parous individuals in both human dSub and our mouse model (Fig. 4l).

## Reversing H3 dopaminylation rescues stress effects

**Fig. 5:**
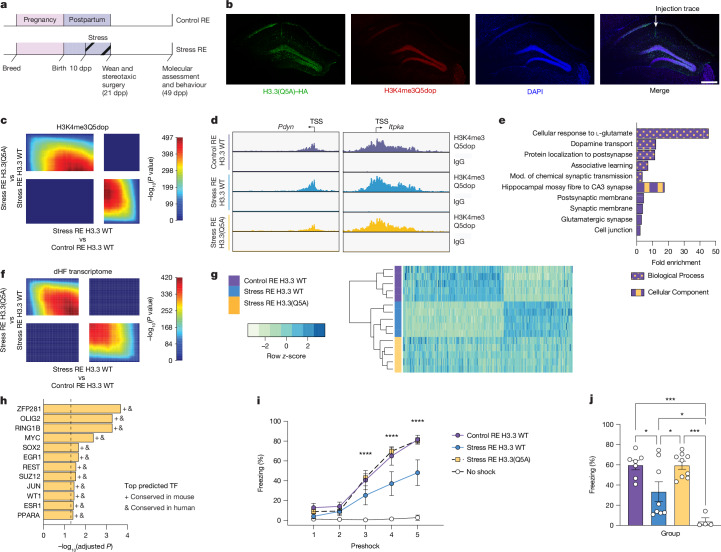
Reversal of H3 dopaminylation rescues dHF plasticity following postpartum stress. **a**, Experimental design depicting post-weaning viral infusion of AAV2-H3.3(Q5A) (to suppress H3Q5 dopaminylation) or AAV2-H3.3 WT (control) in control RE or stress RE. **b**, Representative image of viral targeting to dHF. The experiment was repeated three times. Scale bar, 400 μm. **c**, Threshold-free comparison using RRHO, showing reversal of differential H3Q5 dopaminylation peaks (upper left and bottom right quadrants) between indicated comparisons. **d**, Representative genome browser tracks for differentially enriched H3K4me3Q5dop peaks (versus IgG). Each track represents merged signal for *n* = 5 samples per group. **e**, Selected GO terms enriched for differential H3K4me3Q5dop loci altered in stress RE H3.3 WT (versus control RE H3.3 WT) and reversed in stress RE H3.3(Q5A) (FDR < 0.05). **f**, Threshold-free comparison using RRHO, showing discordant gene expression profiles between the indicated comparisons. **g**, Heat map of DEGs (DESeq2, adjusted *P* < 0.05), with hierarchical clustering. **h**, ChEA ontology analysis of stress RE H3.3 WT versus stress RE H3.3(Q5A) DEGs (Benjamini–Hochberg, adjusted *P* < 0.05). **i**, During fear acquisition, control RE H3.3 WT and stress RE H3.3(Q5A) dams showed increased freezing relative to no shock from shock 3 onwards (two-way rmANOVA, *P* = 0.0004; Tukey’s multiple comparisons test; ****P < 0.0001), whereas stress RE H3.3 WT dams did not exhibit increased freezing until shock 5 (Tukey’s multiple comparisons test, *P* = 0.0391). **j**, During context recall, control RE H3.3 WT and stress RE H3.3(Q5A) froze more than stress RE H3.3 WT and no shock (one-way ANOVA, *P* < 0.0001; Tukey’s multiple comparisons test; *P < 0.05, ***P < 0.001). **i**,**j**, Control RE H3.3 WT (*n* = 7), stress RE H3.3 WT (*n* = 8), stress RE H3.3(Q5A) (*n* = 9) and no shock (*n* = 4). Data are mean ± s.e.m. Source data

Finally, to determine whether H3 dopaminylation causally regulates RE adaptations, we manipulated H3 dopaminylation in dHF by transducing AAVs expressing histone H3.3 constructs—which incorporate into chromatin in post-mitotic neurons—encoding either the wild-type glutamine at position 5 (H3.3 WT, control) or a glutamine-to-alanine substitution (H3.3(Q5A)), which functions as a dominant negative mutant to reduce monoaminylated H3Q5^31–33,35,39^. We targeted H3 dopaminylation in stress RE dHF, as this group showed higher H3 dopaminylation levels versus control RE, and a more pronounced increase versus NP. Accordingly, stress RE dams received either H3.3 WT or H3.3(Q5A) AAVs, and were compared to control RE dams receiving H3.3 WT as a control (Fig. 5a,b).

**Extended Data Fig. 10:**
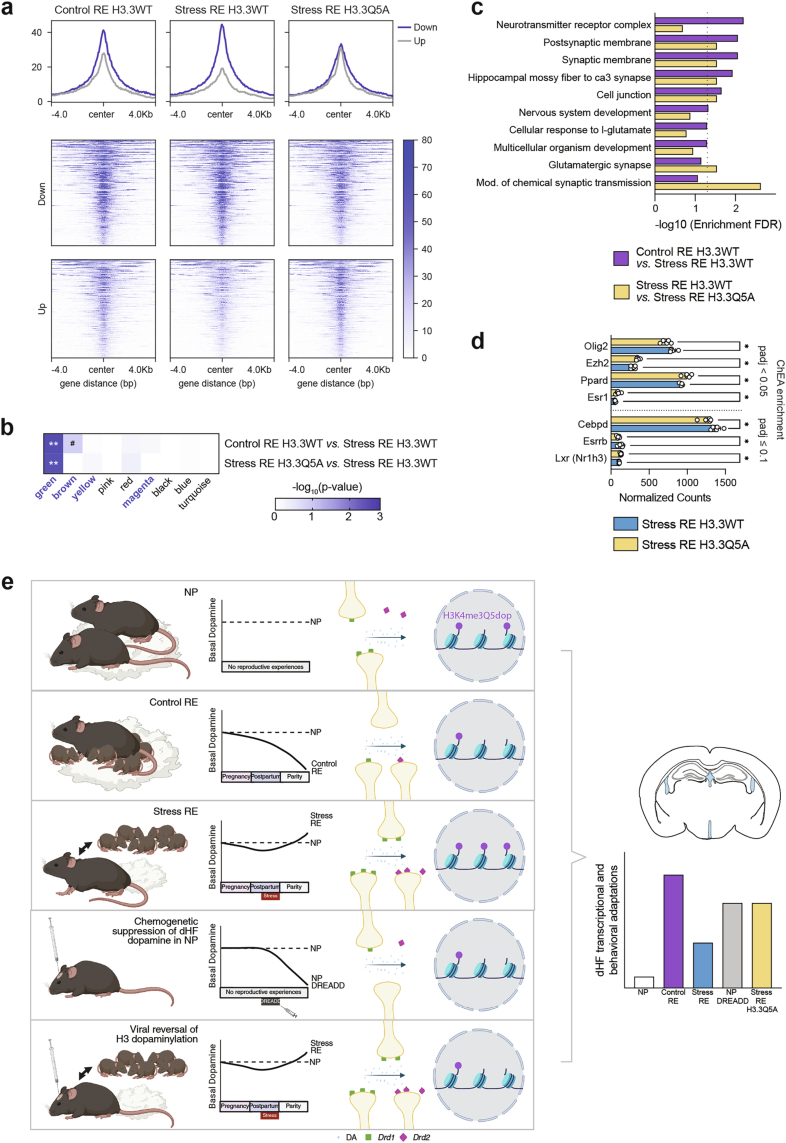
Effect of viral H3.3Q5A transduction in mouse dHF. **a**) Heatmaps of all differential peaks (Diffbind, *p* < 0.05; log_2_FoldChange ≥ |0.1| ) between Stress RE H3.3WT *vs*. Stress RE H3.3Q5A, separated by directionality. **b**) Overlap of DEGs with gene co-expression modules (Fisher’s exact test; **p < 0.01, #*p* < 0.1). High sensitivity RE modules are indicated in bold. **c**) GO term enrichment for overlapping differential H3 dopaminylation peaks and genes in Control RE H3.3WT *vs*. Stress RE H3.3WT (purple) and Stress RE H3.3WT *vs*. Stress RE H3.3Q5A (yellow) comparisons (FDR < 0.05). **d**) Normalized counts of select transcription factors and chromatin remodelers predicted as upstream regulators of transcriptional changes (*p* < 0.05) between Stress RE H3.3WT *vs*. Stress RE H3.3Q5A. Differences between groups were assessed using multiple two-sided unpaired Student’s t-tests with FDR correction (Benjamini-Krieger-Yekutieli; *q < 0.05). *n* = 5/group. Error bars represent median ± interquartile range. **e**) Schematic of working model. Panel **e** created in BioRender; Lab, M. https://biorender.com/4f8zyrj (2026). Source data

To resolve the precise genomic loci affected by H3.3(Q5A) expression, we performed H3K4me3Q5dop CUT&RUN-seq on infected dHF tissues. Remarkably, differential H3 dopaminylation sites that distinguished control RE H3.3 WT from stress RE H3.3 WT were reversed in stress RE H3.3(Q5A) (Fig. 5c). As expected from prior studies validating this approach^31–33,35,39^, the majority of dopaminylation peaks altered in stress RE H3.3(Q5A) were downregulated compared with stress RE H3.3 WT (Fig. 5d, Extended Data Fig. 10a and Supplementary Tables 16 and 17). These significantly reversed loci enriched for pathways involved in dopamine transport, glutamatergic signalling and learning (Fig. 5e), suggesting that H3 dopaminylation contributes to cognition-related behaviours. Similar to our chromatin data, bulk transcriptomic analyses in dHF showed that H3.3(Q5A) transduction reversed gene expression profiles between control RE H3.3 WT and stress RE H3.3 WT (Fig. 5f and Supplementary Table 18). DEGs from both comparisons overlapped with the RE-sensitive green modules, indicating that H3 dopaminylation modulates gene networks central to RE-associated plasticity (Extended Data Fig. 10b). Indeed, hierarchical clustering of all DEGs revealed that H3.3(Q5A) expression in stress RE dHF partially restores transcriptional patterns observed in control RE (Fig. 5g). To evaluate whether changes in H3 dopaminylation correspond to transcriptional alterations, we next intersected differential H3K4me3Q5dop binding sites with genes overlapping between control RE H3.3 WT versus stress RE H3.3 WT and stress RE H3.3 WT versus stress RE H3.3(Q5A) comparisons. Functional annotation analysis of these overlapping genes showed significant enrichment for learning-related pathways, including synaptic plasticity and glutamate signalling processes (Extended Data Fig. 10c). Because transcriptomic profiles at 49 dpp reflect both primary and secondary consequences of reducing H3 dopaminylation, we next examined upstream regulators that may contribute to the broader gene expression changes induced by H3.3(Q5A) transduction. We identified transcription factors predicted to regulate DEGs between stress RE H3.3 WT and stress RE H3.3(Q5A), including several shared with parous human dSub and our mouse model (Fig. 5h and Extended Data Fig. 10d), suggesting that H3 dopaminylation shapes RE-dependent plasticity by modulating not only target genes, but also transcription factors and chromatin remodellers that coordinate downstream expression.

Finally, we assessed whether reversing H3 dopaminylation in stress RE dams could influence contextual fear conditioning outcomes. Both control RE H3.3 WT and stress RE H3.3(Q5A) females showed enhanced acquisition compared to stress RE H3.3 WT (Fig. 5i). During the context test, all groups demonstrated recall, with control RE H3.3 WT and stress RE H3.3(Q5A) females showing higher freezing responses than stress RE H3.3 WT females (Fig. 5j). Together, these data directly link RE-dependent dHF transcriptional plasticity with persistent behavioural adaptations, and establish H3 dopaminylation as a key regulator of transcriptional networks impacted by RE and postpartum stress (Extended Data Fig. 10e).

## Discussion

Through controlled brain-wide, time-course and cell-type-specific transcriptomic analyses—paired with robust behavioural outputs—we identified gene networks, reproductive events and neuromodulatory-dependent epigenetic processes that dynamically shape regional sensitivity across pregnancy and postpartum experiences, thereby promoting plasticity long after these stages have ended. In particular, our study identified the dHF as a key site of sustained plasticity, consistent with its established roles in spatial cognition, novelty detection and sensory integration, which may bestow parous dams with heightened sensitivity to salient environmental cues relevant for resource foraging, nest navigation and offspring survival^41–44^. Consistent with our findings, lesion studies corroborate hippocampal involvement in pup retrieval behaviour^45,46^, potentially through downstream regulation of the NAc and ventral pallidum—key regulators of pup retrieval^44,47^. Notably, human studies show that maternal early life stress alters hippocampal responses to infant cues^48,49^, supporting chronic stress as a common interferer in the normal trajectory of maternal dHF neuroadaptations. Building on this, our maternal–pup separation stress model revealed that dopamine dynamics are essential for maintaining maternal adaptations in the dHF. Indeed, we established the necessity and sufficiency of maintaining appropriate dopamine tone for dHF plasticity, demonstrating that chronic dopamine elevation through maternal stress and chemogenetic suppression within virgin dHF bidirectionally modulate RE-related changes at transcriptional, epigenetic, behavioural and cellular levels. Moreover, we establish conserved transcriptional and epigenetic changes in the human dSub, a subregion of the dHF. In our postpartum stress mouse model, reversing aberrant changes in H3 dopaminylation was sufficient to restore RE adaptations. These findings implicate dopamine as a conserved driver of maternal brain remodelling, linking its transient roles during pregnancy and postpartum to enduring neuroplasticity in critical brain regions, including the dHF.

Although our study focused largely on dopamine, it is notable that our chemogenetic and viral manipulations recapitulated key transcriptional and behavioural outcomes of RE, albeit not to the same extent. This underscores dopamine as a crucial but non-exclusive driver of RE-induced adaptations. Indeed, our data also implicate hormonal contributions, including oestrogen, progesterone and oxytocin signalling, in shaping transcriptional programming. Consistent with this idea, previous studies have linked parity to oestradiol-driven neuronal alterations^12^, whereas others have demonstrated that blocking oxytocin signalling disrupts RE-induced spatial learning enhancements^21^, supporting an integration of multiple neuromodulatory pathways. However, given that oestrogen signalling during postpartum regulates oxytocin release, which in turn modulates VTA signalling, we propose that dopamine adaptations serve as a key downstream mechanism within a broader network driving sustained maternal neuroplasticity. Moreover, although we aimed to parse the contributions of individual events to RE-dependent dHF programming, fully disentangling these factors remains experimentally challenging. Mating that results in pregnancy may exert distinct effects from mating that does not result in pregnancy, and isolating pregnancy, birth or lactation would require invasive procedures that introduce confounds for brain outcomes. Nevertheless, our data indicate that pregnancy induces substantial dHF transcriptional changes, although they are not characteristic of the full composite of RE, emphasizing the additional contribution of healthy postpartum exposures to dHF programming. This raises the possibility that restoring elements of healthy postpartum exposure, through pharmacological interventions or pup-associated sensory cues, may mitigate disruptions imposed by postpartum stress. More broadly, our work suggests that RE drives a fundamental shift in brain state that may interact with additional experiences. As parity is implicated in both risk and resilience to brain disorders^50^, future research should consider parity status as a key variable shaping differential outcomes, particularly in its interactions with other risk factors. In particular, our findings highlight an interplay between RE and stress in shaping maternal brain outcomes, revealing epigenetic mechanisms and neuromodulatory pathways that underlie long-lasting adaptations, and underscore the importance of stress mitigation during pregnancy and postpartum.

## Methods

### Human participants

Brain tissues used in this study were provided by the Douglas Brain Bank (RRID:SCR_025991) with ethical approval from the Research Ethics Board (REB) of the Centre Intégré Universitaire de Santé et de Services Sociaux (CIUSSS) de l’Ouest-de-l'Île-de-Montréal. Analyses were conducted in accordance with all relevant guidelines and regulations. Informed consent from next of kin was obtained for each individual included in this study. Psychological autopsies, considered the gold standard for obtaining information on deceased individuals^51,52^, were conducted. In brief, these consist of a series of proxy-based, structured interviews assessing psychopathology with next of kin and complemented by reviews of medical records, as previously described^51^. Groups were matched for depression diagnosis, and were otherwise neurotypical individuals who died suddenly without prolonged agonal periods and did not have evidence of axis I disorders. Groups were matched for postmortem interval, tissue pH and RNA Integrity number. Frozen histological grade samples of grey and white matter were dissected from the subiculum by expert neuroanatomists and stored at –80 °C. Dissections were performed on 0.5-cm-thick coronal sections with the guidance of a human brain atlas^53^ (http://www.thehumanbrain.info/brain/bn_brain_atlas/brain.html). Subiculum samples were obtained from sections equivalent to plate 43 of the atlas (level of lateral geniculate nucleus), by dissecting through the hippocampal fissure with a slight upward angle, up to the beginning of the CA1 region. All procedures complied with ethical regulations for research involving human tissue. Demographic information can be found in Supplementary Table 11.

### Animals

Wild-type C57BL6/J mice were purchased from Jackson Laboratories at 8 weeks old, and maintained on a 12 h:12 h light:dark cycle throughout the entirety of the experiments. Mice were housed in temperature- and humidity-controlled conditions (approximately 22 ± 2 °C and 50 ± 10% humidity) with ad libitum access to food and water throughout the entirety of the experiments. All behavioural testing occurred during the light cycle. Experimenters were blind to experimental group, and the order of testing was counterbalanced during behavioural experiments. All animal procedures were performed in accordance with NIH guidelines and with the approval of the Institutional Animal Care and Use Committee of the Icahn School of Medicine at Mount Sinai.

### Breeding

Adult virgin female mice were pair bred in house with age-matched males. Males were removed after a maximum of 5 days, and pregnant females were singly housed at least 2 days before parturition. Pups were counted on the day of birth (0 dpp) and weaned at 21 dpp. Only dams with litters between 4–10 pups were used for all experiments. On the day of weaning, dams were group-housed into cages of 3–5 mice with animals of the same experimental condition. For timed breeding, copulation plugs were checked every morning within 1 h after lights on, where confirmation of a plug was designated as embryonic day 0.5 (E0.5), signalling the immediate removal of the female to her own cage with a nestlet. Virgin NP females were age-matched for each experimental cohort. Mating + no pregnancy females were confirmed for the presence of a copulation plug. For mating + pregnancy + birth dams, pups were removed at 0 dpp within 3 h after lights-on to minimize maternal-offspring interactions. For comparison of RE dams to pup sensitized virgin females, litters were culled to 4 pups to equate litter size with the number of pups used for each sensitized female. In all other experiments, litters were not culled.

### Postpartum stress paradigm

Stress RE females were subjected to limited nesting and maternal separation from 10–20 dpp (late stress RE) or 2–12 dpp (early stress RE), as previously described^54–56^. On each day of separation, the entire litter was removed to a clean cage with Sani-Chip bedding for 3–4 h. Separations occurred during the light cycle, and the timing varied each day to minimize predictability and acclimatization. EnviroDri nesting material was depleted to one-third of the amount in control cages during the days of separation. Following pup weaning on 21 dpp, the nesting material was restored to normal levels, and dams were group-housed into cages of 3–5 with mice of the same experimental condition.

### Pup sensitization

Pup sensitization was conducted as previously described^16,57^. On the first day of pup sensitization, virgin NP females were presented with 4 pups (postnatal day 5) from a cage consisting of a lactating donor dam and her litter. Donor pups were exchanged for satiated pups from the same litter every 8–12 h for 21 days. Donor pups were weighed daily to ensure continual weight-gain throughout the experiment, and only pups that exhibited consistent weight gain were used. Behavioural observations were conducted during the first four days of sensitization. Each dam was observed for 30 min during the light phase for the following maternal behaviours: licking/grooming, crouching and nestbuilding. During these observation periods, females were closely monitored to ensure that they did not display aggressive behaviour toward the donor pups. On the last day of sensitization, donor pups were weaned from the cage, and sensitized females were group-housed into cages of 3–5 mice with animals of the same experimental condition.

### Brain tissue collection

Mice were euthanized by rapid decapitation. Whole brains were flash-frozen with cold 2-methylbutane and stored at −80 °C until further processing. Flash-frozen brains were sectioned at −20 °C using a 1 mm mouse coronal brain matrix (Stoelting). Tissues enriched for the brain region of interest were micropunched using a hollow needle (Ted Pella) according to the Allen Brain Atlas. Brain regions were selected based on previous work supporting their involvement in maternal behaviours^58,59^.

### Behavioural analyses

All mice (18–30 weeks, depending on the time from pup weaning) were handled for 2 min for two consecutive days prior to initial behavioural testing. Mice were habituated to the testing room for 1 h prior to each behavioural assay. All testing occurred during the light phase.

#### Pup retrieval

All mice were individually housed for 24 h prior to testing. Following habituation, 2 pups from a donor litter with a lactating dam (aged 4–6 dpp) were placed in different corners opposite to the nest in the home cage. Pup-directed interactions were recorded from above for 15 min or until both pups were successfully retrieved into the nest. Retrieval latency was calculated as [(time pup was first placed into the nest by mouse) − (time first pup was placed into the cage by experimenter)].

#### Contextual fear conditioning

On day 1, mice were habituated for 10 min to the testing chamber, which consisted of a square plexiglass box with a metal grid inside a sound-attenuating cabinet wiped with 70% ethanol (Med Associates). On day 2, following a 3 min baseline measurement, mice were trained with 5× 2.0 s, 0.7 mA foot shocks delivered with an intertrial interval of 90 s. Testing for conditioned fear responses (freezing) for a total of 5 min occurred 24 h following training. Freezing was measured using ANY-maze software (v7.51) connected to a camera positioned above the testing chamber. Freezing is expressed as a percentage of the total test time or as a percentage of the 60 s prior to each shock during conditioning. Mice with freezing levels exceeding 40% prior to the first shock were excluded to remove potential confounding effects of heightened baseline responsivity that could interfere with accurately assessing conditioned fear.

#### Open field

Mice were placed in a 16 × 16 cm square arena under dim lighting for 5 min. A camera positioned overhead recorded the total distance and time spent in the centre versus periphery using Ethovision XT 11 software.

#### Object location task

For training, mice were allowed to freely explore two identical objects placed equidistant from adjacent corners of a 16 × 16 cm square arena for 5 or 10 min, before being returned to the home cage. Following a 1 h or 24 h delay, the mice were returned to the arena for testing, during which time one object was moved to the opposing corner. During the test, the mice were allowed to freely explore the objects for 5 min. A camera positioned overhead recorded time spent exploring each object using Ethovision software. The discrimination score was calculated as [(time spent with moved object) – (time spent with unmoved object)]/[(time spent with moved object) + (time spent with unmoved object)].

#### Light–dark box

Anxiety-like behaviour was tested in an apparatus containing two interconnected 20 × 20 cm compartments (Omnitech Electronics). One compartment was illuminated during the session (‘light’ side), while the other was covered by an opaque black perspex lid (‘dark’ side). The distance, time spent, and number of crossovers in each compartment were automatically recorded by Fusion software during the 10 min test.

#### Forced swim test

Mice were placed in a 4 l glass beaker with 2 l of room temperature water for 8 min. Each session was recorded and scored by a blinded observer. The total number of seconds that mice were immobile during the last 5 min of the test were recorded, as previously described^60^.

#### Observation of pup-directed behaviours in the home cage

Confirmation of maternal behaviour in pup sensitized females was conducted based on prior studies^61^. Mice were observed from the first to fourth day of pup sensitization, based on published work that maternal sensitization in C57BL6/J females occurs following 4 days of pup exposures^62,63^. Observations occurred in the light period in the first 30 min after pups were placed in the cage. Each mouse was scored every 3 min, with the observed behaviour recorded as one or more of the following categories: nestbuilding, grooming, sniffing, crouching, nursing, eating, drinking, self-grooming or no-contact. RE dams were scored for the first 4 days following birth (0–3 dpp) concurrently for comparison.

### Oestrous cycle testing

Vaginal samples were taken on each day of behavioural testing. Fifteen microlitres of sterile PBS was gently pipetted into the vagina and mounted on a glass slide. Vaginal smears were stained with crystal violet dye, washed twice with water, and cover slipped with glycerol. Three 20× images per sample were acquired on a light microscope. Oestrous stage was determined using the pretrained network of EstrousNet^64^, and confirmed afterwards by a trained experimenter based on cytology of nucleated, cornified, or leukocytic cells. *P* values from Fisher’s exact tests confirming that oestrous stage distributions did not differ across groups are provided in Supplementary Table 19. All staging information is available in the Source Data.

### Viral transduction

Mice were anaesthetized with ketamine (100 mg kg^−1^) and xylazine (10 mg kg^−1^) intraperitoneally and positioned in a stereotaxic frame (Kopf instruments). Given the widespread changes in dopamine receptor expression across dHF subregions, we did not restrict our viral manipulations to a specific subregion. For chemogenetic studies, 2 µl of retrograde AAV.rTH.PI.Cre.SV40 (titre ≥ 7 × 10^12^ viral genomes (vg) per ml, Addgene #107788-AAVrg) was infused bilaterally into the dHF at 0.2 μl min^−1^ using the following coordinates: 7° angle; anterior-posterior (AP) −2.2 mm, medial-lateral (ML) ±2.0 mm, dorsal-ventral (DV) −2.0 mm. One microlitre of pAAV-hSyn-DIO-mCherry (titre ≥ 4 × 10^12^ vg ml^−1^, Addgene #50459-AAV2) or pAAV-hSyn-DIO-hM4D(Gi)-mCherry (titre ≥ 5 × 10^12^ vg ml^−1^; Addgene #44362-AAV2) was bilaterally infused into the VTA using the following coordinates: 7° angle; AP −3.3 mm, ML ±0.9 mm; DV −4.6 mm. For rescue of H3 dopaminylation, pAAV2-CMV-H3.3WT-IRES-GFP or pAAV2-CMV-H3.3(Q5A)-IRES-GFP viruses were generated and validated as previously described^31–33,35,39^. Approximately 3–5 days post-weaning, mice were prepared for surgery, as described above, and 1 µl of virus was bilaterally infused into the dHF using the same coordinates at a rate of 0.1 μl min^−1^. Needles remained in place for 7 min following injection to minimize virus diffusion. Viral validations were conducted at least 21 days post-surgery to allow for optimal viral expression and recovery.

### Chemogenetic manipulation

Following 7 days of recovery, surgerized female mice were randomly assigned to NP or RE groups. RE females were pair bred with naïve male mice for a maximum of 5 days, and individually housed prior to parturition. From 10–20 dpp, RE females were injected subcutaneously with DCZ (Tocris 7193)—to minimize off-target effects^65^—at 1 μg kg^−1^ in 1% DMSO or vehicle (saline). NP females were injected with DCZ concurrently. Following weaning on 21 dpp, RE dams were group-housed with mice of the same experimental condition. Behavioural testing occurred beginning at 28 days post-weaning (49 dpp), and brain tissues were collected following contextual fear conditioning for further analyses.

### RNAscope in situ hybridization and analysis

Fresh frozen brains were cut into 12–16-μm-thick slices in the coronal plane with a cryostat (Leica CM3050-S), mounted on charged Superfrost Plus microscope slides (Thermofisher P36934), and stored at −80 °C until processing. Sections were post-fixed with 4% paraformaldehyde (PFA) for 1.5 h at 4 °C, and permeabolized with hydrogen peroxide (10 min, room temperature) and Protease III (30 min at room temperature). The RNAscope Multiplex Fluorescent Reagent Kit v2 (ACD Bio) was used according to manufacturer’s instructions to sequentially stain sections with the following probes: *Drd1a* (Mm-Drd1a-C1, 461901) and *Drd2* (Mm-Drd2-C3, 406501-C3). RNAscope probes were visualized using TSA Vivid Fluorophores (Tocris) at 1:750 dilution. Sections were counterstained with DAPI (ACD Bio) and mounted using ProLong Gold Antifade Mountant (Thermo). Confocal images (3 images per mouse, 1,024 × 1,024 pixels) were acquired on a Zeiss LSM 780 upright microscope using a 40× objective with Zen Black software, with 5 × 1 tiled images. Images were averaged across 8 consecutive acquisitions at a bit depth of 16 bits, with 2 *z*-stacks acquired per image. Subcellular quantification of individual puncta per 100 μm nucleus—identified by DAPI staining—was performed for each maximum intensity projected image using QuPath software (v0.5.1)^66^. Regions of interest (CA1 and DG) were annotated and determined by superimposing images onto the Allen Brain Atlas. Linear mixed-effects models were used to assess group-, region-, and probe-dependent effects on puncta density. For each cell, puncta density (puncta per 100 μm^2^ nuclear area) was modelled with Group, Region, and Probe as fixed effects, including all interaction terms, and mouse identity was included as a random intercept to account for within-animal clustering of cells. Type III tests with Satterthwaite’s approximation were used for inference. Following the mixed-effects analysis, the distribution of puncta within cells was compared across groups. Cells were classified into low (<1), medium (1–5), or high (>5 puncta) expression bins. For each region, contingency tables crossing expression bin with Group were analysed using Pearson’s chi-square test. Standardized Pearson residuals were used to identify bins that were over- or under-represented in each group. For image source data, see Supplementary Fig. 2.

### Immunofluorescence and analysis

Mice were anaesthetized with isoflurane and perfused with cold 1× PBS followed by 4% PFA. Brains were post-fixed in 4% PFA overnight and then transferred to a 30% sucrose/PBS solution for 2 days. Brains were sectioned at 40 µm thickness using a Leica CM3050-S cryostat, with serial sections collected from the VTA. For each subject, 2–3 brain slices were blocked for 2 h (0.1% Triton X-100, 10% normal donkey serum), followed by overnight incubation at 4 °C with primary antibodies: chicken anti-tyrosine hydroxylase (1:500, Aves Labs TYH), rabbit anti-FOS (1:2,000, Synaptic Systems 226-008), mouse anti-HA (1:1,000, Abcam ab18181), and/or rabbit anti-H3K4me3Q5dopaminyl (1:250, Millipore ABE2590). The next day, slices were incubated for 2 h at room temperature with fluorescent-conjugated secondary antibodies (1:1,000, donkey anti-chicken Alexa Fluor 488, Invitrogen A78948; goat anti-rabbit Alexa Fluor 546, Invitrogen A11010; donkey anti-goat Alexa Fluor 680, Invitrogen A10043; donkey anti-mouse Alexa Fluor 680, Invitrogen A10038). Slices were counterstained with DAPI (1:10,000, Thermo Scientific 62248) and mounted with ProLong Gold Antifade Mountant (Thermo Fisher P36934). Confocal images (2–3 replicates per mouse, 1,024 × 1,024 pixels) were acquired on a Zeiss LSM 780 upright microscope using a 40× objective with Zen Black software. Images were averaged across 8 consecutive acquisitions at a bit depth of 16 bits. Image analysis was conducted using FIJI software (NIH). The FOS and TH channels were thresholded using the MaxEntropy method to define regions of interest. Following identification of colocalized FOS^+^/TH^+^ signal, FOS intensity was measured and averaged from 4–6 images per mouse. Brightness and contast were adjusted for representative images. For image source data, see Supplementary Fig. 3.

### Dopamine ELISA

Brain tissue dopamine levels were assessed in response to 3 h of pup separation in the home cage. Independent biological replicates were collected at each time point, with separate mice used for each measurement. Brains were rapidly collected (see ‘Brain tissue collection’) at baseline (prior to pup removal, 0 min), during pup removal (30 and 180 min), and 30 min after pups were returned to the home cage (210 min). Equal weights of micropunched brain tissues were homogenized in lysis buffer (0.01 N HCl, 1 mM EDTA, 4 mM sodium metabisulfite). Tissue dopamine levels were assessed using the Dopamine (Research) ELISA Kit (ALPCO Diagnostics) according to manufacturer’s instruction.

### HPA axis assessment

Plasma corticosterone levels were assessed in response to 3 h of pup separation in the home cage. Testing was initiated within 2 h after lights on. Tail blood was collected prior to pup removal (0 min), during pup removal (30 and 180 min), and 120 min after pups were returned to the cage (300 min) from the same mice. Samples for control mice in the home cage, used to account for the effects of handling-induced stress, were collected concurrently. Blood samples were immediately mixed with 50 mM EDTA and centrifuged at 5,000 rpm for 10 min. Plasma was collected and stored at −80 °C until analysis. Corticosterone levels were quantified using a Corticosterone ELISA kit (ENZO Life Sciences) according to manufacturer’s instruction.

### Clonal *TGM2* knockout in HeLa cells

HeLa cells (ATCC, CCL-2) were cultured at 37 °C with 5% CO_2_ in DMEM medium (high-glucose, ThermoFisher 11965118) supplemented with 10% FBS (Sigma-Aldrich) and 500 U penicillin and streptomycin. The CRISPR guide RNA targeting exon 5 of *TGM2* was purchase from IDT, containing the sequence ACGCTGGGACAACAACTACG. 120 pmol of guide RNA and 100 pmol of Alt-R *Streptococcus pyogenes* Cas9 Nuclease V3 (IDT, 1081058) were premixed for 15 min in 5 μl total (with PBS). HeLa cells (200,000) were washed in PBS, before resuspending in 20 μl of nucleofector solution (SE Cell Line 4D-Nucleofector X Kit S, Lonza V4XC-1032). The 5 μl Cas9/sgRNA mix was added, as well as 1 μl of Alt-R Cas9 Electroporation Enhancer (IDT, NC1395977), and all mixed gently. The entire mixture was transferred to a 16-well Nucleocuvette strip (Lonza, PDH-2104) gently, and nucleofected using the Lonza 4D-NucleofectorUnit, using the default settings for HeLa cells. Directly after nucleofection, 80 μl of prewarmed culture media was added to the cuvette. The entire mixture was immediately transferred to a 6-well plate with 2 ml of prewarmed culture media, and incubated cultured at 37 °C with 5% CO_2_ for 48 h. Nucleofection efficiency was assessed by using a second positive control reaction with a pMax-GFP plasmid (Addgene, 177825). The pool of cells was diluted to a concentration of 1 cell per 200 μl, and 100 μl was aliquoted into each well of 10× 96-well plates, and cultured at 37 °C with 5% CO_2_. After 14 days, single-clones were identified using a light microscope. Single-clone containing wells were expanded and targeting assessed by extracting genomic DNA and performing PCR and PCR sequencing over the targeted site (forward: GGCTCCAGCCCCCACCATCTGCCGCAC; reverse: GCCACATAGCGCATTGAGAGTGTTGGT). PCR sequencing results were assessed using Synthego’s ICE analysis. Clones which were identified as introducing premature stop codons were expanded further.

### Assessment of *TGM2*-knockout cell line

#### Western blot for TG2

In brief, HeLa cells were collected and lysed using high-salt buffer (20 mM HEPES pH 7.9, 500 mM KCl, 10 mM MgCl_2_, and 1 % NP-40), followed by brief pulse-sonication. One-hundred micrograms of protein was run on a 4–12% Bis-Tris gel (Invitrogen, NW04122) for 45 min at 150 V. Protein was transferred to a 0.2 µm nitrocellulose membrane using a Trans-Blot Turbo Transfer System (Bio-Rad) following the manufacturers protocol. The membrane was blocked in 5% milk in TBS for 1 h, and primary antibody (anti-TG2, Abcam 2386, 1:500 in 1.5% milk in TBS) overnight at 4 °C. The blot was washed 3× 15 min in TBS-T, and then incubated with secondary antibody (Goat anti-Mouse IgG (H + L) Cross-Adsorbed Secondary Antibody, Alexa Fluor 647, Invitrogen A-21235) at room temperature for 1 h, followed by washing 3× 15 min in TBS-T. The blot was imaged using a Bio-Rad ChemiDoc MP, and then the blot was stained with amido-black stain to assess total protein loading.

#### Transamidation activity assay

One-hundred micrograms of HeLa extract was diluted in low-salt buffer (20 mM HEPES pH 7.9, 150 mM KCl, 10 mM MgCl_2_), and 1× final transamidation assay buffer was added (25 mM Tris-HCl, pH 8, 10 mM CaCl_2_, 10 mM DTT, 10 mM KCl). Biotin-cadaverine (Millipore Sigma A5348) was added to a final concentration of 1 mM, and reactions incubated at 30 °C for 2 h. A western blot was run as described above, using a Streptavidin-Alexa Fluor 488 Conjugate (ThermoFisher S32354) to measure incorporation of biotin-cadaverine.

### Statistics

Statistical analyses for behavioural and immunoassay data were conducted using Prism software (GraphPad, v10.4.1). Data distribution was assessed for normality. Data that met assumptions of normality were analysed using parametric tests, while non-normally distributed data were analysed using non-parametric alternatives. For experiments involving multiple conditions, one-way or two-way ANOVAs were performed, followed by post hoc analyses when appropriate. For time-course analyses where multiple measurements were taken from the same animal, repeated measures ANOVAs were performed. Two-tailed Student’s *t*-tests were used for comparisons between two conditions. Behavioural data derived from manual observations and pregnancy/litter outcomes were analysed using chi-square tests. Grubb’s test (alpha = 0.05) was applied to detect outliers where necessary. Statistical significance was defined as *P* ≤ 0.05.

### Bulk RNA-seq and analysis

#### RNA isolation and library preparation

Total mRNA was extracted from frozen brain tissues after homogenization in Trizol Reagent (Thermo Fisher) and cleaned using RNeasy Microcolumns (Qiagen) following the manufacturer’s instructions. For RNA-seq library preparation, 150 ng of mRNA per sample was used with either the Illumina Stranded mRNA Prep Kit (Illumina, 20040534) or the TruSeq RNA Library Prep Kit v2 (Illumina, RS-122-2001), according to the manufacturer’s protocols. Library quality was assessed using a Qubit Fluorometer 2.0 (Thermo Fisher) and a High Sensitivity D5000 TapeStation assay (Agilent) before sequencing on a NovaSeq 6000 or NovaSeq X system.

#### Differential expression analysis

Raw fastq files, containing an average of 20–30 million reads per sample, were processed for pseudoalignment and abundance quantification using Kallisto (v0.46.1) against the Ensembl Mus musculus reference (v79)^67^. To filter lowly expressed genes, only those with a total read count of at least ten across all samples were retained. To account for unwanted variation among samples within each sequencing experiment that could arise from technical or biological factors unrelated to the conditions of interest (including litter size, oestrous stage, day of sample collection, etc.), RUVs (v1.32.0) was applied with a negative control gene set derived from the total genes identified per sequencing experiment, after ensuring that unwanted variation did not correlate with covariates of interest, as described previously^68,69^. Differential expression analysis was performed using DESeq2 (v1.38.3)^70^, with significant genes defined by an adjusted *P* value < 0.05. For brain-wide transcriptome comparisons in which samples were processed across multiple sequencing runs and stemming from separate cohorts, pairwise comparisons were performed independently for each brain region. For all other experiments, where individuals came from the same cohort and were processed in a single sequencing run, all groups were analysed together to maintain consistent normalization within the experiment. Gene expression time-course analyses examining the periods before, during, and after pregnancy and postpartum were performed on normalized count data using the ImpulseDE2 package (v0.99.10)^71^ for each brain region. Significant genes exhibiting transient regulation or monotonous changes in expression were identified using case-only differential expression analysis, with a Q-value threshold of 0.05. To adjust for cell-type composition in the DESeq2 model while avoiding multicollinearity and preserving degrees of freedom, principle components analysis was performed on the scaled surrogate proportion values (obtained from BRETIGEA) for the entire dataset^72,73^. The first two principle components (PC1 and PC2) were incorporated into the DESeq2 design formula, with the number of RUVSeq factors (*k*) reduced by two to accommodate these additional covariates.

#### WGCNA

To identify brain-wide gene co-expression networks, normalized count data for all brain regions were compiled and analysed using the WGCNA package (v1.73)^74^. Co-expression networks were constructed from the 7,500 most variable genes, determined by ranking gene variance. A soft-threshold power of 12 was identified with the pickSoftThreshold function to ensure scale-free network properties. Modules were identified based on dissimilarity of a signed topological overlap matrix, and named with an arbitrary colour. The ‘grey’ module encompassed genes that did not segregate into any specific module, and was therefore removed from further analyses. To assess the enrichment of DEGs within each brain region per module, Fisher’s exact tests were performed using the fisher.test() function in R (v4.3.0). To analyse the correlation between gene modules and brain regional sensitivity to parity, brain regions were categorized into two groups based on the top five regions with significant DEG overlap. These regions were designated as high-sensitivity regions, while all other regions were classified as low-sensitivity. Pearson correlation coefficients were calculated to examine the relationship between each module and the trait (regional sensitivity × parity status), with statistical significance determined by Student’s *t*-tests (*P* < 0.05). Heat maps were generated to visualize these module–trait correlations, and module-specific gene lists were exported for pathway enrichment analysis.

#### Pathway and predicted upstream regulator analyses

Functional annotation of DEGs was conducted using ShinyGO (v0.81)^75^, with all protein-coding genes in the mm10 genome used as the background. Pathways with an FDR < 0.05 were considered significant. Relevant pathways were selected from the top 10 or one-third of significant terms, ranked by FDR, to emphasize processes consistent with hypotheses informed by published literature. For GO term selection, Revigo^76^ was used to reduce redundancy of overlapping GO terms when needed. Ingenuity Pathway Analysis (IPA; Qiagen v01-23-01) was used to predict upstream regulators for DEG lists^77^. For high-sensitivity and low-sensitivity regions (see ‘WGCNA’ section), DEGs shared by at least two or three brain regions were extracted, irrespective of the direction of change. The IPA software was used to identify upstream regulators associated with each DEG list, with statistical significance defined as *P* < 0.05. To investigate the mechanisms underlying parity programming of dHF plasticity, significant upstream regulators were systematically prioritized based on one or more of the following criteria: (1) multiple molecules involved in the same signalling pathways (for example, progesterone and its receptor, PGR); (2) structurally similar molecules that engage analogous signalling cascades (for example, levodopa and dopamine); (3) molecules known to be influenced by reproductive exposures; (4) molecules demonstrated to play a role in hippocampal plasticity; (5) molecules identified as significant in both high-sensitivity and low-sensitivity regulator lists; and (6) molecules with high statistical significance values. Following selection, upstream regulators were categorized into general molecular classes (for example, ‘hormone’, ‘transcription factor’ or ‘lipid’) and grouped for visualization. Data are presented as a bubble plot, with marker shape representing number of brain regions sharing DEGs and marker size corresponding to significance.

#### Gene expression overlap analyses

Jaccard indices were calculated for overlapping gene lists using the GeneOverlap package (v1.36.0), with significance defined by *p* < 0.05. Fisher’s exact tests were calculated using the base fisher.test() function in R (v4.3.0) following construction of contingency tables for each comparison. Transcriptome-wide, threshold-free gene expression overlap was visualized using RRHO heat maps generated with the RRHO2 package (v1.0)^78^. Gene lists were ranked by signed *P* values, calculated as the log_10_-transformed nominal *P* value multiplied by the sign of the fold change, without applying differential expression thresholds.

#### Cell-type deconvolution

Brain cell-type proportion was estimated from normalized expression data using the BRETIGEA package (v1.0.4)^79^ with default markers. For comparisons between groups, surrogate proportion variables were normalized using the Normalize function in Prism (GraphPad v10.4.1). Each sub column was normalized separately, with the smallest value set to 0% and the largest value set to 100%. To calculate log_2_(fold change) between groups, normalized values per cell type per group were averaged and expressed as the log_2_-transformed ratio of the group means.

### snRNA-seq

#### Nuclei isolation and library preparation

For each mouse, 2 mm dHF-enriched tissue micropunches were collected bilaterally from consecutive 1 mm slices from −0.80 to −2.80 mm relative to bregma for a total of 4 punches (Ted Pella). Samples were processed in batches of 4–6, with each group represented within each batch. Nuclei were isolated using a modified version of a sucrose density gradient isolation protocol^80^. In brief, thawed tissues were placed in 1 ml of lysis buffer (0.32 M sucrose, 5 mM CaCl_2_, 3 mM magnesium acetate, 0.1 mM EDTA, 10 mM Tris-HCl pH 8, 1 mM DTT, 0.1% Triton X-100) with 50 μl of 25 U ml^−1^ RNase inhibitor (Takara 2313B) in a dounce homogenizer (Wheaton 357538). Homogenization was performed with 20 strokes using a tight pestle. Another 1 ml of lysis buffer was added, followed by an additional 10 strokes. The resulting 2 ml homogenate was transferred to a 15 ml Open-Top Thinwall Polypropylene Tube (Beckman-Coulter 361707). The homogenizer and pestle were rinsed with 2 ml of lysis buffer, and this wash was combined with the homogenate for a total of 4 ml. The homogenate was carefully underlaid with 9 ml of sucrose solution (1.8 M sucrose, 3 mM magnesium acetate, 1 mM DTT, 10 mM Tris-HCl pH 8) and ultracentrifuged at 24,000 rpm for 1 h at 4 °C using a Sorvall WX+ centrifuge. After centrifugation, the supernatant and debris at the interface were gently removed. The nuclear pellet was resuspended in 1 ml of resuspension buffer (0.02% bovine serum albumin in DPBS with 25 μl of 25 U ml^−1^ RNase inhibitor) and incubated on ice for 10 min. The suspension was passed through a 35 μm nylon mesh filter (Corning 352235) into a 1.5 ml RNase/DNase-free microcentrifuge tube and centrifuged at 2,600*g* for 10 min at 4 °C. Supernatants were discarded, and nuclei were resuspended in 200 μl of resuspension buffer. A 10 μl aliquot of the nuclei suspension was stained with Trypan Blue to assess quality and concentration using a haemocytometer. Nuclei suspensions were loaded onto a Chromium Single Cell 3′ chip (10X Genomics, v3) and processed according to the manufacturer’s protocol, targeting 10,000 nuclei. Single-nuclei libraries were generated using the 10X Chromium Next GEM Single Cell 3′ v3.1 (Dual Index) protocol (CG000315 Rev A). Libraries were pooled, loaded onto a single 10B 100 Cycle Flowcell, and sequenced using an Illumina NovaSeq 6000 system to generate 25,000–30,000 paired-end 2× 100 bp reads per cell.

#### Data analysis

FastQ files were processed with the 10X Genomics Cell Ranger pipeline (v7.1.0) to demultiplex reads, align them to the mouse genome (mm10-2020-A), remove PCR duplicates, and generate gene expression matrices. Cell Ranger filtered outputs were analysed using Seurat v4.3.0^81^, and mitochondrial RNA content per cell was calculated using the GRCm39 (mm10) genome annotation and regressed out using SCTransform normalization protocol included in the Seurat toolkit with 20 principal components and a resolution of 0.1. To estimate ambient RNA and correct for background contamination, the SoupX (v1.6.2) package^82^ was used for each sample using raw and filtered feature matrices from the Cell Ranger output. Heterotypic doublets were identified and removed using DoubletFinder (v2)^83^ to ensure the integrity of singlet datasets. Filtered singlet datasets were then re-normalized and integrated using the same Seurat SCTransform v2 workflow mentioned above. Cell clusters were annotated using a combination of expert curation based on published marker genes^84–90^, and label transfer from hippocampal reference datasets, including the Allen Brain Map and Broad Institute resources^89,91^. Clusters with contaminant cell populations expressing markers for choroid plexus (*Ttr*), ependymal (*Tmem212*), and vascular leptomeningeal cells (*Vtn* and *Col1a2*) were removed from the analysis^90,92–94^. Additionally, as the sequential 2 mm micropunches encompassed portions of cortical, thalamic and vHF regions, clusters characterized by enrichment of published non-dHF neuronal markers using Seurat’s FindMarkers function (layer 5/6 cortical: *Rorb* and* Foxp2*^94^; ventral granule neurons: *Tox3*^86^) were also removed from the analysis. Cell cluster proportion analyses were conducted using the scProportionTest package^95^, which employs a Monte Carlo permutation test to evaluate whether observed differences result from random sampling. Proportional differences between conditions were compared to a null distribution generated by resampling, and statistical significance was determined by permutation-based *P* values with confidence intervals estimated via bootstrapping. Differential expression analysis was conducted using pseudobulk analysis, where gene counts were summed across all cells within each sample for each cell-type cluster using the AggregateExpression() function. DESeq2 was then applied at the sample level to conduct differential expression. Note that differential expression analysis could not be performed for the GABA.4 cluster due to insufficient sample representation in multiple groups. To explore pathways underlying cluster-specific differences across conditions, pathway analysis was conducted using ShinyGO on genes meeting the following criteria: log_2_(fold change) > 1.5 and *P* < 0.05.

### CUT&RUN-seq

The procedure was adapted from established protocols^33,96^. For mouse brain tissue, two 1.5-mm dHF punches were used for each biological replicate and split across two reactions (H3K4me3Q5dop and IgG). Samples were not pooled. For human brain tissue, ~10 mg of tissue dissected from individual frozen subiculum samples was collected from each subject, and split across the indicated antibodies. For HeLa cells, samples were split across H3K4me3Q5dop, H3K4me3 and IgG reactions. Samples were dounce homogenized in nuclear extract (NE) buffer (20 mM HEPES-KOH pH 7.9, 10 mM KCl, 0.5 mM spermidine, 0.1% Triton X-100, 20% glycerol with protease inhibitors) and passed through a 21 gauge needle 10 times. Nuclei were pelleted at 1,100*g* for 5 min at 4 °C in a swinging-bucket rotor, passed through a 40-μm strainer (pluriSelect), washed again in 500 μl NE buffer and counted. BioMag Plus Concanavalin A beads (Polysciences) were prepared per reaction by washing three times with binding buffer (20 mM HEPES-KOH pH 7.9, 10 mM KCl, 1 mM CaCl_2_, 1 mM MnCl_2_). Beads (15 μl) were aliquoted into 1.7 ml DNA low-bind tubes (Eppendorf) containing 500 μl NE buffer and 100,000 nuclei per reaction. Samples were rotated at room temperature for 10 min, then bead-bound nuclei were washed three times with wash buffer (20 mM HEPES pH 7.5, 150 mM NaCl, 0.1% Triton X-100, 0.1% Tween-20, 0.5 mM spermidine, 0.1% BSA, and protease inhibitors), resuspended in 100 μl antibody buffer (wash buffer with 2 mM EDTA), and mixed. 2 μl antibodies were added to the corresponding tubes: H3K4me3 (Active Motif, 39159), H3K4me3Q5dopaminyl (Millipore, ABE2590), or rabbit IgG (Invitrogen, 10500c). Samples were incubated overnight at 4 °C on a rotating mixer angled upward at 20 degrees. The next day, nuclei were washed twice with cold wash buffer, and incubated with 2.5 μl pAG-MNase (Epicypher, 15-1016) for 1 h at 4 °C. After 4 washes with cold wash buffer and one with low-salt rinse buffer (20 mM HEPES pH 7.5, 0.5 mM spermidine, 0.1% Tween-20, 0.1% Triton X-100), nuclei were resuspended in calcium incubation buffer (3.5 mM HEPES pH 7.5, 10 mM CaCl_2_, 0.1% Tween-20, 0.1% Triton X-100) and placed into an ice-cold block at 4 °C. MNase digestion was stopped by adding 100 μl of 2× Stop Buffer (340 mM NaCl, 20 mM EDTA, 5 mM EGTA, 0.1% Tween-20, 0.1% Triton X-100, 25 μg ml^−1^ RNase A, and 0.05 ng per 100 μl *Escherichia coli* spike-in DNA), followed by a 15 min incubation at 37 °C without shaking. Beads were then placed on a magnet and 200 μl of supernatant was collected. DNA was purified using the Zymo ChIP DNA Clean & Concentrator kit (D5205), eluted in 30 μl, and stored at −20 °C for library preparation. Libraries were generated using the NEBNext Ultra II DNA library kit, quantified using a Qubit fluorometer with the HS DNA kit, checked for size distribution on the Agilent TapeStation, pooled equimolarly, and sequenced on an Illumina NovaSeq X.

#### Data analysis

Raw fastq files were aligned to the hg19 or mm10 genome using bowtie2 (v2.5.0)^97^. Low-quality reads were filtered using Samtools (v1.9) with a MAPQ cut-off score of 30^98^. Only unique, deduplicated reads were retained for further processing. Bigwig files were produced using the deepTools package (v3.5.1), using an ENCODE hg19 or mm10 v2 blacklist file to discard regions with consistently non-specific signal, and scaled using *E. coli* spike-in controls to normalize sequencing depth. To determine normalization factors based on *E. coli* reads, each sample was aligned to the *E. coli* genome (MG1655), and the unique deduplicated reads were compared across groups per antibody per experiment. The sample with the lowest number of *E. coli* reads was determined (minimum), and all samples were scaled by dividing their corresponding *E. coli* read count by this minimum number^99^. For each group, bigwig files were merged and peak calling was conducted using MACS2 (v2.1.0) with the corresponding merged IgG file as control, and filtered for peaks with FDR < 0.05^100^. Peak annotation was conducted using HOMER (v4.1.1)^101^. Heat maps were made either using the DiffBind (v3.8.4) or deepTools (v3.5.5) packages^102^. For deepTools, heat maps were made by merging DEGs from RNA-seq data with TSSs downloaded from the UCSC genome browser using the canonically annotated transcript for each gene. Profiles were generated and statistically analysed using the deepStats package^103^ by using the dsCompareCurves function to perform Wilcoxon Rank-sum tests per-bin. For DiffBind analysis, heat maps were made for peaks identified by DiffBind’s differential peak algorithm, where differential peaks were first filtered using a log_2_(fold change) threshold > 0.1 and defined at p < 0.05, where log_2_(fold change) was calculated as log_2_(parity) − log_2_(NP), based on prior empirical observations used to define thresholds for differential peaks^36^. ChEA analysis on annotated loci was conducted using EnrichR with a significance threshold of adjusted *P* < 0.05 (ref. ^104^).

### Reporting summary

Further information on research design is available in the Nature Portfolio Reporting Summary linked to this article.

## Online content

Any methods, additional references, Nature Portfolio reporting summaries, source data, extended data, supplementary information, acknowledgements, peer review information; details of author contributions and competing interests; and statements of data and code availability are available at 10.1038/s41586-026-10509-4.

## Supplementary information

Supplementary Information This file contains Supplementary Figs. 1–3 and the legends for Supplementary Tables 1–19.

Reporting Summary

Supplementary Tables This file contains Supplementary Tables 1–19.

Peer Review File

## Source data

Source Data Figs. 1, 2, 4 and 5 and Source Data Extended Data Figs. 1, 2, 5–7, 9 and 10

## Data Availability

The genomics data generated in this study have been deposited in the National Center for Biotechnology Information Gene Expression Omnibus (GEO) database under the SuperSeries GSE298544. Reference genomes used in this study include the mouse genome mm10 and human genome hg19. Publicly available reference datasets used for annotation included resources from the Allen Brain Map and Broad Institute, with gene annotations obtained from the UCSC Genome Browser and ENCODE blacklist regions applied where appropriate. All data supporting the findings of this study are available within the article and Supplementary Information. Related data, including raw microscopy images, are available from the corresponding authors upon reasonable request. Source data are provided with this paper.

## Code availability

Data were analysed using publicly available software and standard pipelines, as described in the Methods. No custom code was generated for this study; however, any related analysis scripts or details are available from the corresponding authors upon reasonable request.
