# Supplementary material for: Dopamine drives persistent remodelling of the maternal brain
Source: Nature. 2026 May 20;654(8118):465–75. doi: 10.1038/s41586-026-10509-4 (PMC13253353; doi:10.1038/s41586-026-10509-4)
Supplement: Supplementary file 1 — This file contains Supplementary Figs. 1–3 and the legends for Supplementary Tables 1–19. [file 41586_2026_10509_MOESM1_ESM.pdf]

nature portfolio

https://doi.org/10.1038/s41586-026-10509-4

Supplementary information

Dopamine drives persistent remodelling of the maternal brain

In the format provided by the authors and unedited

Nature – **Research Article**

Supplementary Information

Dopamine drives persistent remodeling of the maternal brain

Jennifer C O'Chan, Giuseppina Di Salvo, Ashley M. Cunningham, Sohini Dutta, Elizabeth Brindley, Benjamin H. Weekley, Winnie Chen, Rasika R. Iyer, Ethan Wan, Cindy Zhang, Naguib Mechawar, Gustavo Turecki, Ian Maze

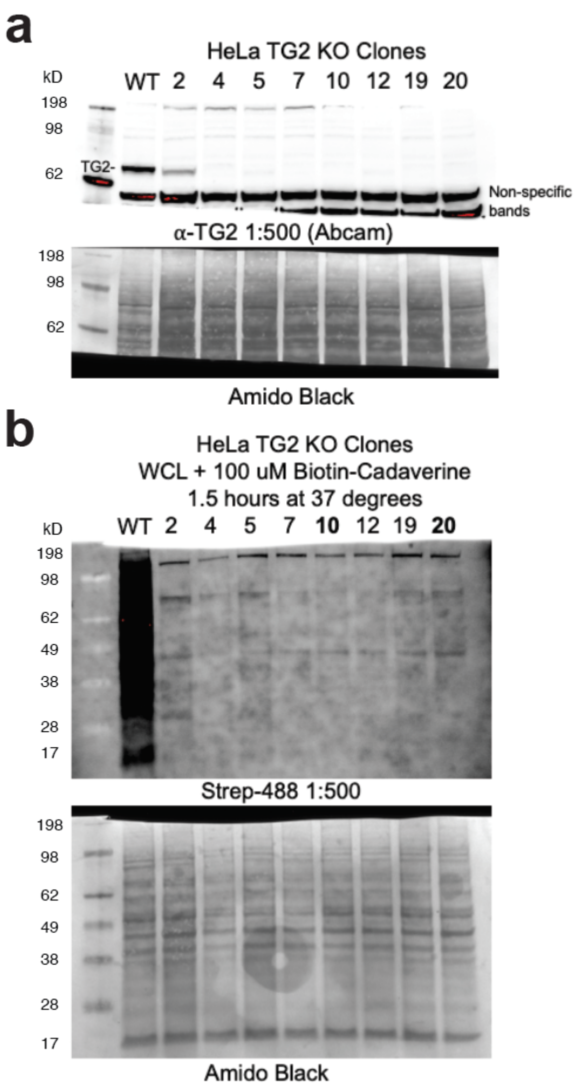

Supplementary Figure 1. Uncropped blots related to Extended Data Figure 8
Western blots related to **(a)** Extended Data Fig 8a, **(b)** Extended Data Fig 8b. The images shown in Extended Data Fig. 8 are presented in uncropped form. Amido Black staining, used as a loading control, was performed on the same blot.

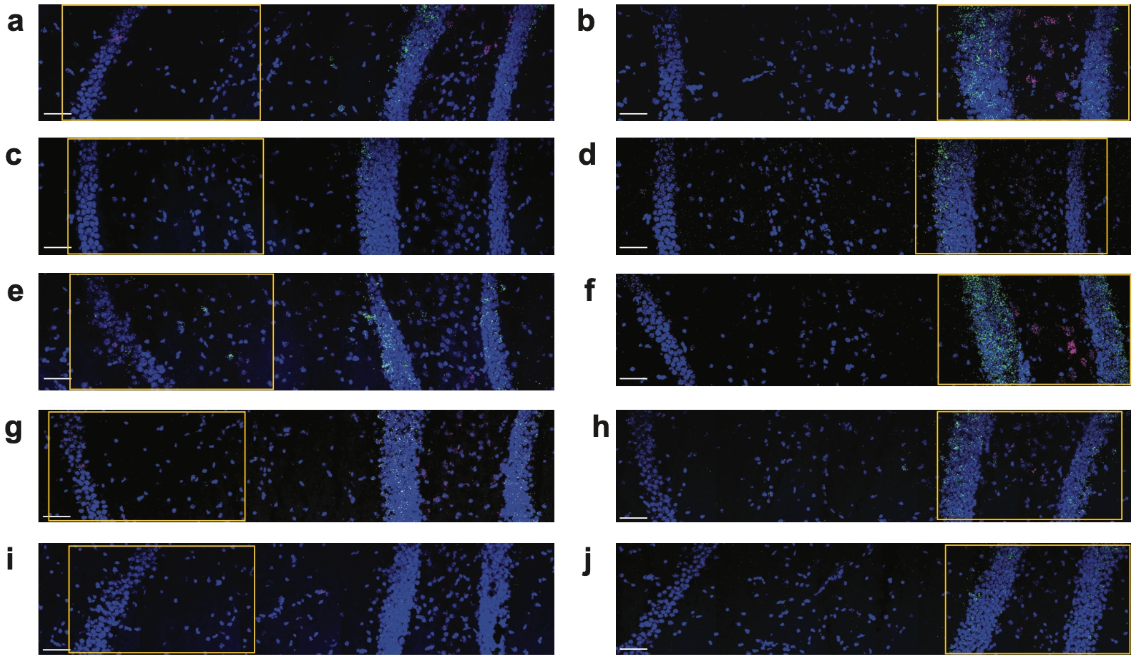

Supplementary Figure 2. Uncropped RNAscope images related to Figures 2 and 4. Full tiled RNAscope images related to **(a)** Fig 2i (NP CA1), **(b)** Fig 2j (NP DG), **(c)** Fig 2i (Control RE CA1), **(d)** Fig 2j (Control RE DG), **(e)** Fig 2i (Stress RE CA1), **(f)** Fig 2j (Stress RE DG), **(g)** Fig 4g (NP-mCherry CA1), **(h)** Fig 4h (NP-mCherry DG), **(i)** Fig 4g (NP-hM4Di CA1), **(j)** Fig 4h (NP-hM4Di DG). Scale bars, 50 μm. Yellow boxes indicate representative images presented in the Figures.

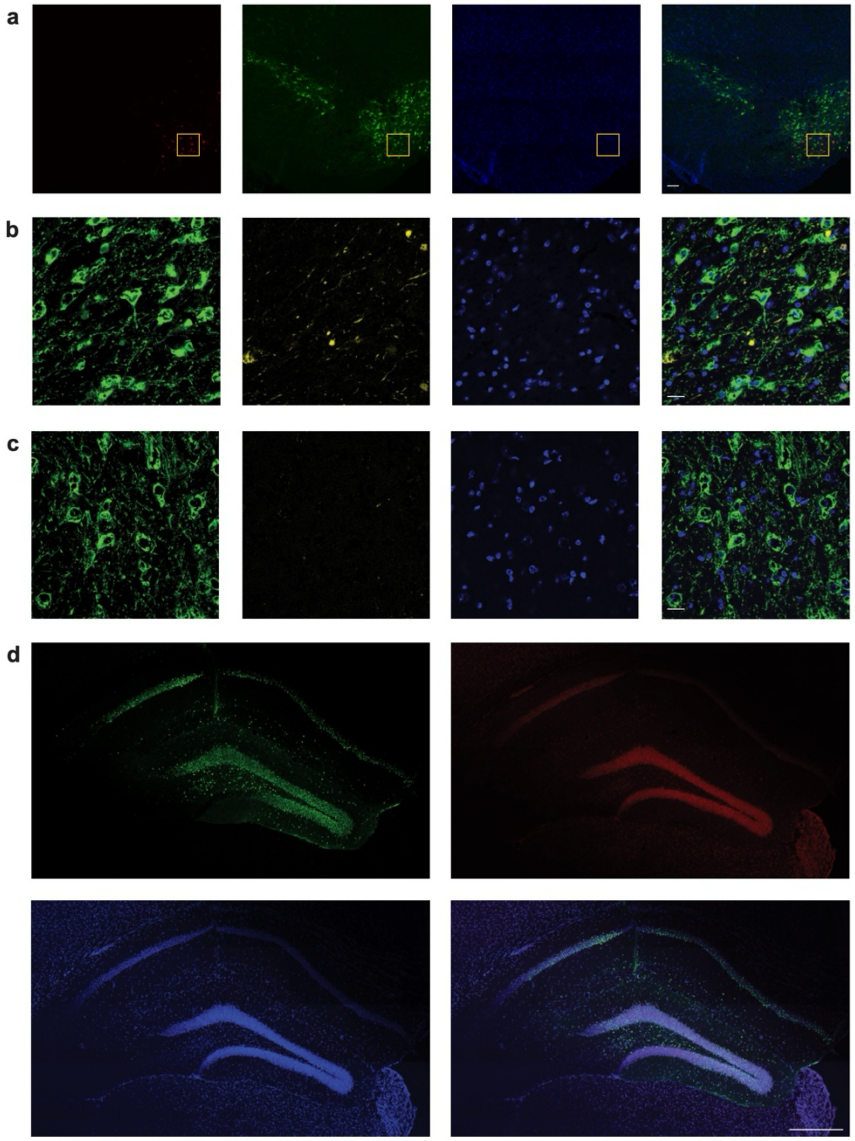

Supplementary Figure 3. Uncropped microscopy images related to Figures 4, 5 and Extended Data 9. Confocal images related to **(a)** Fig 4a-b; Scale bar, 100 µm, **(b)** Extended Data 9a (rTH-mCherry); Scale bar, 20 µm, **(c)** Extended Data 9a (rTH-hM4Di); Scale bar, 20 µm, **(d)** Fig 5b; Scale bar, 400 µm. Yellow boxes indicate representative images presented in the Figures.

Legends for Supplementary Tables 1-19

Supplementary Table 1. DESeq2 results for bulk RNA-seq of 11 brain regions, comparing NP vs. RE related to Figure 1 and Extended Data Figure 1.

Supplementary Table 2. DESeq2 results for bulk RNA-seq of 11 brain regions, adjusted for cell composition, comparing NP vs. RE related to Figure 1 and Extended Data Figure 1.

Supplementary Table 3. DESeq2 results for bulk RNA-seq of dHF across reproductive events (NP, Mating + No Pregnancy, Mating + Pregnancy + Birth, Pup Sensitized, RE) related to Extended Data Figure 3.

Supplementary Table 4. DESeq2 results for bulk RNA-seq of dHF across reproductive timepoints (7.5dpc, 17.5dpc, 2dpp, 21dpp, NP, RE) related to Extended Data Figure 4.

Supplementary Table 5. DESeq2 results for bulk RNA-seq of vHF across reproductive timepoints (7.5dpc, 17.5dpc, 2dpp, 21dpp, NP, RE) related to Extended Data Figure 4.

Supplementary Table 6. DESeq2 results for bulk RNA-seq of mPFC across reproductive timepoints (7.5dpc, 17.5dpc, 2dpp, 21dpp, NP, RE) related to Extended Data Figure 4.

Supplementary Table 7. DESeq2 results for bulk RNA-seq in dHF examining effect of late postpartum stress (NP, Control RE, Stress RE) related to Figure 2 and Extended Data Figure 5.

Supplementary Table 8. DESeq2 results for bulk RNA-seq in dHF examining effect of early postpartum stress (NP, Control RE, Early Stress RE) related to Extended Data Figure 6.

Supplementary Table 9. Diffbind results for H3K4me3Q5dop CUT&RUN-seq in dHF examing effect of parity (Control RE vs. NP) related to Figure 3 and Extended Data Figure 8.

Supplementary Table 10. Diffbind results for H3K4me3Q5dop CUT&RUN-seq in dHF examing effect of postpartum stress (Control RE vs. Stress RE) related to Figure 3 and Extended Data Figure 8.

Supplementary Table 11. Clinical, demographic, and parity information for donors of human postmortem dorsal subiculum tissues.

Supplementary Table 12. DESeq2 results for bulk RNA-seq in human dorsal subiculum examing effect of parity (Parous vs. NP) related to Figure 3 and Extended Data Figure 8.

Supplementary Table 13. Diffbind results for H3K4me3Q5dop CUT&RUN-seq in human dorsal subiculum examing effect of parity (Parous vs. NP) related to Figure 3 and Extended

Data Figure 8.

Supplementary Table 14. DESeq2 results for bulk RNA-seq in dHF examining effect of chronic chemogenetic suppression of VTA-dHF projection (NP-mCherry, NP-hM4Di, RE-mCherry, RE-hM4Di) related to Figure 4 and Extended Data Figure 9.

Supplementary Table 15. Diffbind results for H3K4me3Q5dop CUT&RUN-seq in dHF examining effect of chronic chemogenetic suppression of VTA-dHF projection (NP-mCherry vs. NP-hM4Di) related to Figure 4 and Extended Data Figure 9.

Supplementary Table 16. Diffbind results for H3K4me3Q5dop CUT&RUN-seq examing effect of postpartum stress in virally manipulated dHF (Control RE H3.3WT vs. Stress RE H3.3WT) related to Figure 5 and Extended Data Figure 10.

Supplementary Table 17. Diffbind results for H3K4me3Q5dop CUT&RUN-seq examing effect of H3.3Q5A in postpartum stress dHF (Stress RE H3.3WT vs. Stress RE H3.3Q5A) related to Figure 5 and Extended Data Figure 10.

Supplementary Table 18. DESeq2 results for bulk RNA-seq in dHF examining effect of H3.3Q5A in postpartum stress (Control RE H3.3WT, Stress RE H3.3WT, Stress RE H3.3Q5A) related to Figure 5 and Extended Data Figure 10.

Supplementary Table 19. Fisher's exact test ($p > 0.05$) results for estrous stage distributions across groups for all behavioral assays. All staging information is available in the Source Data file.
