# Supplementary material for: Dopamine drives persistent remodelling of the maternal brain
Source: Nature. 2026 May 20;654(8118):465–75. doi: 10.1038/s41586-026-10509-4 (PMC13253353; doi:10.1038/s41586-026-10509-4)
Supplement: Supplementary file 2 — Reporting Summary [file 41586_2026_10509_MOESM2_ESM.pdf]

Corresponding author(s): Ian Maze, PhD

Last updated by author(s): April 2, 2026

Reporting Summary

Nature Portfolio wishes to improve the reproducibility of the work that we publish. This form provides structure for consistency and transparency in reporting. For further information on Nature Portfolio policies, see our Editorial Policies and the Editorial Policy Checklist.

Statistics

For all statistical analyses, confirm that the following items are present in the figure legend, table legend, main text, or Methods section.

n/a	Confirmed	
☐	☒	The exact sample size (*n*) for each experimental group/condition, given as a discrete number and unit of measurement
☐	☒	A statement on whether measurements were taken from distinct samples or whether the same sample was measured repeatedly
☐	☒	The statistical test(s) used AND whether they are one- or two-sided<br>*Only common tests should be described solely by name; describe more complex techniques in the Methods section.*
☐	☒	A description of all covariates tested
☐	☒	A description of any assumptions or corrections, such as tests of normality and adjustment for multiple comparisons
☐	☒	A full description of the statistical parameters including central tendency (e.g. means) or other basic estimates (e.g. regression coefficient) AND variation (e.g. standard deviation) or associated estimates of uncertainty (e.g. confidence intervals)
☐	☒	For null hypothesis testing, the test statistic (e.g. *F*, *t*, *r*) with confidence intervals, effect sizes, degrees of freedom and *P* value noted<br>*Give P values as exact values whenever suitable.*
☒	☐	For Bayesian analysis, information on the choice of priors and Markov chain Monte Carlo settings
☐	☒	For hierarchical and complex designs, identification of the appropriate level for tests and full reporting of outcomes
☐	☒	Estimates of effect sizes (e.g. Cohen's *d*, Pearson's *r*), indicating how they were calculated

Our web collection on statistics for biologists contains articles on many of the points above.

Software and code

Policy information about availability of computer code

Data collection	Data were analysed using publicly available software and standard pipelines, as described in the Methods. No custom code was generated for this study; however, any related analysis scripts or details are available from the corresponding author upon reasonable request.
Data analysis	Bulk RNA-seq analysis: Raw fastq files, containing an average of 20–30 million reads per sample, were processed for pseudoalignment and abundance quantification using Kallisto (v. 0.46.1) against the Ensembl Mus musculus reference (v. 79). To filter lowly-expressed genes, only those with a total read count of at least 10 across all samples were retained. To account for unwanted variation among samples within each sequencing experiment that could arise from technical or biological factors unrelated to the conditions of interest (including litter size, estrous stage, day of sample collection, etc.), RUVs (v1.32.0) was applied with a negative control gene set derived from the total genes identified per sequencing experiment, after ensuring that unwanted variation did not correlate with covariates of interest, as described previously. Differential expression analysis was performed using DESeq2 (v1.38.3), with significant genes defined by an adjusted p-value < 0.05. For brain-wide transcriptome comparisons in which samples were processed across multiple sequencing runs and stemming from separate cohorts, pairwise comparisons were performed independently for each brain region. For all other experiments, where subjects came from the same cohort and were processed in a single sequencing run, all groups were analyzed together to maintain consistent normalization within the experiment. Gene expression time course analyses examining the periods before, during, and after pregnancy and postpartum were performed on normalized count data using the ImpulseDE2 package (v0.99.10)20 for each brain region. Significant genes exhibiting transient regulation or monotonous changes in expression were identified using case-only differential expression analysis, with a Q-value threshold of 0.05. Enrichment analyses were conducted using Ingenuity Pathway Analysis (Qiagen, Inc., v.01-23-01), ShinyGO (v. 0.81), and EnrichR (ChEA database, accessed May 2025 and November 2025, reflecting analyses conducted at different stages of the study).<br><br>snRNA-seq analysis: FastQ files were processed with the 10X Genomics Cell Ranger pipeline (v7.1.0) to demultiplex reads, align them to the mouse genome (mm10-2020-A), remove PCR duplicates, and generate gene expression matrices. Cell Ranger filtered outputs were analyzed

using Seurat v4.3.029, and mitochondrial RNA content per cell was calculated using the GRCm39 (mm10) genome annotation and regressed out using SCTransform normalization protocol included in the Seurat toolkit with 20 principal components (PCs) and a resolution of 0.1. To estimate ambient RNA and correct for background contamination, the SoupX (v1.6.2) package was used for each sample using raw and filtered feature matrices from the Cell Ranger output. Heterotypic doublets were identified and removed using DoubletFinder (v2) to ensure the integrity of singlet datasets. Filtered singlet datasets were then re-normalized and integrated using the same Seurat SCTransform v2 workflow mentioned above. Cell clusters were annotated using a combination of expert curation based on published marker genes, and label transfer from hippocampal reference datasets, including the Allen Brain Map and Broad Institute resources. Clusters with contaminant cell populations expressing markers for choroid plexus (Ttr), ependymal (Tmem212), and vascular leptomeningeal cells (Vtn, Col1a2) were removed from the analysis. Additionally, as the sequential 2mm micropunches encompassed portions of cortical, thalamic and vHpF regions, clusters characterized by enrichment of published non-dHpF neuronal markers using Seurat's FindMarkers function (layer 5/6 cortical: Rorb, Foxp2; ventral granule neurons: Tox3) were also removed from the analysis. Cell cluster proportion analyses were conducted using the scProportionTest package (v1), which employs a Monte Carlo permutation test to evaluate whether observed differences result from random sampling. Proportional differences between conditions were compared to a null distribution generated by resampling, and statistical significance was determined by permutation-based p-values with confidence intervals estimated via bootstrapping. Differential expression analysis was conducted using pseudobulk analysis, where gene counts were summed across all cells within each sample for each cell type cluster using the AggregateExpression() function. DESeq2 was then applied at the sample level to conduct differential expression. To explore pathways underlying cluster-specific differences across conditions, pathway analysis was conducted using ShinyGO on genes meeting the following criteria: log2FC > 1.5 and $p < 0.05$.

CUT&RUN-seq:Raw fastq files were aligned to the hg19 or mm10 genome using bowtie2 (v2.5.0). Low-quality reads were filtered using Samtools (v.1.9) with a MAPQ cut-off score of 30. Only unique, deduplicated reads were retained for further processing. Bigwig files were produced using the deepTools package (v.3.5.1), using an ENCODE hg19 or mm10 v2 blacklist file to discard regions with consistently non-specific signal, and scaled using E. coli spike-in controls to normalize sequencing depth. To determine normalization factors based on E. coli reads, each sample was aligned to the E. coli genome (MG1655), and the unique deduplicated reads were compared across groups per antibody per experiment. The sample with the lowest number of E. coli reads was determined ("minimum"), and all samples were scaled by dividing their corresponding E. coli read count by this minimum number. For each group, bigwig files were merged and peak callling was conducted using MACS2 (v2.1.0) with the corresponding merged IgG file as control, filtered for peaks with FDR < 0.05. Peak annotation was conducted using HOMER (v4.1.1). Heatmaps were made either using the DiffBind (v3.8.4) or deepTools (v3.5.5) packages. For deepTools, heatmaps were made by merging DEGs from RNA-seq data with TSSs downloaded from the UCSC genome browser using the canonically annotated transcript for each gene. Profiles were generated and statistically analyzed using the deepStats package108 by using the dsCompareCurves function to perform Wilcoxon Rank-sum tests per-bin. For DiffBind analysis, heatmaps were made for peaks identified by DiffBind's differential peak algorithm, where differential peaks were first filtered using a log2(fold change) threshold > 0.1 and defined at $p < 0.05$, where log2(fold change) was calculated as log2(parity) − log2(NP), based on prior empirical observations used to define thresholds for differential peaks. ChEA analysis on annotated loci was conducted using EnrichR with a significance threshold of adjusted $p < 0.05$.

Image analysis: Subcellular quantification of individual puncta per 100µm² nucleus in RNAscope images, identified by nuclear detection using DAPI staining, was performed on maximum intensity projections using QuPath (v0.5.1). Microscopy images for immunoreactivity were analyzed using FIJI (v2.14.0/1.54f).

Behavioral analysis: ANY-maze (v. 7.51), Ethovision (XT 11) and Fusion (v.5.6) software were used to acquire behavioral tracking data. Statistical analyses for behavioral were conducted using Prism software (GraphPad, v.10.4.1).

For manuscripts utilizing custom algorithms or software that are central to the research but not yet described in published literature, software must be made available to editors and reviewers. We strongly encourage code deposition in a community repository (e.g. GitHub). See the Nature Portfolio guidelines for submitting code & software for further information.

Data

Policy information about availability of data

All manuscripts must include a data availability statement. This statement should provide the following information, where applicable:

- Accession codes, unique identifiers, or web links for publicly available datasets
- A description of any restrictions on data availability
- For clinical datasets or third party data, please ensure that the statement adheres to our policy

The genomics data generated in this study have been deposited in the National Center for Biotechnology Information Gene Expression Omnibus (GEO) database under the SuperSeries GSE298544. We declare that the data supporting findings for this study are available within the article and Supplementary Information. Reference genomes used in this study include the mouse genome mm10 and human genome hg19. Publicly available reference datasets used for annotation included resources from the Allen Brain Map and Broad Institute, with gene annotations obtained from the UCSC Genome Browser and ENCODE blacklist regions applied where appropriate. All data supporting the findings of this study are available within the article and Supplementary Information. Related data, including raw microscopy images, are available from the corresponding author upon reasonable request.

Research involving human participants, their data, or biological material

Policy information about studies with human participants or human data. See also policy information about sex, gender (identity/presentation), and sexual orientation and race, ethnicity and racism.

Reporting on sex and gender	Yes
Reporting on race, ethnicity, or other socially relevant groupings	Race and ethnicity information not available

Population characteristics	Covariate-relevant population characteristics for human subjects, including age, sex, parity (pregnancies and live births), menopausal status, hormone use, and psychiatric history, are provided in Supplementary Table 11. All subjects were female, with ages ranging from 23–68 years and 0–2 pregnancies/live births. Menopausal status and hormone use were also documented where available. No genotypic information is available for these samples.
Recruitment	Yes
Ethics oversight	Brain tissues used in this study were provided by the Douglas Brain Bank (RRID:SCR_025991) with ethical approval from the Research Ethics Board (REB) of the Centre intégré universitaire de santé et de services sociaux (CIUSSS) de l'Ouest-de-l'Île-de-Montréal.

Note that full information on the approval of the study protocol must also be provided in the manuscript.

Field-specific reporting

Please select the one below that is the best fit for your research. If you are not sure, read the appropriate sections before making your selection.

☒ Life sciences ☐ Behavioural & social sciences ☐ Ecological, evolutionary & environmental sciences

For a reference copy of the document with all sections, see nature.com/documents/nr-reporting-summary-flat.pdf

Life sciences study design

All studies must disclose on these points even when the disclosure is negative.

Sample size	Sample sizes were determined based on variability observed in prior experiments using these behavioral, molecular, and sequencing endpoints, as well as prior experience with these assays. No statistical methods were used to predetermine sample size. Sample sizes were selected to detect biologically meaningful differences with a confidence level of 95% and are consistent with those commonly used in the field. Experimental group sizes were balanced where possible, and all experiments included independent biological replicates. Practical considerations, including animal availability, variability in pregnancy success and litter viability, surgical survival rates, viral expression efficiency and targeting accuracy, and overall experimental feasibility, were also taken into account when determining sample sizes.
Data exclusions	Grubb's test (alpha = 0.05) was applied to detect outliers where necessary.
Replication	All biological endpoints were reliably reproduced using numerous biological (>3 for all experiments in which statistics were employed) and technical replicates for each experiment.
Randomization	For all experiments, animals were randomly assigned to experimental groups, including viral treatment and stress conditions. Animals were also randomly selected for breeding to generate parity-defined groups. Randomization was performed across litters where possible to minimize potential litter effects. All animals were housed under similar conditions, and experiments were conducted using age-matched cohorts where feasible to minimize potential confounding variables.
Blinding	Where feasible, data collection and quantification (including behavioral scoring, microscopy analyses, and molecular quantification) were performed with investigators blinded to group allocation. For experiments involving pregnancy, postpartum, and stress manipulations, blinding during data collection was not possible due to the nature of the experimental design (for example, the presence of pups, differences in pregnancy status, and administration of the stress protocol). Importantly, investigators were blinded to group allocation during data analysis for all experiments.

Behavioural & social sciences study design

All studies must disclose on these points even when the disclosure is negative.

Study description	*Briefly describe the study type including whether data are quantitative, qualitative, or mixed-methods (e.g. qualitative cross-sectional, quantitative experimental, mixed-methods case study).*
Research sample	*State the research sample (e.g. Harvard university undergraduates, villagers in rural India) and provide relevant demographic information (e.g. age, sex) and indicate whether the sample is representative. Provide a rationale for the study sample chosen. For studies involving existing datasets, please describe the dataset and source.*
Sampling strategy	*Describe the sampling procedure (e.g. random, snowball, stratified, convenience). Describe the statistical methods that were used to predetermine sample size OR if no sample-size calculation was performed, describe how sample sizes were chosen and provide a rationale for why these sample sizes are sufficient. For qualitative data, please indicate whether data saturation was considered, and what criteria were used to decide that no further sampling was needed.*
Data collection	*Provide details about the data collection procedure, including the instruments or devices used to record the data (e.g. pen and paper, computer, eye tracker, video or audio equipment) whether anyone was present besides the participant(s) and the researcher, and whether the researcher was blind to experimental condition and/or the study hypothesis during data collection.*
Timing	*Indicate the start and stop dates of data collection. If there is a gap between collection periods, state the dates for each sample cohort.*

Data exclusions	*If no data were excluded from the analyses, state so OR if data were excluded, provide the exact number of exclusions and the rationale behind them, indicating whether exclusion criteria were pre-established.*
Non-participation	*State how many participants dropped out/declined participation and the reason(s) given OR provide response rate OR state that no participants dropped out/declined participation.*
Randomization	*If participants were not allocated into experimental groups, state so OR describe how participants were allocated to groups, and if allocation was not random, describe how covariates were controlled.*

Ecological, evolutionary & environmental sciences study design

All studies must disclose on these points even when the disclosure is negative.

Study description	*Briefly describe the study. For quantitative data include treatment factors and interactions, design structure (e.g. factorial, nested, hierarchical), nature and number of experimental units and replicates.*
Research sample	*Describe the research sample (e.g. a group of tagged Passer domesticus, all Stenocereus thurberi within Organ Pipe Cactus National Monument), and provide a rationale for the sample choice. When relevant, describe the organism taxa, source, sex, age range and any manipulations. State what population the sample is meant to represent when applicable. For studies involving existing datasets, describe the data and its source.*
Sampling strategy	*Note the sampling procedure. Describe the statistical methods that were used to predetermine sample size OR if no sample-size calculation was performed, describe how sample sizes were chosen and provide a rationale for why these sample sizes are sufficient.*
Data collection	*Describe the data collection procedure, including who recorded the data and how.*
Timing and spatial scale	*Indicate the start and stop dates of data collection, noting the frequency and periodicity of sampling and providing a rationale for these choices. If there is a gap between collection periods, state the dates for each sample cohort. Specify the spatial scale from which the data are taken*
Data exclusions	*If no data were excluded from the analyses, state so OR if data were excluded, describe the exclusions and the rationale behind them, indicating whether exclusion criteria were pre-established.*
Reproducibility	*Describe the measures taken to verify the reproducibility of experimental findings. For each experiment, note whether any attempts to repeat the experiment failed OR state that all attempts to repeat the experiment were successful.*
Randomization	*Describe how samples/organisms/participants were allocated into groups. If allocation was not random, describe how covariates were controlled. If this is not relevant to your study, explain why.*
Blinding	*Describe the extent of blinding used during data acquisition and analysis. If blinding was not possible, describe why OR explain why blinding was not relevant to your study.*

Did the study involve field work? ☐ Yes ☐ No

Field work, collection and transport

Field conditions	*Describe the study conditions for field work, providing relevant parameters (e.g. temperature, rainfall).*
Location	*State the location of the sampling or experiment, providing relevant parameters (e.g. latitude and longitude, elevation, water depth).*
Access & import/export	*Describe the efforts you have made to access habitats and to collect and import/export your samples in a responsible manner and in compliance with local, national and international laws, noting any permits that were obtained (give the name of the issuing authority, the date of issue, and any identifying information).*
Disturbance	*Describe any disturbance caused by the study and how it was minimized.*

Reporting for specific materials, systems and methods

We require information from authors about some types of materials, experimental systems and methods used in many studies. Here, indicate whether each material, system or method listed is relevant to your study. If you are not sure if a list item applies to your research, read the appropriate section before selecting a response.

Materials & experimental systems

n/a	Involved in the study
☐	☒ Antibodies
☐	☒ Eukaryotic cell lines
☒	☐ Palaeontology and archaeology
☐	☒ Animals and other organisms
☒	☐ Clinical data
☒	☐ Dual use research of concern
☒	☐ Plants

Methods

n/a	Involved in the study
☐	☒ ChIP-seq
☒	☐ Flow cytometry
☒	☐ MRI-based neuroimaging

Antibodies

Antibodies used

H3K4me3 (Active Motif, 39159), H3K4me3Q5dopaminyl (Millipore, ABE2590), or rabbit IgG (Invitrogen, 10500c), mouse anti-HA (1:1000, Abcam #ab18181), chicken anti-tyrosine hydroxylase (1:500, Aves Labs #TYH), rabbit anti-Fos (1:2000, Synaptic Systems #226-008), Mouse anti-TG2 (Abcam 2386), Goat anti-Mouse IgG (Invitrogen, A-21235)

Validation

Primary antibodies used in this study were validated for the appropriate species either by the manufacturer (as indicated in datasheets and validation data provided on the manufacturer's website) or through prior publications cited in the manuscript. Where applicable, antibodies were additionally validated in-house using immunoblotting, immunoprecipitation, or immunofluorescence/immunohistochemistry in relevant tissues or cell types prior to experimentation.

Eukaryotic cell lines

Policy information about cell lines and Sex and Gender in Research

Cell line source(s)

HeLa (CRM-CCL-2) cell lines were obtained from the American Type Culture Collection (ATCC).

Authentication

Human tissue culture cell lines (HeLa) were imaged for appropriate morphology.

Mycoplasma contamination

We can confirm that all cell lines tested negative for mycoplasma contamination.

Commonly misidentified lines
(See ICLAC register)

No commonly misidentified cell lines were used.

Palaeontology and Archaeology

Specimen provenance

Provide provenance information for specimens and describe permits that were obtained for the work (including the name of the issuing authority, the date of issue, and any identifying information). Permits should encompass collection and, where applicable, export.

Specimen deposition

Indicate where the specimens have been deposited to permit free access by other researchers.

Dating methods

If new dates are provided, describe how they were obtained (e.g. collection, storage, sample pretreatment and measurement), where they were obtained (i.e. lab name), the calibration program and the protocol for quality assurance OR state that no new dates are provided.

☐ Tick this box to confirm that the raw and calibrated dates are available in the paper or in Supplementary Information.

Ethics oversight

Identify the organization(s) that approved or provided guidance on the study protocol, OR state that no ethical approval or guidance was required and explain why not.

Note that full information on the approval of the study protocol must also be provided in the manuscript.

Animals and other research organisms

Policy information about studies involving animals; ARRIVE guidelines recommended for reporting animal research, and Sex and Gender in Research

Laboratory animals

Wild-type C57BL/6J mice were purchased from The Jackson Laboratory at 8 weeks of age and maintained on a 12-h/12-h light/dark cycle throughout the experiments. Mice were housed in temperature- and humidity-controlled conditions (approximately 22±2°C and 50 ±10% humidity) with ad libitum access to food and water. All behavioral testing occurred during the animals' light cycle. Behavioral testing was conducted in adult mice aged 18–30 weeks. Pups used for pup retrieval experiments were aged 4-6 dpp, and pups used for maternal separation stress experiments were aged postnatal day 10–20 dpp.

Wild animals

No wild animals were used in this study.

Reporting on sex	Due to the nature of the study, all mice were female.
Field-collected samples	No field collected samples were used in the study.
Ethics oversight	All animal protocols were approved by the IACUC at both the Icahn School of Medicine at Mount Sinai (ISMMS).

Note that full information on the approval of the study protocol must also be provided in the manuscript.

Clinical data

Policy information about clinical studies

All manuscripts should comply with the ICMJE guidelines for publication of clinical research and a completed CONSORT checklist must be included with all submissions.

Clinical trial registration	*Provide the trial registration number from ClinicalTrials.gov or an equivalent agency.*
Study protocol	*Note where the full trial protocol can be accessed OR if not available, explain why.*
Data collection	*Describe the settings and locales of data collection, noting the time periods of recruitment and data collection.*
Outcomes	*Describe how you pre-defined primary and secondary outcome measures and how you assessed these measures.*

Dual use research of concern

Policy information about dual use research of concern

Hazards

Could the accidental, deliberate or reckless misuse of agents or technologies generated in the work, or the application of information presented in the manuscript, pose a threat to:

No	Yes	
☒	☐	Public health
☒	☐	National security
☒	☐	Crops and/or livestock
☒	☐	Ecosystems
☒	☐	Any other significant area

Experiments of concern

Does the work involve any of these experiments of concern:

No	Yes	
☒	☐	Demonstrate how to render a vaccine ineffective
☒	☐	Confer resistance to therapeutically useful antibiotics or antiviral agents
☒	☐	Enhance the virulence of a pathogen or render a nonpathogen virulent
☒	☐	Increase transmissibility of a pathogen
☒	☐	Alter the host range of a pathogen
☒	☐	Enable evasion of diagnostic/detection modalities
☒	☐	Enable the weaponization of a biological agent or toxin
☒	☐	Any other potentially harmful combination of experiments and agents

Plants

Seed stocks

Report on the source of all seed stocks or other plant material used. If applicable, state the seed stock centre and catalogue number. If plant specimens were collected from the field, describe the collection location, date and sampling procedures.

Novel plant genotypes

Describe the methods by which all novel plant genotypes were produced. This includes those generated by transgenic approaches, gene editing, chemical/radiation-based mutagenesis and hybridization. For transgenic lines, describe the transformation method, the number of independent lines analyzed and the generation upon which experiments were performed. For gene-edited lines, describe the editor used, the endogenous sequence targeted for editing, the targeting guide RNA sequence (if applicable) and how the editor was applied.

Authentication

Describe any authentication procedures for each seed stock used or novel genotype generated. Describe any experiments used to assess the effect of a mutation and, where applicable, how potential secondary effects (e.g. second site T-DNA insertions, mosiacism, off-target gene editing) were examined.

ChIP-seq

Data deposition

☒ Confirm that both raw and final processed data have been deposited in a public database such as GEO.

☒ Confirm that you have deposited or provided access to graph files (e.g. BED files) for the called peaks.

Data access links
May remain private before publication.

The CUT&RUN-seq data generated in this study have been deposited in the National Center for Biotechnology Information Gene Expression Omnibus (GEO) database under accession number GSE298544. This includes the raw fastq files for each experiment, as well as bigwigs made from individual and merged BAM files. These bigwigs were scaled according to an E. coli spike-in DNA for each CUT&RUN reaction, with scaling occurring for each treatment group for the same antibody. See methods for details.

Files in database submission

processed data file *raw file	raw file	
AB1_192_rmdup.scaled.bw	192_Ab1_S61_L007_R1_001.fastq.gz	192_Ab1_S61_L007_R2_001.fastq.gz
AB1_194_rmdup.scaled.bw	194_Ab1_S64_L007_R1_001.fastq.gz	194_Ab1_S64_L007_R2_001.fastq.gz
AB1_198_rmdup.scaled.bw	198_Ab1_S70_L007_R1_001.fastq.gz	198_Ab1_S70_L007_R2_001.fastq.gz
AB1_206_rmdup.scaled.bw	206_Ab1_S76_L007_R1_001.fastq.gz	206_Ab1_S76_L007_R2_001.fastq.gz
AB1_196_rmdup.scaled.bw	196_Ab1_S67_L007_R1_001.fastq.gz	196_Ab1_S67_L007_R2_001.fastq.gz
AB1_203_rmdup.scaled.bw	203_Ab1_S73_L007_R1_001.fastq.gz	203_Ab1_S73_L007_R2_001.fastq.gz
AB1_210_rmdup.scaled.bw	210_Ab1_S79_L007_R1_001.fastq.gz	210_Ab1_S79_L007_R2_001.fastq.gz
AB1_213_rmdup.scaled.bw	213_Ab1_S82_L007_R1_001.fastq.gz	213_Ab1_S82_L007_R2_001.fastq.gz
AB1_NP10_rmdup.scaled.bw	NP10_Ab1_S88_L007_R1_001.fastq.gz	NP10_Ab1_S88_L007_R2_001.fastq.gz
AB1_NP11_rmdup.scaled.bw	NP11_Ab1_S91_L007_R1_001.fastq.gz	NP11_Ab1_S91_L007_R2_001.fastq.gz
AB1_NP12_rmdup.scaled.bw	NP12_Ab1_S94_L007_R1_001.fastq.gz	NP12_Ab1_S94_L007_R2_001.fastq.gz
AB1_NP9_rmdup.scaled.bw	NP9_Ab1_S85_L007_R1_001.fastq.gz	NP9_Ab1_S85_L007_R2_001.fastq.gz
AB3_192_rmdup.scaled.bw	192_Ab3_S63_L007_R1_001.fastq.gz	192_Ab3_S63_L007_R2_001.fastq.gz
AB3_194_rmdup.scaled.bw	194_Ab3_S66_L007_R1_001.fastq.gz	194_Ab3_S66_L007_R2_001.fastq.gz
AB3_198_rmdup.scaled.bw	198_Ab3_S72_L007_R1_001.fastq.gz	198_Ab3_S72_L007_R2_001.fastq.gz
AB3_206_rmdup.scaled.bw	206_Ab3_S78_L007_R1_001.fastq.gz	206_Ab3_S78_L007_R2_001.fastq.gz
AB3_196_rmdup.scaled.bw	196_Ab3_S69_L007_R1_001.fastq.gz	196_Ab3_S69_L007_R2_001.fastq.gz
AB3_203_rmdup.scaled.bw	203_Ab3_S75_L007_R1_001.fastq.gz	203_Ab3_S75_L007_R2_001.fastq.gz
AB3_210_rmdup.scaled.bw	210_Ab3_S81_L007_R1_001.fastq.gz	210_Ab3_S81_L007_R2_001.fastq.gz
AB3_213_rmdup.scaled.bw	213_Ab3_S84_L007_R1_001.fastq.gz	213_Ab3_S84_L007_R2_001.fastq.gz
AB3_NP10_rmdup.scaled.bw	NP10_Ab3_S90_L007_R1_001.fastq.gz	NP10_Ab3_S90_L007_R2_001.fastq.gz
AB3_NP11_rmdup.scaled.bw	NP11_Ab3_S93_L007_R1_001.fastq.gz	NP11_Ab3_S93_L007_R2_001.fastq.gz
AB3_NP12_rmdup.scaled.bw	NP12_Ab3_S96_L007_R1_001.fastq.gz	NP12_Ab3_S96_L007_R2_001.fastq.gz
AB3_NP9_rmdup.scaled.bw	NP9_Ab3_S87_L007_R1_001.fastq.gz	NP9_Ab3_S87_L007_R2_001.fastq.gz
AB1_755_rmdup.scaled.bw	755_Ab1_S37_L006_R1_001.fastq.gz	755_Ab1_S37_L006_R2_001.fastq.gz
AB1_757_rmdup.scaled.bw	757_Ab1_S40_L006_R1_001.fastq.gz	757_Ab1_S40_L006_R2_001.fastq.gz
AB1_760_rmdup.scaled.bw	760_Ab1_S43_L006_R1_001.fastq.gz	760_Ab1_S43_L006_R2_001.fastq.gz
AB1_767_rmdup.scaled.bw	767_Ab1_S46_L006_R1_001.fastq.gz	767_Ab1_S46_L006_R2_001.fastq.gz
AB1_768_rmdup.scaled.bw	768_Ab1_S49_L006_R1_001.fastq.gz	768_Ab1_S49_L006_R2_001.fastq.gz
AB1_774_rmdup.scaled.bw	774_Ab1_S52_L006_R1_001.fastq.gz	774_Ab1_S52_L006_R2_001.fastq.gz
AB1_776_rmdup.scaled.bw	776_Ab1_S55_L006_R1_001.fastq.gz	776_Ab1_S55_L006_R2_001.fastq.gz
AB1_777_rmdup.scaled.bw	777_Ab1_S58_L006_R1_001.fastq.gz	777_Ab1_S58_L006_R2_001.fastq.gz
AB3_755_rmdup.scaled.bw	755_Ab3_S39_L006_R1_001.fastq.gz	755_Ab3_S39_L006_R2_001.fastq.gz
AB3_757_rmdup.scaled.bw	757_Ab3_S42_L006_R1_001.fastq.gz	757_Ab3_S42_L006_R2_001.fastq.gz
AB3_760_rmdup.scaled.bw	760_Ab3_S45_L006_R1_001.fastq.gz	760_Ab3_S45_L006_R2_001.fastq.gz
AB3_767_rmdup.scaled.bw	767_Ab3_S48_L006_R1_001.fastq.gz	767_Ab3_S48_L006_R2_001.fastq.gz
AB3_768_rmdup.scaled.bw	768_Ab3_S51_L006_R1_001.fastq.gz	768_Ab3_S51_L006_R2_001.fastq.gz
AB3_774_rmdup.scaled.bw	774_Ab3_S54_L006_R1_001.fastq.gz	774_Ab3_S54_L006_R2_001.fastq.gz
AB3_776_rmdup.scaled.bw	776_Ab3_S57_L006_R1_001.fastq.gz	776_Ab3_S57_L006_R2_001.fastq.gz
AB3_777_rmdup.scaled.bw	777_Ab3_S60_L006_R1_001.fastq.gz	777_Ab3_S60_L006_R2_001.fastq.gz
AB1_258_rmdup.scaled_fixed.bw	AB1_258_S289_L008_R1_001_fixed.fastq.gz	AB1_258_S289_L008_R2_001_fixed.fastq.gz
AB1_442_rmdup.scaled.bw	AB1_442_S294_L008_R1_001.fastq.gz	AB1_442_S294_L008_R2_001.fastq.gz
AB1_461_rmdup.scaled.bw	AB1_461_S297_L008_R1_001.fastq.gz	AB1_461_S297_L008_R2_001.fastq.gz

AB1_484_rmdup.scaled.bw AB1_484_S300_L008_R1_001.fastq.gz AB1_484_S300_L008_R2_001.fastq.gz
AB1_505_rmdup.scaled.bw AB1_505_S303_L008_R1_001.fastq.gz AB1_505_S303_L008_R2_001.fastq.gz
AB1_532_rmdup.scaled.bw AB1_532_S309_L008_R1_001.fastq.gz AB1_532_S309_L008_R2_001.fastq.gz
AB1_560_rmdup.scaled.bw AB1_560_S312_L008_R1_001.fastq.gz AB1_560_S312_L008_R2_001.fastq.gz
AB1_616_rmdup.scaled.bw AB1_616_S315_L008_R1_001.fastq.gz AB1_616_S315_L008_R2_001.fastq.gz
AB3_258_rmdup.scaled.bw AB3_258_S290_L008_R1_001.fastq.gz AB3_258_S290_L008_R2_001.fastq.gz
AB3_442_rmdup.scaled.bw AB3_442_S296_L008_R1_001.fastq.gz AB3_442_S296_L008_R2_001.fastq.gz
AB3_461_rmdup.scaled.bw AB3_461_S299_L008_R1_001.fastq.gz AB3_461_S299_L008_R2_001.fastq.gz
AB3_484_rmdup.scaled.bw AB3_484_S302_L008_R1_001.fastq.gz AB3_484_S302_L008_R2_001.fastq.gz
AB3_505_rmdup.scaled.bw AB3_505_S305_L008_R1_001.fastq.gz AB3_505_S305_L008_R2_001.fastq.gz
AB3_532_rmdup.scaled.bw AB3_532_S311_L008_R1_001.fastq.gz AB3_532_S311_L008_R2_001.fastq.gz
AB3_560_rmdup.scaled.bw AB3_560_S314_L008_R1_001.fastq.gz AB3_560_S314_L008_R2_001.fastq.gz
AB3_616_rmdup.scaled.bw AB3_616_S317_L008_R1_001.fastq.gz AB3_616_S317_L008_R2_001.fastq.gz
H_DualDopeWT_merged.scaled.bs10.bw H_1_DD_WT_S12_L005_R1_001.fastq.gz
H_1_DD_WT_S12_L005_R2_001.fastq.gz
H_DualDopeWT_merged.scaled.bs10.bw H_2_DD_WT_S13_L005_R1_001.fastq.gz
H_2_DD_WT_S13_L005_R2_001.fastq.gz
H_DualDopeWT_merged.scaled.bs10.bw H_3_DD_WT_S14_L005_R1_001.fastq.gz
H_3_DD_WT_S14_L005_R2_001.fastq.gz
H_1_IgG_WT_S18_L005.hg19.sorted_uniq.RPKM.bs10.sl30.bw H_1_IgG_WT_S18_L005_R1_001.fastq.gz
H_1_IgG_WT_S18_L005_R2_001.fastq.gz
H_K4me3WT_merged.scaled.bs10.bw H_1_K4_WT_S15_L005_R1_001.fastq.gz
H_1_K4_WT_S15_L005_R2_001.fastq.gz
H_K4me3WT_merged.scaled.bs10.bw H_2_K4_WT_S16_L005_R1_001.fastq.gz
H_2_K4_WT_S16_L005_R2_001.fastq.gz
H_K4me3WT_merged.scaled.bs10.bw H_3_K4_WT_S17_L005_R1_001.fastq.gz
H_3_K4_WT_S17_L005_R2_001.fastq.gz
H_DualDopeKO_merged.scaled.bs10.bw H_1_DD_KO_S19_L005_R1_001.fastq.gz
H_1_DD_KO_S19_L005_R2_001.fastq.gz
H_DualDopeKO_merged.scaled.bs10.bw H_2_DD_KO_S20_L005_R1_001.fastq.gz
H_2_DD_KO_S20_L005_R2_001.fastq.gz
H_DualDopeKO_merged.scaled.bs10.bw H_3_DD_KO_S21_L005_R1_001.fastq.gz
H_3_DD_KO_S21_L005_R2_001.fastq.gz
H_1_IgG_KO_S25_L005.hg19.sorted_uniq.RPKM.bs10.sl30.bw H_1_IgG_KO_S25_L005_R1_001.fastq.gz
H_1_IgG_KO_S25_L005_R2_001.fastq.gz
H_K4me3KO_merged.scaled.bs10.bw H_1_K4_KO_S22_L005_R1_001.fastq.gz
H_1_K4_KO_S22_L005_R2_001.fastq.gz
H_K4me3KO_merged.scaled.bs10.bw H_2_K4_KO_S23_L005_R1_001.fastq.gz
H_2_K4_KO_S23_L005_R2_001.fastq.gz
H_K4me3KO_merged.scaled.bs10.bw H_3_K4_KO_S24_L005_R1_001.fastq.gz
H_3_K4_KO_S24_L005_R2_001.fastq.gz
904_dop_rmdup.scaled.bw 904_dop_S103_L006_R1_001.fastq.gz 904_dop_S103_L006_R2_001.fastq.gz
914_dop_rmdup.scaled.bw 914_dop_S104_L006_R1_001.fastq.gz 914_dop_S104_L006_R2_001.fastq.gz
915_dop_rmdup.scaled.bw 915_dop_S105_L006_R1_001.fastq.gz 915_dop_S105_L006_R2_001.fastq.gz
921_dop_rmdup.scaled.bw 921_dop_S106_L006_R1_001.fastq.gz 921_dop_S106_L006_R2_001.fastq.gz
922_dop_rmdup.scaled.bw 922_dop_S107_L006_R1_001.fastq.gz 922_dop_S107_L006_R2_001.fastq.gz
923_dop_rmdup.scaled.bw 923_dop_S108_L006_R1_001.fastq.gz 923_dop_S108_L006_R2_001.fastq.gz
925_dop_rmdup.scaled.bw 925_dop_S109_L006_R1_001.fastq.gz 925_dop_S109_L006_R2_001.fastq.gz
927_dop_rmdup.scaled.bw 927_dop_S110_L006_R1_001.fastq.gz 927_dop_S110_L006_R2_001.fastq.gz
930_dop_rmdup.scaled.bw 930_dop_S111_L006_R1_001.fastq.gz 930_dop_S111_L006_R2_001.fastq.gz
932_dop_rmdup.scaled.bw 932_dop_S112_L006_R1_001.fastq.gz 932_dop_S112_L006_R2_001.fastq.gz
937_dop_rmdup.scaled.bw 937_dop_S113_L006_R1_001.fastq.gz 937_dop_S113_L006_R2_001.fastq.gz
939_dop_rmdup.scaled.bw 939_dop_S114_L006_R1_001.fastq.gz 939_dop_S114_L006_R2_001.fastq.gz
941_dop_rmdup.scaled.bw 941_dop_S115_L006_R1_001.fastq.gz 941_dop_S115_L006_R2_001.fastq.gz
945_dop_rmdup.scaled.bw 945_dop_S116_L006_R1_001.fastq.gz 945_dop_S116_L006_R2_001.fastq.gz
946_dop_rmdup.scaled.bw 946_dop_S117_L006_R1_001.fastq.gz 946_dop_S117_L006_R2_001.fastq.gz
Con_WT_igg_rmdup.scaled.bw Group1_igg_S118_L006_R1_001.fastq.gz Group1_igg_S118_L006_R2_001.fastq.gz
ELS_WT_igg_rmdup.scaled.bw Group2_igg_S119_L006_R1_001.fastq.gz Group2_igg_S119_L006_R2_001.fastq.gz
ELS_Q5A_igg_rmdup.scaled.bw Group3_igg_S120_L006_R1_001.fastq.gz Group3_igg_S120_L006_R2_001.fastq.gz

Genome browser session
(e.g. UCSC)

Not applicable; no genome browser session was created. All bigwigs were visualized on local IGV browser.

Methodology

Replicates

There were 3-5 independent biological replicates for each antibody and group, each coming from a biologically independent sample. These biological replicates were assessed for similarity visually in IGV and by comparing alignment rate, peak calling (between biological replicates), and heatmaps (using MACS2/Deeptools). All replicates had high concordance.

Sequencing depth

Sample	Total	Mapped	Uniq	Uniq_rmdup
NP9_Ab3_S87_L007	19866418	19866418	15868814	9645996
NP9_Ab1_S85_L007	57711254	57711254	52685958	37079664
NP12_Ab3_S96_L007	25532480	25532480	20503512	11192352
NP12_Ab1_S94_L007	52835116	52835116	48055420	31104630
NP11_Ab3_S93_L007	29053582	29053582	23678142	13746254
NP11_Ab1_S91_L007	48942940	48942940	45206140	27359510

NP10_Ab3_S90_L007	20953976	20953976	16996738	9632080
NP10_Ab1_S88_L007	47721170	47721170	44031230	29971312
AB3_616_S317_L008.hg19	8245278	8245278	7433188	6695886
AB3_560_S314_L008.hg19	26827434	26827434	23497930	16380416
AB3_532_S311_L008.hg19	8114900	8114900	6938636	5643530
AB3_505_S305_L008.hg19	34784472	34784472	30377284	20657938
AB3_484_S302_L008.hg19	33350630	33350630	28951346	18496882
AB3_461_S299_L008.hg19	26857860	26857860	23361854	14624180
AB3_442_S296_L008.hg19	37210520	37210520	32506086	22288486
AB3_258_S290_L008.hg19	42123454	42123454	37780614	26419280
AB1_616_S315_L008.hg19	91552248	91552248	82976924	68684064
AB1_560_S312_L008.hg19	68281038	68281038	63533174	46378316
AB1_532_S309_L008.hg19	52470684	52470684	48773932	37055916
AB1_505_S303_L008.hg19	52321356	52321356	48464796	36140496
AB1_484_S300_L008.hg19	68549140	68549140	63506122	46679568
AB1_461_S297_L008.hg19	83648770	83648770	78070302	56543966
AB1_442_S294_L008.hg19	64545394	64545394	60166028	46329980
AB1_258_S289_L008.hg19	53893632	53893632	48905488	38664050
777_Ab3_S60_L006	50708582	50708582	40650460	22706638
777_Ab1_S58_L006	16929191	16929191	15456440	12721900
776_Ab3_S57_L006	21193198	21193198	16907920	5613482
776_Ab1_S55_L006	30633804	30633804	27846294	20920998
774_Ab3_S54_L006	35527264	35527264	28461780	14767282
774_Ab1_S52_L006	82663896	82663896	75992910	52618000
768_Ab3_S51_L006	28168916	28168916	22528060	12121214
768_Ab1_S49_L006	68262890	68262890	62697190	41046830
767_Ab3_S48_L006	32504954	32504954	25908914	14983890
767_Ab1_S46_L006	74452148	74452148	68121012	47211176
760_Ab3_S45_L006	29559926	29559926	23627974	13313314
760_Ab1_S43_L006	71659516	71659516	65981780	46741786
757_Ab3_S42_L006	29565438	29565438	23425442	12834146
757_Ab1_S40_L006	63853158	63853158	57939124	35629596
755_Ab3_S39_L006	11586264	11586264	8616416	6041062
755_Ab1_S37_L006	96359626	96359626	89343340	54376490
213_Ab3_S84_L007	16885802	16885802	13264014	8415582
213_Ab1_S82_L007	46989992	46989992	43595172	29954726
210_Ab3_S81_L007	24149912	24149912	19310392	12345520
210_Ab1_S79_L007	59709758	59709758	55708620	38641768
206_Ab3_S78_L007	28075504	28075504	22045876	13307778
206_Ab1_S76_L007	42827876	42827876	39576282	26103516
203_Ab3_S75_L007	27583326	27583326	22194356	12487234
203_Ab1_S73_L007	46490036	46490036	42012662	30122714
198_Ab3_S72_L007	25744320	25744320	20485122	11497700
198_Ab1_S70_L007	47273522	47273522	43992858	27933962
196_Ab3_S69_L007	29431918	29431918	23753810	14449562
196_Ab1_S67_L007	54338710	54338710	50099046	37942680
194_Ab3_S66_L007	14760936	14760936	11782552	6423390
194_Ab1_S64_L007	43689880	43689880	40270670	28094118
192_Ab3_S63_L007	19058722	19058722	15374416	8843190
192_Ab1_S61_L007	44078694	44078694	40432376	27672254
H_1_K4_WT_S12_L005.hg19.sorted	66198404	66198404	62103880	38662184
H_2_K4_WT_S13_L005.hg19.sorted	64256844	64256844	60109754	39015904
H_3_K4_WT_S14_L005.hg19.sorted	61761062	61761062	58652322	36741662
H_1_K4_KO_S19_L005.hg19.sorted	73915214	73915214	68481028	43454550
H_2_K4_KO_S20_L005.hg19.sorted	52167598	52167598	48798922	30729710
H_3_K4_KO_S21_L005.hg19.sorted	90769394	90769394	83526186	64615194
H_1_DD_WT_S15_L005.hg19.sorted	76788700	76788700	71557166	48656610
H_2_DD_WT_S16_L005.hg19.sorted	69797890	69797890	64501764	42365234
H_3_DD_WT_S17_L005.hg19.sorted	66075710	66075710	60832878	41868458
H_1_DD_KO_S22_L005.hg19.sorted	34392416	34392416	30750956	23307316
H_2_DD_KO_S23_L005.hg19.sorted	37920920	37920920	34153444	24321770
H_3_DD_KO_S24_L005.hg19.sorted	71437646	71437646	66810286	43626434
H_1_IgG_WT_S18_L005.hg19.sorted	34406156	34406156	30151962	22106604
H_1_IgG_KO_S25_L005.hg19.sorted	25183044	25183044	21935454	6032818
921_dop_S106_L006.mm10	10668796	10668796	9597088	8298318
927_dop_S110_L006.mm10	7979140	7979140	7216314	6320654
932_dop_S112_L006.mm10	14204920	14204920	12785184	11046402
937_dop_S113_L006.mm10	14911758	14911758	12934434	11238654
941_dop_S115_L006.mm10	15031270	15031270	13588674	11540598
914_dop_S104_L006.mm10	12659676	12659676	11264688	9546488
915_dop_S105_L006.mm10	19226126	19226126	17371800	14341418
922_dop_S107_L006.mm10	11916982	11916982	10671686	9211250
939_dop_S114_L006.mm10	12210914	12210914	10927692	9277756
945_dop_S116_L006.mm10	10141844	10141844	8726612	7447372
904_dop_S103_L006.mm10	19706748	19706748	17496534	14838062
923_dop_S108_L006.mm10	16554748	16554748	14871398	12547778

925_dop_S109_L006.mm10 5822902 5822902 5079710 4348114
930_dop_S111_L006.mm10 12307798 12307798 10930914 9365426
946_dop_S117_L006.mm10 12911000 12911000 11388312 9618166
Group1_igg_S118_L006.mm10 2548370 2548370 2022146 1732692
Group3_igg_S120_L006.mm10 5206846 5206846 4129838 3415282
Group2_igg_S119_L006.mm10 3140426 3140426 2507550 2140078

Antibodies

H3K4me3 (Active Motif, 39159), H3K4me3Q5dopaminyl (Millipore, ABE2590), or rabbit IgG (Invitrogen, 10500c)

Peak calling parameters

macs2 callpeak -t file_uniq_rmdup.bam -c igg_uniq_rmdup.bam -f BAMPE -g mm (or hh) --call-summits --outdir out_dir_path -n file_name

Data quality

MACS2 peak calling was used with the corresponding IgG control, with a threshold of $q < 0.05$. Peaks were visually inspected in IGV and by heatmap.

Software

Raw fastq files were aligned to the hg19 or mm10 genome using bowtie2 (v2.5.0). Low-quality reads were filtered using Samtools (v.1.9) with a MAPQ cut-off score of 30. Only unique, deduplicated reads were retained for further processing. Bigwig files were produced using the deepTools package (v.3.5.1), using an ENCODE hg19 or mm10 v2 blacklist file to discard regions with consistently non-specific signal, and scaled using E. coli spike-in controls to normalize sequencing depth. To determine normalization factors based on E. coli reads, each sample was aligned to the E. coli genome (MG1655), and the unique deduplicated reads were compared across groups per antibody per experiment. The sample with the lowest number of E. coli reads was determined ("minimum"), and all samples were scaled by dividing their corresponding E. coli read count by this minimum number. For each group, bigwig files were merged and peak callling was conducted using MACS2 (v2.1.0) with the corresponding merged IgG file as control, filtered for peaks with FDR < 0.05. Peak annotation was conducted using HOMER (v4.1.1). Heatmaps were made either using the DiffBind (v3.8.4) or deepTools (v3.5.5) packages. For deepTools, heatmaps were made by merging DEGs from RNA-seq data with TSSs downloaded from the UCSC genome browser using the canonically annotated transcript for each gene. Profiles were generated and statistically analyzed using the deepStats package by using the dsCompareCurves function to perform Wilcoxon Rank-sum tests per-bin. For DiffBind analysis, heatmaps were made for peaks identified by DiffBind's differential peak algorithm, where differential peaks were first filtered using a log2(fold change) threshold > 0.1 and defined at $p < 0.05$, where log2(fold change) was calculated as log2(parity) – log2(NP), based on prior empirical observations used to define thresholds for differential peaks. ChEA analysis on annotated loci was conducted using EnrichR with a significance threshold of adjusted $p < 0.05$.

Flow Cytometry

Plots

Confirm that:

- [ ] The axis labels state the marker and fluorochrome used (e.g. CD4-FITC).
- [ ] The axis scales are clearly visible. Include numbers along axes only for bottom left plot of group (a 'group' is an analysis of identical markers).
- [ ] All plots are contour plots with outliers or pseudocolor plots.
- [ ] A numerical value for number of cells or percentage (with statistics) is provided.

Methodology

Sample preparation

Describe the sample preparation, detailing the biological source of the cells and any tissue processing steps used.

Instrument

Identify the instrument used for data collection, specifying make and model number.

Software

Describe the software used to collect and analyze the flow cytometry data. For custom code that has been deposited into a community repository, provide accession details.

Cell population abundance

Describe the abundance of the relevant cell populations within post-sort fractions, providing details on the purity of the samples and how it was determined.

Gating strategy

Describe the gating strategy used for all relevant experiments, specifying the preliminary FSC/SSC gates of the starting cell population, indicating where boundaries between "positive" and "negative" staining cell populations are defined.

- [ ] Tick this box to confirm that a figure exemplifying the gating strategy is provided in the Supplementary Information.

Magnetic resonance imaging

Experimental design

Design type

Indicate task or resting state; event-related or block design.

Design specifications

Specify the number of blocks, trials or experimental units per session and/or subject, and specify the length of each trial or block (if trials are blocked) and interval between trials.

Behavioral performance measures | State number and/or type of variables recorded (e.g. correct button press, response time) and what statistics were used to establish that the subjects were performing the task as expected (e.g. mean, range, and/or standard deviation across subjects).

Acquisition

Imaging type(s) | Specify: functional, structural, diffusion, perfusion.

Field strength | Specify in Tesla

Sequence & imaging parameters | Specify the pulse sequence type (gradient echo, spin echo, etc.), imaging type (EPI, spiral, etc.), field of view, matrix size, slice thickness, orientation and TE/TR/flip angle.

Area of acquisition | State whether a whole brain scan was used OR define the area of acquisition, describing how the region was determined.

Diffusion MRI ☐ Used ☐ Not used

Preprocessing

Preprocessing software | Provide detail on software version and revision number and on specific parameters (model/functions, brain extraction, segmentation, smoothing kernel size, etc.).

Normalization | If data were normalized/standardized, describe the approach(es): specify linear or non-linear and define image types used for transformation OR indicate that data were not normalized and explain rationale for lack of normalization.

Normalization template | Describe the template used for normalization/transformation, specifying subject space or group standardized space (e.g. original Talairach, MNI305, ICBM152) OR indicate that the data were not normalized.

Noise and artifact removal | Describe your procedure(s) for artifact and structured noise removal, specifying motion parameters, tissue signals and physiological signals (heart rate, respiration).

Volume censoring | Define your software and/or method and criteria for volume censoring, and state the extent of such censoring.

Statistical modeling & inference

Model type and settings | Specify type (mass univariate, multivariate, RSA, predictive, etc.) and describe essential details of the model at the first and second levels (e.g. fixed, random or mixed effects; drift or auto-correlation).

Effect(s) tested | Define precise effect in terms of the task or stimulus conditions instead of psychological concepts and indicate whether ANOVA or factorial designs were used.

Specify type of analysis: ☐ Whole brain ☐ ROI-based ☐ Both

Statistic type for inference | Specify voxel-wise or cluster-wise and report all relevant parameters for cluster-wise methods.

(See Eklund et al. 2016)

Correction | Describe the type of correction and how it is obtained for multiple comparisons (e.g. FWE, FDR, permutation or Monte Carlo).

Models & analysis

n/a	Involved in the study
☐	☐ Functional and/or effective connectivity
☐	☐ Graph analysis
☐	☐ Multivariate modeling or predictive analysis

Functional and/or effective connectivity | Report the measures of dependence used and the model details (e.g. Pearson correlation, partial correlation, mutual information).

Graph analysis | Report the dependent variable and connectivity measure, specifying weighted graph or binarized graph, subject- or group-level, and the global and/or node summaries used (e.g. clustering coefficient, efficiency, etc.).

Multivariate modeling and predictive analysis | Specify independent variables, features extraction and dimension reduction, model, training and evaluation metrics.
