# Supplementary material for: Dopamine drives persistent remodelling of the maternal brain
Source: Nature. 2026 May 20;654(8118):465–75. doi: 10.1038/s41586-026-10509-4 (PMC13253353; doi:10.1038/s41586-026-10509-4)
Supplement: Supplementary file 4 — Peer Review File [file 41586_2026_10509_MOESM4_ESM.pdf]

nature portfolio

Peer Review File

Dopamine drives persistent remodeling of the maternal brain

Corresponding Author: Dr Ian Maze

Any redactions in this file are there to maintain patient confidentiality, the confidentiality of unpublished data, or to remove third-party material.

This file contains all reviewer reports in order by version, followed by all author rebuttals in order by version.

Attachments originally included by the reviewers as part of their assessment can be found at the end of this file.

Version 0:

Reviewer comments:

Referee #1

(Remarks to the Author)
In this work, Chan and colleagues investigate the impact of parity on the maternal brain in mice and also performed analysis using human brain tissue to confirm the effects observed in the animal model. The authors used an impressively large set of sophisticated orthogonal and complementary in vivo and in vitro approaches, including meticulous mice behavior studies, multiple rounds of brain gene expression and transcriptomic analyses and single nuclei RNA sequencing, to show that pregnancy and postpartum stress induce transcriptional changes specifically in the dorsal hippocampal formation (dHF). The authors have identified a set of genes that display persistent and progressive downregulation during postpartum. These genes are associated with dopaminergic signaling, and their expression depends on dopamine dynamics and dopaminylation of histone H3.

Overall, this is an exceptionally challenging study that addresses the fundamental long sought after question regarding the mechanism by which parity promotes long-lasting transcriptional and behavioral adaptations in brain. This manuscript contains exceptional quality data, conclusions are convincing and justified, and I enthusiastically support publication.

Referee #3

(Remarks to the Author)
The manuscript “Persistent dopamine-dependent remodeling of the neural transcriptome in response to pregnancy and postpartum” provides a deep analysis into the molecular changes that may underly the lasting neurobiological effects of pregnancy and postpartum experiences in mice. The molecular tools used to explore these effects identify the dorsal hippocampal formation (dHF) brain region as a key target that differentiates females that have vs. have not undergone the experience of pregnancy and postpartum and further interrogates the dependency of dopamine-associated histone changes in the molecular and behavioral profiles observed. The manuscript also examines the impact of postnatal stress on some of these outcomes and suggests that this experience shifts phenotypes (molecular and behavioral) to a state intermediate between parous and nulliparous. Overall, this work does add significant insights into the long-term effects of pregnancy and postpartum experiences on the brain – a question that has been increasingly explored using human neuroimaging approaches.

Comments/critiques
1) The primary comparison that is the focus of this manuscript is between NP vs. PP. While there is subsequent analyses attempting to differentiate the potential impact of a more nuanced comparison (i.e pregnancy only, mating only, pup sensitization), there are limits to the effectiveness of these approaches that need to be more carefully acknowledged in the interpretation of the findings. The PP group includes mating, pregnancy, vaginal birth, lactation, postnatal mother-infant interactions, and weaning of offspring. Thus is can’t be concluded that “parity”, “the state of previously carrying a pregnancy to term” is the variable defining the PP group molecular and behavioral phenotype. More care needs to be taken in acknowledging this limitation or reframing what the data are illustrating. The supplementary groups created to explore

different aspects of the PP group have notable limitations. Mating that achieves a successful pregnancy may be a very different experience than mating that does not. The pregnancy only group also gave birth to live pups which may trigger lasting changes in the brain and physiology. While pup sensitization does allow for interactions with pups, these experiences are unlikely mimic the full array of postnatal interactions that are typical of the PP experience. Lactation seems not to be considered fully in this design and will likely have a significant effect on the brain and behavior. Thus the PP group is not restricted to "parity" and the interpretation of the findings must account for this lack of equivalency. This may be particularly important for the selection of the dHF. If a more restrictive definition of parity was used, would this brain region still emerge as strongly?
2) Given the NP vs. PP differences in cell type estimates, why was this variable not used as a covariate when examining DEGs? The DEG counts should factor in the potential confound of group differences in cell types.
3) The link between behavior and transcriptional changes is highly speculative – these were assessed in different animals and so a direct link can't be made – though the subsequent studies that manipulate key systems and examine behavior do attempt to lessen the impact of this limitation.
4) While assessing estrus state at the time of behavioral testing is a strength of the approach, the conclusions drawn from this approach need to be carefully considered. The authors need to report how many females in each analysis were in each estrus state and whether there was sufficient power to detect differences – which based on the data visualizations, is doubtful. Using estrus state as a covariate is reasonable. Concluding that estrus state does not impact behavior is unlikely to be reasonable unless the numbers per group are very high.
5) Why is chronic stress being done so late in the postpartum period? I understand the desire to avoid potential mortality issues but by avoiding critical times of hormonal, lactational, and behavior experience during the early postpartum period, there is a missed opportunity to model experiences during this critical postpartum timepoint.
6) Lines 304-305; It unclear how "This postpartum stress paradigm resolves key windows during which disruptions to parity adaptations occur," – as per the comment above, it is clear that key windows are being missed based on the timing of implementation.
7) Line 370-371: "Similarly, increased release of dopamine in dHF tissues was not observed in NP females 30-minutes following separation from a littermate (Extended Data S4p), though this likely lacks the same salience as pup separation." This is not a good comparison and weakens the manuscript.
8) On what basis do the authors conclude that there is a "prevailing view that maternal dopamine function is solely driven by transient surges during maternal behaviors". There is an extensive literature examining the role of dopamine in the brain of females during pregnancy and the postpartum period that is not focused on mother-infant interactions.
9) Why was litter size not used as a covariate?

Version 1:

Reviewer comments:

Referee #3

(Remarks to the Author)
The revised manuscript "Persistent dopamine-dependent remodeling of the neural transcriptome in response to pregnancy and postpartum" thoroughly addresses the critiques raised and represents a significant contribution to the literature on the lasting effects of reproductive experiences on the brain. This methodologically sophisticated and robust work that carefully frames the results.

Referee #4

(Remarks to the Author)
The manuscript by Chan et al is remarkable in its depth, range of techniques, and quite timely – determining genetic and epigenetic based neural plasticity in the hippocampal formation during pregnancy and post-partum. As the authors show via behavioral studies, and cite in extensive literature, across the mammalian kingdom pregnancy permanently changes the brain and alters the expression of future behaviors of the mother. Despite the centrality of pregnancy to the human experience, how it changes the brain remains poorly defined. Taking an unbiased gene expression approach across 11 brain regions previously linked to parity, the authors landed on the dorsal hippocampal formation (dHF) because it demonstrated the largest number of differentially expressed genes (with most being downregulated). They then show that the state of full-term pregnancy plus a full-term postpartum period (RE) drives the greatest transcriptional changes in the dHF. Adding a maternal stress paradigm predictably altered the gene expression signature and behaviors in the dHF compared to pregnancy + postpartum and no stress. The scRNAseq data was a nice touch and allowed them to key in on dopamine signaling, and they showed bi-directional effects of DA levels with pup separation (DA up) vs normal pregnancy + postpartum (DA down). The paper then ends showing that the downregulated DA signaling between the VTA and dHF results in a downregulation of H3 dopaminylation in both mice and humans with one or more pregnancies. They prove causality of this result with chemogenetics and add-back experiments with the H3 dopamine epigenetic marker. In all, I enjoyed this paper, it was easy to follow and well-written, and I am convinced by the results and impact of the work. I endorse publication.

Here are a few minor comments the authors can consider:

-RNA in situs in the dorsal CA1 are not convincing and don't seem to fully align with the neighboring scRNAseq clustering of the respective genes across states (Drd1, Drd2).

-In the figure3 maternal stress paradigm, would a stress-relieving drug, or even providing some pup cues (vocalizations/odors) rescue the behavior and gene expression changes associated with the separation?
-I could not tell if there was general convergence between the bulk-seq data (which is most of the paper) vs the scRNAseq data? Are the same genes generally up/down. A table or vinn diagram showing overlap would be useful – especially given the technical difficulties of inferring differential gene expression across groups from scRNAseq data.

Open Access This Peer Review File is licensed under a Creative Commons Attribution 4.0 International License, which permits use, sharing, adaptation, distribution and reproduction in any medium or format, as long as you give appropriate credit to the original author(s) and the source, provide a link to the Creative Commons license, and indicate if changes were made.
In cases where reviewers are anonymous, credit should be given to 'Anonymous Referee' and the source.
The images or other third party material in this Peer Review File are included in the article's Creative Commons license, unless indicated otherwise in a credit line to the material. If material is not included in the article's Creative Commons license and your intended use is not permitted by statutory regulation or exceeds the permitted use, you will need to obtain permission directly from the copyright holder.
To view a copy of this license, visit https://creativecommons.org/licenses/by/4.0/

Response to Referees for O'Chan *et al.*, 2025: *Persistent dopamine-dependent remodeling of the neural transcriptome in response to pregnancy and postpartum*

Referees' comments:

Referee #1 (Remarks to the Author):

In this work, Chan and colleagues investigate the impact of parity on the maternal brain in mice and also performed analysis using human brain tissue to confirm the effects observed in the animal model. The authors used an impressively large set of sophisticated orthogonal and complementary in vivo and in vitro approaches, including meticulous mice behavior studies, multiple rounds of brain gene expression and transcriptomic analyses and single nuclei RNA sequencing, to show that pregnancy and postpartum stress induce transcriptional changes specifically in the dorsal hippocampal formation (dHF). The authors have identified a set of genes that display persistent and progressive downregulation during postpartum. These genes are associated with dopaminergic signaling, and their expression depends on dopamine dynamics and dopaminylation of histone H3.

Overall, this is an exceptionally challenging study that addresses the fundamental long sought after question regarding the mechanism by which parity promotes long-lasting transcriptional and behavioral adaptations in brain. This manuscript contains exceptional quality data, conclusions are convincing and justified, and I enthusiastically support publication.

We sincerely thank the Referee for their supportive assessment of our manuscript. We are pleased that they found our study to be rigorous, novel, and impactful, and we appreciate their endorsement of publication.

Referee #3 (Remarks to the Author):

The manuscript "Persistent dopamine-dependent remodeling of the neural transcriptome in response to pregnancy and postpartum" provides a deep analysis into the molecular changes that may underly the lasting neurobiological effects of pregnancy and postpartum experiences in mice. The molecular tools used to explore these effects identify the dorsal hippocampal formation (dHF) brain region as a key target that differentiates females that have vs. have not undergone the experience of pregnancy and postpartum and further interrogates the dependency of dopamine-associated histone changes in the molecular and behavioral profiles observed. The manuscript also examines the impact of postnatal stress on some of these outcomes and suggests that this experience shifts phenotypes (molecular and behavioral) to a state intermediate between parous and nulliparous. Overall, this work does add significant insights into the long-term effects of pregnancy and postpartum experiences on the brain – a question that has been increasingly explored using human neuroimaging approaches.

We very much appreciate the Referee's positive evaluation of our manuscript and their recognition that this work adds significant insights into the long-term effects of pregnancy and postpartum experiences on the brain. Please find our detailed responses to their individual critiques below.

Comments/critiques

1) The primary comparison that is the focus of this manuscript is between NP vs. PP. While there is subsequent analyses attempting to differentiate the potential impact of a more nuanced

comparison (i.e pregnancy only, mating only, pup sensitization), there are limits to the effectiveness of these approaches that need to be more carefully acknowledged in the interpretation of the findings. The PP group includes mating, pregnancy, vaginal birth, lactation, postnatal mother-infant interactions, and weaning of offspring. *Thus is can't be concluded that "parity", "the state of previously carrying a pregnancy to term" is the variable defining the PP group molecular and behavioral phenotype. More care needs to be taken in acknowledging this limitation or reframing what the data are illustrating.* The supplementary groups created to explore different aspects of the PP group have notable limitations. Mating that achieves a successful pregnancy may be a very different experience than mating that does not. The pregnancy only group also gave birth to live pups which may trigger lasting changes in the brain and physiology. While pup sensitization does allow for interactions with pups, these experiences are unlikely mimic the full array of postnatal interactions that are typical of the PP experience. Lactation seems not to be considered fully in this design and will likely have a significant effect on the brain and behavior. Thus the PP group is not restricted to "parity" and the interpretation of the findings must account for this lack of equivalency. This may be particularly important for the selection of the dHF. If a more restrictive definition of parity was used, would this brain region still emerge as strongly?

*We thank the Referee for their positive insights and thoughtful suggestions. As noted by the Referee, neuroimaging studies have increasingly examined the long-term effects of pregnancy experiences on human brain structure and connectivity, yet relatively few studies have investigated the underlying molecular mechanisms contributing to these outcomes. To parallel these human studies in a rodent model, we compared nulliparous (NP) and primiparous (PP) females. Our design was modeled, in part, after seminal work, such as Hoekzema et al. (2017), which prospectively compared women before and after their first pregnancy. In that study, the authors defined nulliparous and primiparous states broadly: primiparity was defined to encompass a combination of fertilization (both natural and in vitro), term pregnancy, birth (vaginal and C-section), and caregiving via breastfeeding and/or feeding with formula. Similarly, de Lange et al. (2020) defined parity based on the number of births. While the literal definition of parity refers to having carried a pregnancy to term, many studies in the clinical literature have similarly defined parity by the number of births/deliveries, linking it to outcomes such as obstetric complications and perinatal mortality (Bai et al., Am J Obstet Gynecol, 2002), cardiovascular disease (Li et al., Eur J Prev Cardiol, 2019), and cervical cancer (Tekalegn et al., Women's Health, 2022). In line with these precedents, our rodent PP group incorporated breeding, pregnancy, birth, nursing, and pup rearing until weaning (at which point pups were removed in accordance with animal care standards). We recognize, however, that despite such vernacular usage of the term "parity," the definition does not reflect the full scope of experiences included in the condition described in our manuscript. Accordingly, in our revised manuscript, we summarize this rationale (**lines 58-65**) and have replaced references to "(primi)parity" with "reproductive experiences" (RE) to more accurately reframe our data as reflecting the composite of maternal experiences described above.*

Regarding the supplementary groups, we agree that each has inherent limitations, and we have revised our manuscript to more clearly acknowledge these caveats. Isolation of individual reproductive experiences is experimentally challenging, and while our intent was to separate these events as faithfully as possible, significant constraints remain. For example, creating a true "Pregnancy Only" group would require in vitro fertilization followed by a procedure to terminate the pregnancy in order to prevent live birth, a manipulation that would inappropriately introduce significant confounds for brain outcomes; therefore, we do not consider this to be a feasible experiment for inclusion in our study. Likewise, although lactation is a critical factor in shaping the maternal brain, it cannot be fully manipulated experimentally: inducing lactation in

*NP females is not technically feasible, and preventing lactation in dams without pup removal would require invasive procedures, such as mammary gland excision, which would themselves present significant confounds in our study by most certainly impacting brain outcomes. As such, and in accordance with the Referee's suggestions, we now discuss these limitations more explicitly in the revised manuscript in both the Results and Discussion sections (**lines 207-212; 669-676**). We have also updated the terminology used for the supplementary groups to more accurately reflect the experiences involved (e.g., "Mating only" → "Mating + No Pregnancy"; "Pregnancy only" → "Mating + Pregnancy + Birth").*

Together, these revisions clarify that our study investigates the long-term molecular and behavioral consequences of composite female "reproductive experiences," providing novel insights into the dynamic and persistent alterations that shape the maternal brain. We believe that such reframing will better reflect both the human contexts described in the literature and the rodent experiments described here. Further, these adjustments more robustly emphasize that the dramatic transcriptional changes observed in the dHF arise from an integrated set of reproductive and maternal exposures, while also acknowledging important limitations that future studies may aim to address by attempting to disentangle these individual contributions.

2) Given the NP vs. PP differences in cell type estimates, why was this variable not used as a covariate when examining DEGs? The DEG counts should factor in the potential confound of group differences in cell types.

In our original analyses, we used RUVSeq to estimate factors of unwanted variation (k), based on the rationale that variation attributable to cell-type composition would be partially captured within these factors. To avoid overfitting in a dataset of modest sample size, we therefore did not initially include explicit cell-type covariates.

Following the Referee's recommendation, however, we have now performed an additional analysis incorporating cell-type surrogate proportion variables (SPVs) derived from our BRETIGEA analyses. Because the six SPVs represent compositional estimates whose values are inherently correlated (e.g., an increase in neuronal proportion necessarily corresponds to a decrease in non-neuronal signatures), including all six SPVs directly in the design matrix would introduce substantial multicollinearity. To address this, we conducted a principal components analysis (PCA) on the scaled SPV matrix to obtain a parsimonious set of orthogonal covariates. This PCA-based approach has been shown to improve detection of cell-intrinsic differential expression while preserving degrees of freedom and improving model stability (Gural et al, 2025).

*In the updated DESeq2 model, we reduced the number of RUVSeq factors (k) by two and incorporated SPV-derived PC1 and PC2 as covariates, where PC1 and PC2 captured the dominant cell composition-related variance across samples. Incorporating these covariates increased the number of DEGs identified in the dHF to 1,567 (**Extended Data 1g**). Notably, consistent with the Reviewer's expectation that adjusting for composition would reveal additional biologically meaningful transcriptional differences, cell composition adjustments in our model realigned the intrinsic "transcriptional sensitivity" of several brain regions, including the LC (from 4th to 2nd) and the NAc (from 2nd to 7th), for example, indicating that earlier rankings were partly driven by composition-related variability and that adjusting for these effects provides a more accurate interpretation of which regions are intrinsically most responsive to RE. Importantly, we were excited to observe that the dHF remained the most transcriptionally responsive brain region to RE even after adjusting for cell composition, indicating that RE drives both shifts in cellular makeup and robust gene expression changes within cell populations themselves. This is*

supported by our snRNA-seq data, which independently reveal alterations in cell-type proportions as well as transcriptional remodeling within specific cell populations following RE.

*Importantly, comparison of the RUVSeq-based and SPV-adjusted analyses in the dHF showed strong concordance at the level of biological pathway enrichment, indicating that the original DESeq2 model successfully identified the major transcriptional programs engaged by RE (**Extended Data 1h-i**). Thus, even without explicit cell-composition covariates, we believe the original analyses provide a robust and biologically valid representation of brain region transcriptional sensitivity. Moreover, because our overarching goal was to characterize both intrinsic gene expression changes and composition-related shifts that identify brain regions most sensitive to RE, we have retained the original RUVSeq-based analyses in the main text and now include the SPV-adjusted analyses in **Extended Data 1** and the list of adjusted DEGs in **Supplementary Table 2**.*

3) The link between behavior and transcriptional changes is highly speculative – these were assessed in different animals and so a direct link can't be made – though the subsequent studies that manipulate key systems and examine behavior do attempt to lessen the impact of this limitation.

*In line with the Referee's comment, we acknowledge that the link between behavior and transcriptional changes observed in our original submission remained correlative, as behavioral and molecular measures were generally obtained from different cohorts, with the exception of the DREADD experimental cohort (**Fig. 6**). Therefore, to further strengthen causal links, we infused an AAV -H3.3Q5A virus (vs. H3.3WT) into the dHF of Control and Stress RE dams, and evaluated both transcriptional and behavioral consequences within the same mice. Our prior published work has established that this approach effectively reduces H3Q5 dopaminylation, alters dopaminylation-dependent transcriptional programs, and leads to corresponding behavioral changes (Lepack et al.,* Science*, 2020; Fulton et al.,* Neuropsychopharmacology*, 2022). Therefore, our rationale in this revised manuscript was to reduce the elevated H3Q5 dopaminylation observed in Stress RE dHF (as shown in **Fig. 5**), reverse the RE-dependent gene expression patterns (as shown in **Fig. 3**), and test whether this manipulation can subsequently rescue postpartum stress-induced behavioral deficits.*

*These new data are now presented in **Fig. 7**. In this new Figure, we show that H3.3Q5A expression in Stress RE dHF: 1) reverses H3Q5 dopaminylation in Stress RE dHF to Control RE-like levels, 2) reverses gene expression to mimic Control RE profiles, and 3) rescues both acquisition and recall of contextual fear conditioning. Together, we believe that these results directly address the limitation raised by the Referee by providing causal evidence that dHF transcriptional adaptations drive the behavioral phenotypes in our model, while also providing a translational context for reversing the effects of postpartum stress.*

4) While assessing estrus state at the time of behavioral testing is a strength of the approach, the conclusions drawn from this approach need to be carefully considered. The authors need to report how many females in each analysis were in each estrus state and whether there was sufficient power to detect differences – which based on the data visualizations, is doubtful. Using estrus state as a covariate is reasonable. Concluding that estrus state does not impact behavior is unlikely to be reasonable unless the numbers per group are very high.

We thank the Referee for bringing up this point. Our intent was not to suggest that estrus stage has no impact on behavior, but rather to clarify that the distribution of estrus stages did not account for the behavioral differences observed between NP and Control RE groups. In the

*revised manuscript, we have clarified this point and provide estrus staging for each animal per behavior in the Source Data table, along with Fisher's exact tests to demonstrate that stage distributions do not differ between groups for each behavioral test (**Extended Data Table 1**).*

While our studies were not powered to detect estrus stage effects per behavioral test - an analysis that would have required at least 4x as many animals per group - this decision was guided by prior work showing no significant impacts of estrus stage on acquisition of contextual fear conditioning (Cossio et al., Physiol Behav*, 2016; Blair et al.,* Psychoneuroendocrinology*, 2022). Furthermore, although pup retrieval has not been directly tested across estrus stages, earlier work found that estrogen depletion via ovariectomy did not alter retrieval success (Rosenblatt,* Science*, 1967).*

*To improve statistical power in our study and strengthen our conclusions, we have further stratified animals into high-estrogen (proestrus + estrus) and low-estrogen (metestrus + diestrus) conditions, following an approach commonly used in the field. Applying this approach, we reanalyzed our behavioral data from the mCherry-infected controls of our DREADD study cohort (Fig. 6), which is sufficiently powered to support group stratification (thereby enabling robust detection of differences), and evaluated the effects of Control RE versus NP across behavioral tests. These results are now included in **Extended Data 8e-f** in the revised manuscript to support our conclusions that behavioral differences reflect reproductive experience and not estrus stage.*

5) Why is chronic stress being done so late in the postpartum period? I understand the desire to avoid potential mortality issues but by avoiding critical times of hormonal, lactational, and behavior experience during the early postpartum period, there is a missed opportunity to model experiences during this critical postpartum timepoint.

*This is an excellent suggestion. In our revised manuscript, we now include new data from an early postpartum stress group (Early Stress RE), in which maternal separation and limited nesting were conducted from postpartum days 2–12, compared to Control RE and NP females. We have conducted the same behavioral and bulk transcriptional analyses of this group as were used for the late postpartum stress cohort. These new results, now presented in **Extended Data 4**, show that chronic stress during the early postpartum period disrupts dHF transcriptional and behavioral adaptations in a manner that closely parallels the effects observed in the late postpartum stress cohort. Because the behavioral and transcriptional outcomes were more robust in the late postpartum stress group, we centered the manuscript on this timing and paradigm for mechanistic clarity. Nonetheless, the early postpartum analyses strengthen the overall conclusion that stress across early and late postpartum windows can mitigate RE-dependent dHF transcriptional and behavioral plasticity.*

6) Lines 304-305; It unclear how "This postpartum stress paradigm resolves key windows during which disruptions to parity adaptations occur," – as per the comment above, it is clear that key windows are being missed based on the timing of implementation.

*As noted in our prior response, we have now included data from an earlier postpartum stress group to address this concern (see **Extended Data 4**) and have removed this sentence from the manuscript.*

7) Line 370-371: "Similarly, increased release of dopamine in dHF tissues was not observed in NP females 30-minutes following separation from a littermate (Extended Data S4p), though this

likely lacks the same salience as pup separation.” This is not a good comparison and weakens the manuscript.

Based on the Referee’s recommendation, we agree that this comparison does not meaningfully strengthen the manuscript and have removed these data from the Figure.

8) On what basis do the authors conclude that there is a “prevailing view that maternal dopamine function is solely driven by transient surges during maternal behaviors”. There is an extensive literature examining the role of dopamine in the brain of females during pregnancy and the postpartum period that is not focused on mother-infant interactions.

*We thank the Referee for this comment. We agree that dopamine function has been studied extensively during pregnancy and the postpartum period. Our intent was to clarify that, beyond these transient roles, our work demonstrates a persistent dopaminergic contribution to long-lasting maternal brain adaptations. We have revised the text (**lines 653-656**) to clarify this distinction.*

9) Why was litter size not used as a covariate?

We did not use litter size as a covariate in our analyses because this variable is inherently confounded with group, and thus is not statistically appropriate to use as a covariate in NP vs. RE comparisons. Specifically, all NP animals have a litter size of zero, while all RE animals have non-zero litter sizes (≥4). This non-overlap (i.e. perfect separation) makes it statistically impossible to estimate independent effects of RE and litter size in the same model.

*To address the Referee’s question as to whether litter size impacts RE effects, however, we returned to our original NP vs. RE dHF bulk RNA-seq dataset and stratified samples into three groups based on litter size: 0 pups (bin 1; N=6, i.e., NP), 4–6 pups (bin 2; N=3), and 7–8 pups (bin 3; N=3). As noted in the Methods, only animals with litter sizes of 4–10 were included in all studies. Unsupervised hierarchical clustering of DEGs showed that all bin 1 (NP) samples clustered together, while bins 2 and 3 were intermixed (**Extended Data 1j**), indicating that variation in litter size within the RE group does not meaningfully alter the transcriptional signatures observed in the dHF.*

Response to Referees for O'Chan *et al.,* 2026:
Dopamine drives persistent remodeling of the maternal brain

Referees' comments:

Referee #3 (Remarks to the Author):

The revised manuscript "Persistent dopamine-dependent remodeling of the neural transcriptome in response to pregnancy and postpartum" thoroughly addresses the critiques raised and represents a significant contribution to the literature on the lasting effects of reproductive experiences on the brain. This methodologically sophisticated and robust work that carefully frames the results.

We thank the Reviewer for their thoughtful comments, which improved the manuscript. We are pleased that the revisions have addressed their concerns and appreciate their support of its publication.

Referee #4 (Remarks to the Author):

The manuscript by Chan et al is remarkable in its depth, range of techniques, and quite timely – determining genetic and epigenetic based neural plasticity in the hippocampal formation during pregnancy and post-partum. As the authors show via behavioral studies, and cite in extensive literature, across the mammalian kingdom pregnancy permanently changes the brain and alters the expression of future behaviors of the mother. Despite the centrality of pregnancy to the human experience, how it changes the brain remains poorly defined. Taking an unbiased gene expression approach across 11 brain regions previously linked to parity, the authors landed on the dorsal hippocampal formation (dHF) because it demonstrated the largest number of differentially expressed genes (with most being downregulated). They then show that the state of full-term pregnancy plus a full-term postpartum period (RE) drives the greatest transcriptional changes in the dHF. Adding a maternal stress paradigm predictably altered the gene expression signature and behaviors in the dHF compared to pregnancy + postpartum and no stress. The scRNAseq data was a nice touch and allowed them to key in on dopamine signaling, and they showed bi-directional effects of DA levels with pup separation (DA up) vs normal pregnancy + postpartum (DA down). The paper then ends showing that the downregulated DA signaling between the VTA and dHF results in a downregulation of H3 dopaminylation in both mice and humans with one or more pregnancies. They prove causality of this result with chemogenetics and add-back experiments with the H3 dopamine epigenetic marker. In all, I enjoyed this paper, it was easy to follow and well-written, and I am convinced by the results and impact of the work. I endorse publication.

Here are a few minor comments the authors can consider:

-RNA in situs in the dorsal CA1 are not convincing and don't seem to fully align with the neighboring scRNAseq clustering of the respective genes across states (Drd1, Drd2).

*We have updated the representative images (**Figure 2i; Figure 4g**) to better reflect the patterns observed in the snRNA-seq data. Drd1 and Drd2 expression in dorsal CA1 is known to be sparse, and therefore RNAscope signal is limited to a small number of puncta-positive cells. Thus, we have also added insets to more clearly visualize these puncta-positive cells.*

-In the figure3 maternal stress paradigm, would a stress-relieving drug, or even providing some pup cues (vocalizations/odors) rescue the behavior and gene expression changes associated with the separation?

*We thank the Reviewer for this thoughtful suggestion. Testing whether pharmacological treatment or restoration of pup-associated sensory cues can rescue the behavioral and transcriptional effects of maternal separation would provide important insight into the mechanisms underlying these changes. While such experiments are beyond the scope of the current study, we have added discussion regarding this as a future direction (**lines 474-476**).*

-I could not tell if there was general convergence between the bulk-seq data (which is most of the paper) vs the scRNAseq data? Are the same genes generally up/down. A table or vinn diagram showing overlap would be useful – especially given the technical difficulties of inferring differential gene expression across groups from scRNAseq data.

*We have now provided additional data (**Extended Data Figure 7o-q**) summarizing the convergence between the snRNA-seq and bulk RNA-seq datasets. To assess differential expression overlap for both the Control RE vs. NP and Control RE vs. Stress RE comparisons, we compared bulk RNA-seq data from Fig. 2 (formerly Fig. 3) with pseudobulk analyses from Extended Data Figure 7 (formerly Fig. 4). We found that ~16-38% of bulk DEGs overlapped with our pseudobulk analyses when accounting for directionality, consistent with known technical and anatomical differences between datasets, including platform-specific capture methods (poly-A in bulk vs. 3' UMI-based detection in single nuclei), the increased sensitivity of bulk RNA-seq for highly abundant transcripts, and differences in tissue input (a single dHF micropunch for bulk vs. four larger bilateral dHF dissections for snRNA-seq). The overlap was lower when applying an additional fold change threshold of 1.5, consistent with the reduced power of pseudobulk analyses to detect smaller effect size changes. Given that pathway analyses benefit from broader gene lists to detect enrichment, we performed pathway analysis on the overlapping DEGs identified without the fold change threshold and found that similar KEGG pathways were enriched in the overlapping DEGs as in the full bulk DEG analysis (Figure 2d), supporting functional concordance between our datasets.*
